# A Faculty Development Workshop for Planning and Implementing Interactive Virtual Case-Based Teaching

**DOI:** 10.15766/mep_2374-8265.11126

**Published:** 2021-03-17

**Authors:** Jennifer O. Spicer, Trong Tien Nguyen, Margaret W. Arnold, Tiffany Anderson, Roy Khalife

**Affiliations:** 1 Assistant Professor, Department of Medicine, Division of Infectious Diseases, Emory University School of Medicine; 2 Assistant Professor, Department of Medicine, Division of Infectious Diseases, McGill University Faculty of Medicine; 3 Program Director, Department of Surgery, MedStar Health Baltimore; 4 Resident, Department of Surgery, University of Florida College of Medicine; 5 Fellow, Department of Medicine, Division of Hematology, University of Ottawa Faculty of Medicine

**Keywords:** Faculty Development, Cased-Based Learning, Online/Distance Learning, Clinician Educators, Virtual Learning

## Abstract

**Introduction:**

The virtual learning environment has become increasingly important due to physical distance requirements put in place during the COVID-19 pandemic. The transition to a virtual format has been challenging for case-based teaching sessions, which involve substantial audience participation. We developed a faculty development workshop aimed at teaching health professions educators how to use various interactive virtual tools within videoconferencing platforms to facilitate virtual case-based sessions.

**Methods:**

Two 90-minute workshops were piloted as a faculty development initiative. The facilitators demonstrated interactive teaching tools that could be used within virtual case-based sessions. Then, participants discussed how to incorporate these tools into case-based teaching sessions of different class sizes in small-group breakout sessions. Participants completed an online survey following each workshop to evaluate the sessions.

**Results:**

A total of 18 and 26 subjects participated in the first and second workshops, respectively. Survey response rates were 100% (*n* = 18) and 65% (*n* = 17) for the first and second workshops, respectively. Both groups provided overall high ratings and reported that the workshop was clear, organized, and relevant. Participants were more familiar and comfortable with the use of various interactive tools for online teaching.

**Discussion:**

Distance online teaching will be increasingly required for an undetermined time. Faculty development efforts are crucial to facilitate effective interactive teaching sessions that engage learners and maximize learning. This virtual teaching workshop is a simple and straightforward way to introduce a more interactive format to virtual case-based teaching in the health professions.

## Educational Objectives

By the end of this activity, learners will be able to:

1. Explain select learning theories that underpin a multimodal model of online learning.
2. Identify unique features and limitations of various interactive virtual tools.
3. Integrate online tools into interactive case-based virtual teaching sessions.

## Introduction

The virtual learning environment has become increasingly important during the coronavirus disease 2019 (COVID-19) pandemic as physical distancing requirements have required health professions programs to adapt previous in-person sessions to synchronous videoconferencing platforms. Health professions educators transitioned lecture-based sessions to videoconferencing platforms with relative ease, but case-based teaching sessions have presented more of a challenge. Traditional case-based teaching sessions, such as morning report, involve substantial audience participation. Health professions learners and educators believe that active learner engagement in case-based teaching is important for effective learning,^1^ which is consistent with findings from undergraduate science courses demonstrating that active learning strategies increase student performance more than listening to lectures does.^2^ In the virtual setting, this audience interaction can be difficult to facilitate unless educators are familiar and facile with videoconferencing platforms and adjunctive interactive tools.

Even though technology-enhanced learning has become more prevalent in health professions education over the last few decades,^3^ synchronous sessions remain uncommon. A recently published survey of internal medicine residency program directors showed that close to two-thirds of programs in the United States rarely or never used synchronous online electronic learning (e-learning).^4^ A lack of information technology skills was found to be an important barrier to implementation of e-learning in a well-conducted systematic review geared toward health professions education.^5^ Notwithstanding this evidence, Wittich and colleagues' survey also showed how efforts to equip faculty with the knowledge, skills, and attitudes needed for e-learning were generally inadequate or lacking.^4^ Facing the many hurdles of the COVID-19 pandemic, this substantial gap in local institutions' abilities to provide adequate faculty development for e-learning requires additional attention.

Limited information has been published on practical real-world use of videoconferencing platforms to facilitate interactive case-based teaching. Brady and Pradham recently outlined best practices for transitioning in-person sessions to synchronous and asynchronous distance-learning platforms and also summarized the features of various videoconferencing platforms.^6^ In relation to case-based learning, Murdock and colleagues recently shared a short description of some lessons learned from their experience transitioning their morning report to a synchronous videoconferencing platform.^7^ *MedEdPORTAL* has published a workshop describing the development of case-based teaching sessions using evidence-based strategies,^8^ but we were unable to find faculty development resources aimed at teaching faculty how to convert their current case-based teaching sessions to a virtual platform.

Our workshop aims to teach health professions educators how to use videoconferencing platforms to facilitate interactive virtual case-based sessions. The workshop uses Zoom (Zoom Video Communications) as the primary online videoconferencing platform given its prevalence and availability in current health professions education programs; however, materials could be adapted to deliver the same workshop using another videoconferencing platform. The workshop is meant to be implemented virtually in order to demonstrate the virtual teaching tools about which the participants are learning; therefore, participants should have a basic knowledge of videoconferencing platforms (e.g., how to log on and mute/unmute themselves) prior to the session. No other technology-specific knowledge is required ahead of time. Prior to the session, participants are provided with reference materials to familiarize themselves with relevant learning theories. During the session, participants learn about interactive teaching tools within and outside of Zoom, practice using those tools, and then work in small groups to outline how to integrate those tools into a case-based teaching session.

## Methods

### Curricular Context

We initially designed this workshop as a final seminar for our Master of Health Professions Education (MHPE) core course Instruction and Assessment, based at the University of Illinois Chicago, in July 2020. The course culminated in a Residency Day, at which students developed and presented their workshops with opportunity for immediate feedback. The session was a synchronous online session conducted through videoconferencing software (Zoom). Participants included 26 MHPE candidates with various educational roles at their primary institutions. Based on positive feedback, one of the authors (Jennifer O. Spicer) repeated the workshop at the Emory University School of Medicine as a virtual faculty development session for a group of 30 faculty members serving as small-group facilitators for medical students from various clinical departments within the School of Medicine.

### Participant Prerequisites

All participants needed to have at least a basic knowledge of how to use videoconferencing software (e.g., how to log on and mute/unmute themselves) prior to the session but were not required to be familiar with any other functions.

### Materials

Participants were expected to have the following workshop materials and software available:

• Computer with reliable internet access, microphone, and speakers.
• Zoom link (created by tutorial organizers prior to the session and emailed to the learners).
• Word-processing program that could open and edit DOCX files, such as Microsoft Word (Microsoft Corporation), OpenOffice Writer (Apache Software Foundation), Apple Pages (Apple), and so on.
• Optional: pen and paper—participants could write on paper printouts of the workshop documents as an alternative to working on them electronically.

In addition to the above, workshop facilitators and presenters also required the following:

• Free subscriptions (for the presenter) to:
  ○ Zoom (Zoom Video Communications Inc.; https://zoom.us).
  ○ Backchannel Chat (LearnWeaver; http://backchannelchat.com).
  ○ Padlet (WallWisher; https://padlet.com).
  ○ PollEverywhere (PollEverywhere; https://www.polleverywhere.com).
• Subscription to an online polling software to collect participants' postworkshop feedback and evaluation of the session; we used SurveyMonkey (SVMK; https://www.surveymonkey.com), but the survey is portable to other software with the same functionality.
• Workshop document files (see Appendices A–F), which were distributed to participants according to the instructions detailed below.

### Personnel and Training

We ran each workshop session with at least two facilitators, and we strongly recommend this strategy during its implementation elsewhere. One primary facilitator presented the workshop contents and led the discussions. One or more other facilitators assisted with the background logistics, such as monitoring the chat for questions and sharing workshop documents with participants, to ensure a seamless transition between workshop activities. The facilitator demonstrating the interactive teaching tools during the session tested all software ahead of time and rehearsed the workshop to ensure a smooth delivery during the session.

### Workshop Outline

The workshop lasted 90 minutes and followed a timed schedule:

• Optional readings (pre- or postworkshop): approximately 20 minutes but participant dependent.
• Introduction and objectives: 10 minutes.
• Demonstration of interactive teaching tools: 30 minutes.
• Breakout sessions: 40 minutes total.
  ○ Small-group discussion: 15 minutes.
  ○ Facilitated large-group discussion: 25 minutes.
• Session summary and evaluation: 10 minutes.

If needed, the workshop could also be lengthened to 120 minutes to allow for more discussion and/or incorporate the information from the preworkshop reading as didactic content into the session.

### Optional Readings

Prior to the workshop for our MHPE program, we sent participants a PowerPoint file (Appendix A) on the educational theory underpinning virtual teaching and asked them to review the slides and notes on their own; however, for the faculty development workshop conducted at Emory University, this material was provided as a postworkshop reference, since the goal of that workshop focused more on the practical application of virtual teaching. We also sent participants a tech tools worksheet (Appendix B) to facilitate note-taking during the session on relevant aspects of each interactive teaching tool that we discussed. We sent this handout prior to the session, offering participants the option to print it out ahead of time and then fill it in by hand during the workshop.

Prior to the session, the facilitator hosting the Zoom session also did the following:

• Optimized Zoom settings to allow file sharing, breakout rooms, polling, annotations, and screen sharing by all participants.
• Created a Zoom poll asking participants which of the workshop tools they had previously used in their teaching (see Appendix C, slide 9).
• Signed up for a PollEverywhere account and a Padlet account and created the polls and boards that would be needed for the session as outlined in the Facilitator Guide for Tech Demonstrations (Appendix D).

Prior to the session, the workshop assistant(s) did the following tasks:

• Coordinated a strategy to manage questions posed on the Zoom chat.
• Ensured all workshop documents were on hand and ready for dissemination during the session.
• Prepared the postworkshop online evaluation questionnaire and had a shareable link ready for dissemination during the session.

Immediately before the session, the primary facilitator opened a separate internet tab for each tech tool: Backchannel Chat, Padlet, and PollEverywhere. Having these tabs already open allowed the facilitator to easily transition between the various tools during the demonstration. Additionally, the primary facilitator shared the entire desktop screen using Zoom's Share Screen function, not just the PowerPoint, to make it easier to transition between the presentation slides and the various tabs of the internet browser during the session.

### Workshop Introduction

At the beginning of the workshop, we introduced ourselves to the group, reviewed the purpose of the session, and provided an overview of the rationale for the topic using the workshop PowerPoint (Appendix C, slides 1–8). We used the Zoom polling features to determine how familiar participants were with the interactive tools being discussed (Appendix C, slide 9), which allowed us to tailor the demonstration of the tools to the audience. Throughout the session, one of the facilitators monitored the Zoom chat, which was the primary method we used to allow participants to ask questions and provide comments.

### Demonstration of Interactive Teaching Tools

After the introduction, one of the facilitators shared the tech tools worksheet (Appendix B) in the chat in case some participants had not received the presession email. We instructed the participants to use this handout to take notes during the demonstrations. The primary facilitator used a combination of PowerPoint slides (Appendix C, slides 10–45) and interactive demonstrations to introduce each of the following tech tools: Zoom chat, Backchannel Chat, Zoom whiteboard and annotations, Padlet, Zoom polling, PollEverywhere, and Zoom breakout rooms. The facilitator used the script listed in the notes section of the PowerPoint slides; screenshots in the Facilitator Guide for Tech Demonstrations (Appendix D) showed how to perform the interactive demonstrations. During this time, participants interacted with the tools as learners, allowing them to experience the pros and cons of each tool; we emphasized these aspects during the demonstration.

### Breakout Session

We asked participants to work in small groups to develop a plan for a virtual interactive case-based session using the Breakout Session Worksheet (Appendix E), which we shared using the File Share option in the chat. We wanted participants to consider what tools would be useful for different portions of the case (e.g., eliciting history, forming a differential diagnosis, deciding on a final diagnosis, etc.) and how this might differ for a small versus large group of learners. Participants were also asked to reflect on how anonymity and learner level (i.e., student, resident, fellow, or faculty) would impact their choice. We told participants that they could use as many (or as few) tools as they wanted and that they could also select other tools not mentioned in the session. We used Zoom breakout rooms to automatically place participants into groups of four to six participants for 15 minutes. In the first session, participants were divided based on their preassigned MHPE group. In the second session, participants were randomly assigned to breakout rooms. One difficulty we faced was the inability to easily circulate among groups to answer questions without disrupting their discussions. To overcome this issue, we told participants that they could rejoin the main room anytime they had questions for the facilitators. In addition, we joined each group when 5 minutes were left to see if any further time or help was needed.

After the small-group activity, we reconvened as a large group. We asked each group to spend a few minutes describing its choice of tools and rationale for selecting those tools. Participants were encouraged to use the chat to provide additional comments. After each group's presentation, we used the following questions and optional prompts to encourage discussion:

• Why did you select a particular tool over another?
• What factors influenced this decision (e.g., anonymity, familiarity, ease of use, class size, learner level)?
• Can you propose a different approach to other groups' educational design?
• What are the advantages and disadvantages of the tools that you selected?
• Aside from the virtual tools presented today, would you use any other virtual tools to enhance your educational intervention?

One facilitator moderated the group discussion while another monitored the chat, periodically bringing forward the group's additional comments and questions as a stimulus for further discussion. We did not formally record each group's and participants' suggestions during our session, but it could be helpful to have one of the facilitators share a screen and type each group's suggestions on one central document, which could be used as both a workshop summary and a postworkshop handout.

### Workshop Summary

We ended the workshop with a brief summary of lessons learned from the discussion and reviewed some best practices for effective videoconferencing (Appendix C, slides 50–53).

### Evaluation Strategy

At the end of the workshop, we shared a link to our online evaluation (Appendix F). This form was designed by the workshop facilitators and used a 5-point Likert scale (1 = *strongly disagree,* 5 = *strongly agree*) to assess participants' responses. We asked participants to evaluate the session (learning objectives, organization, facilitator knowledge, and reference materials) and their perceived learning outcomes (i.e., familiarity with tools discussed, comfort in using tools, and plans for using them in the future). We also collected open-ended responses regarding knowledge gained and opportunities for improvement.

## Results

We presented the first iteration of the workshop (Workshop 1) for our final seminar in our MHPE Instruction and Assessment course in July 2020. All of the health professions education graduate students enrolled in the course responded to the evaluation (18 participants, response rate: 100%). The second iteration of the workshop (Workshop 2) was given as a faculty development seminar at the Emory University School of Medicine to a group of faculty small-group facilitators in August 2020. Participation rate was high at 86% (26 out of 30). Survey response rate was moderate at 65% (17 out of 26).

Both groups provided high ratings for our workshop, as shown in the Table. Overall, the workshop was clear, organized, and relevant. The workshop increased participants' familiarity and comfort with using various interactive tools for online teaching. On average, participants in Workshop 1 reported slightly lower ratings for the use of seminal articles and appropriate reference materials compared to participants in Workshop 2.

**Table.**
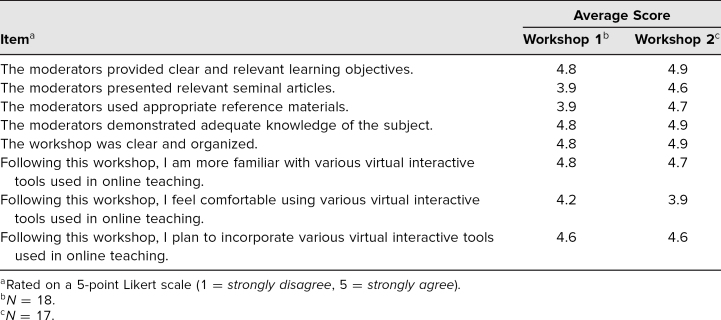
Evaluation Scores by Workshop Session

Narrative comments were collected in both versions of the workshop and differed slightly between workshops. In both workshops, participants described an increased level of familiarity with and awareness of various technological and interactive tools used to augment teaching virtually. However, wanting additional time to practice with the presented tools was a common critique.

• “I think the most important was seeing the variety of options available.” (Participant, Workshop 1)
• When asked for take-home messages: “1. Recognize the pitfalls of using only [one] modality for teaching; 2. Using other resources such as chat and polling to enhance learning; 3. Plan ahead with teaching materials.” (Participant, Workshop 2)

A few participants in Workshop 2 also discussed the importance of planning ahead of time how a teaching session would be facilitated using some of the interactive tools presented. Some also learned how to use these tools to engage different learners during virtual teaching sessions.

• When asked for take-home messages: “How to make everyone in the group more involved… to keep learners engaged rather than [the teacher] just talking….” (Participant, Workshop 2)

Participants in Workshop 1 requested additional data comparing the effectiveness of virtual teaching with traditional in-person methods. Similarly, the second participant group wondered about published outcomes related to the use of the presented tools in virtual teaching. Both groups requested additional information on how to access and set up accounts for these tools, including additional information on pricing, security, and privacy.

• When asked for ways to improve the workshop: “One of the difficulties is that some of the services require membership to get full access… maybe distinguishing between free software and paid software.” (Participant, Workshop 1)
• “This session was great for seeing and using the different platforms as learners, but it would be helpful to have a follow-up email with a list of steps on how to create the faculty… account… because it's hard to remember the different details of each program.” (Participant, Workshop 2)

## Discussion

At a time when distance online learning is garnering increased interest and accessibility to online teaching tools is flourishing, the topic of how to effectively integrate some of these modalities to enhance interactive online education is of particular interest to health professions educators. Although online teaching tools may provide increased flexibility in the content being taught and the process of teaching, maintaining learner engagement, especially with case-based sessions, is key to ensuring that learning outcomes are achieved. Many tools exist to increase interactivity during online teaching; however, educators need to be familiar with the advantages and disadvantages of each to determine the best choice for achieving their intended learning outcomes. The literature on faculty development initiatives aimed at improving health professions educators' knowledge of and skills with a variety of online teaching tools is lacking. Our workshop aimed to address this gap in the hope of enhancing learners' virtual educational experience.

Participants found this workshop valuable because it allowed them to engage with each interactive tool as a learner, thereby experiencing the advantages and disadvantages of these tools firsthand. This workshop provided practical exposure to teaching tools and encouraged participants to consider how they could implement them for case-based teaching sessions in their own educational setting. The breakout sessions and the large-group discussions were an important component that helped many participants discuss their unanswered questions about each tool during the workshop. Although we were initially worried that participants would all develop the same teaching plan in the breakout sessions, we found that each group developed a unique approach and provided different perspectives on the optimal tools for each portion of the case. The large-group discussion after the breakout session allowed participants to share their personal experiences with online teaching, and we found that participants learned from each other's stories.

After the first workshop, we realized that most participants were familiar with basic functions in our videoconference platforms (i.e., chat, polling, and whiteboard) and that they preferred to spend more time learning about alternative tools to incorporate into their sessions. Thus, for the second workshop, more time was spent reviewing the alternative technology tools. This change also allowed more time for participants to ask questions while introducing these tools. For the second iteration of the workshop, only one facilitator was available to lead the session, which made it more difficult to monitor participant comments in the chat. To adjust for this, the facilitator asked participants to unmute themselves to ask questions or to enter queries into the chat to be addressed at the end of the session. For future iterations of this workshop, we recommend that facilitators determine participants' baseline familiarity with the various tools (e.g., via a poll at the beginning of the session) and adjust the time spent on each tool accordingly.

We identified several limitations of our workshop after piloting it with two groups of participants. First, the facilitators need to have familiarity with the interactive teaching tools in order to effectively run the session. However, most of the tools are user-friendly, can be easily learned by watching readily available online tutorials, and do not require unreasonable time commitment from the facilitators to familiarize themselves beforehand. Second, participants need to have at least some familiarity with online teaching and videoconferencing platforms. Learning multiple new tools at once can be overwhelming, but we found that most participants picked up something valuable from the session and were motivated to practice on their own. Third, technology changes rapidly, which means that some of these teaching tools may change or not be available in the future. To mitigate this issue, we provide an overview of different broad categories of teaching tools, mention a few teaching tools within each category, and demonstrate only established tools already in use in the educational community. We focus on general features of the tools (e.g., anonymity, collaborative potential, etc.), which should allow future facilitators to replace the ones we used with other options. By preferentially selecting the free version of each available tool, we intend the workshop to be generalizable to a broader audience. Lastly, limitations in our small audience sample size and in the self-reporting nature of our workshop evaluation affected our ability to evaluate the effectiveness of the workshop in a robust manner; however, judging by their reactions, participants were enthusiastic about the value of the material.

Potential future directions include presenting this workshop to national and international audiences. At the local and national levels, it can be integrated into faculty development initiatives related to teaching and learning in health professions education. At the international level, adapting the content and activities of the workshop into a massive open online course might be a challenge worth pursuing, as fostering effective interaction among such a large audience would be innovative.

## Appendices

Optional Readings.pptxInteractive Tools Worksheet.docxWorkshop Presentation.pptxFacilitator Guide Tech Demo.docxBreakout Session Worksheet.docxWorkshop Evaluation.docx
All appendices are peer reviewed as integral parts of the Original Publication.
