# Supplementary material for: A Faculty Development Workshop for Planning and Implementing Interactive Virtual Case-Based Teaching
Source: MedEdPORTAL. 2021 Mar 17;17:11126. doi: 10.15766/mep_2374-8265.11126 (PMC7970636; doi:10.15766/mep_2374-8265.11126)
Supplement: Supplementary file 1 — Optional Readings.pptxInteractive Tools Worksheet.docxWorkshop Presentation.pptxFacilitator Guide Tech Demo.docxBreakout Session Worksheet.docxWorkshop Evaluation.docx [file mep_2374-8265.11126-s001.zip › A. Optional Readings.pptx]

## Slide 1
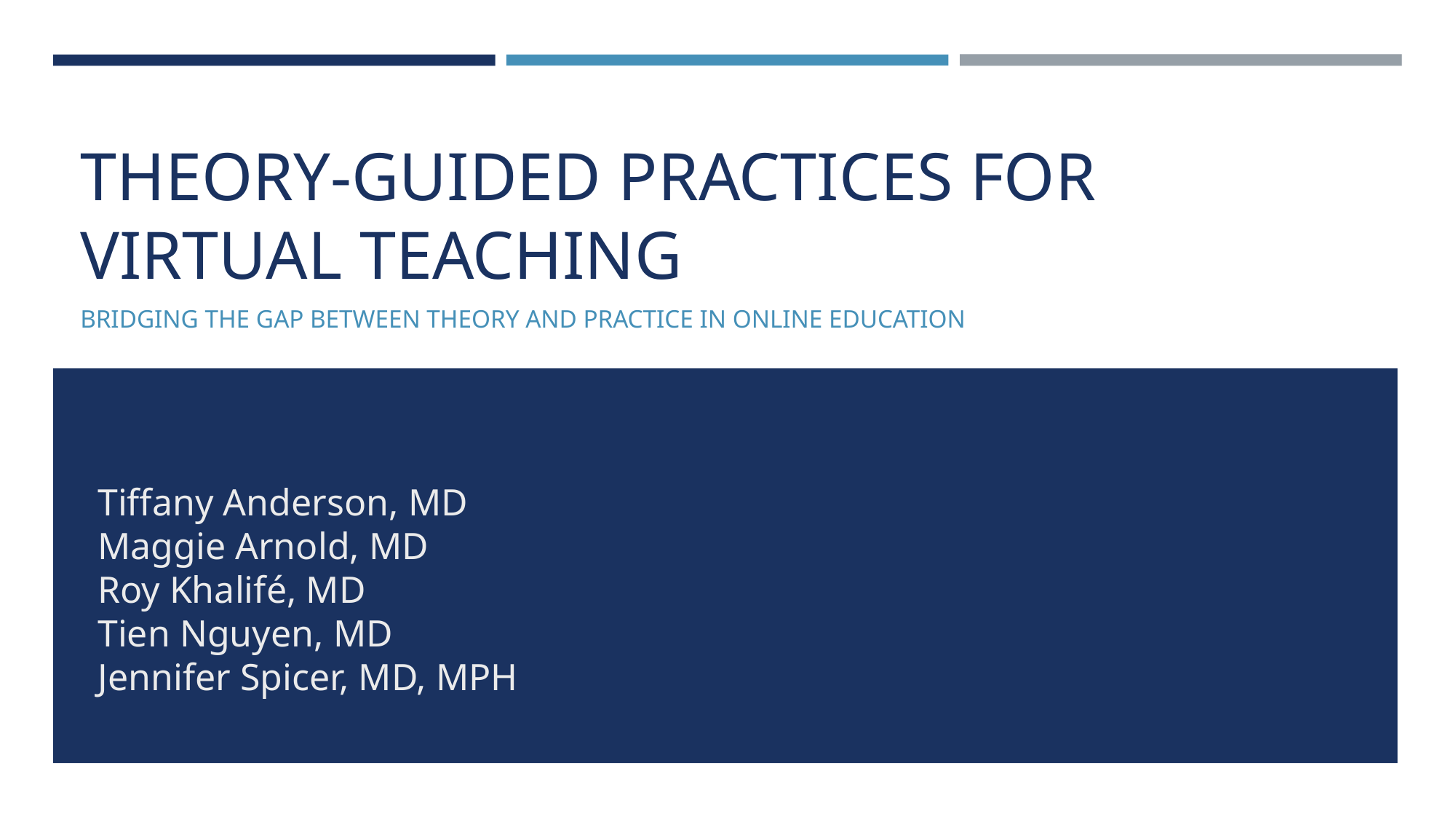

# THEORY-GUIDED PRACTICES FOR VIRTUAL TEACHING
BRIDGING THE GAP BETWEEN THEORY AND PRACTICE IN ONLINE EDUCATION
Tiffany Anderson, MD
Maggie Arnold, MD
Roy Khalifé, MD
Tien Nguyen, MD
Jennifer Spicer, MD, MPH

## Slide 2
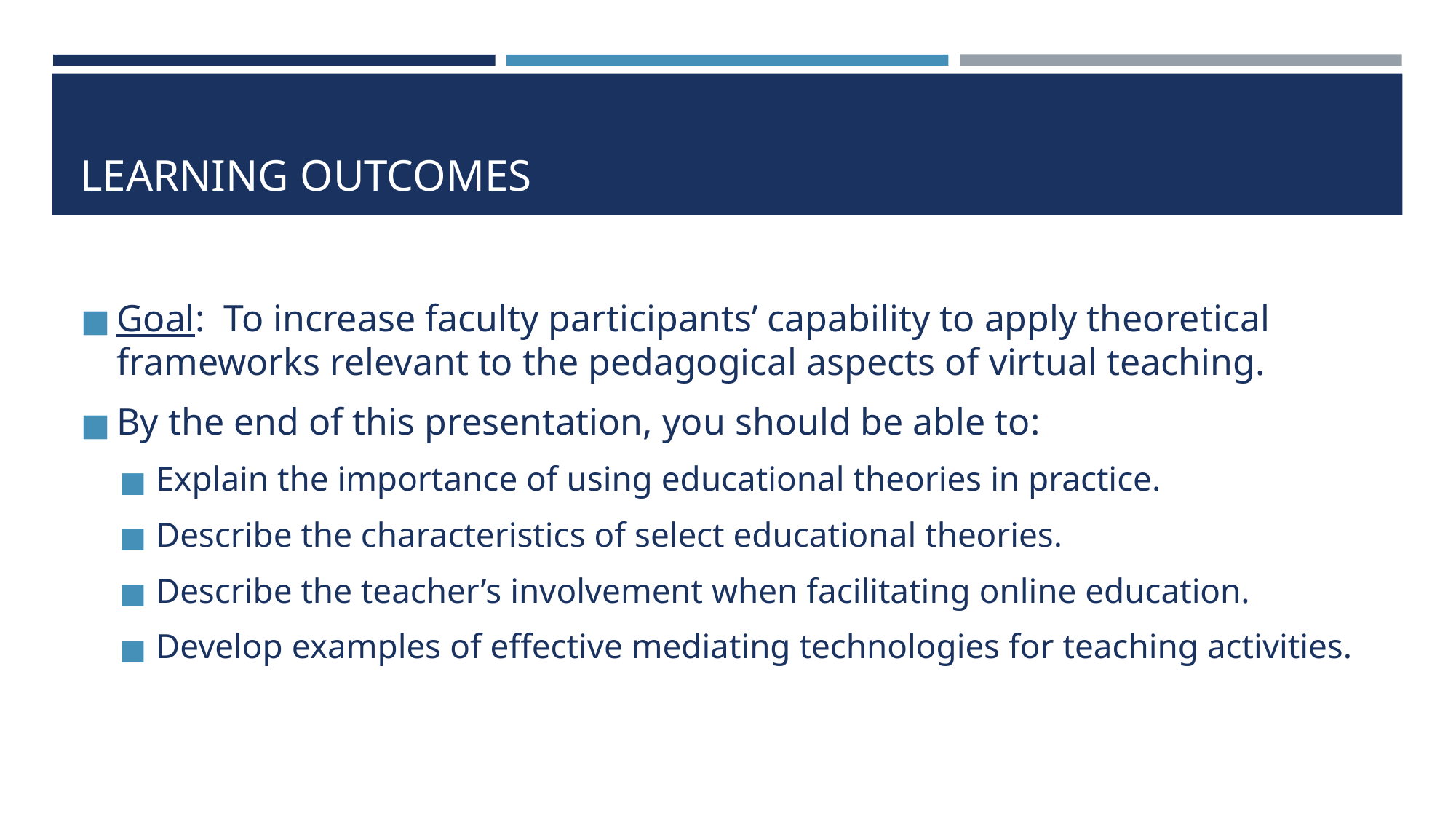

# LEARNING OUTCOMES
Goal: To increase faculty participants’ capability to apply theoretical frameworks relevant to the pedagogical aspects of virtual teaching.
By the end of this presentation, you should be able to:
Explain the importance of using educational theories in practice.
Describe the characteristics of select educational theories.
Describe the teacher’s involvement when facilitating online education.
Develop examples of effective mediating technologies for teaching activities.

## Slide 3
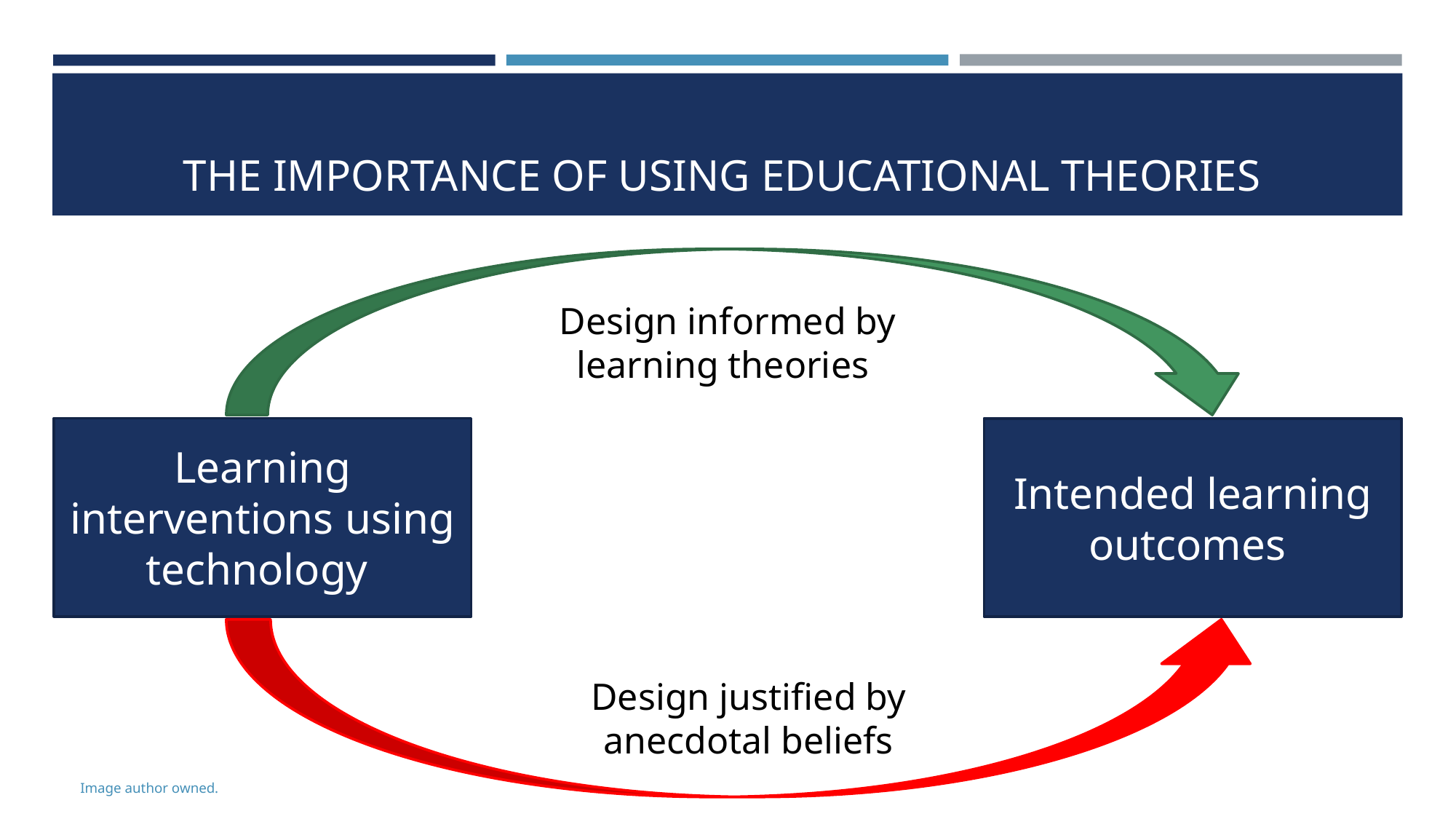

# THE IMPORTANCE OF USING EDUCATIONAL THEORIES
Design informed by learning theories
Learning interventions using technology
Intended learning outcomes
Design justified by anecdotal beliefs
Image author owned.

## Slide 4
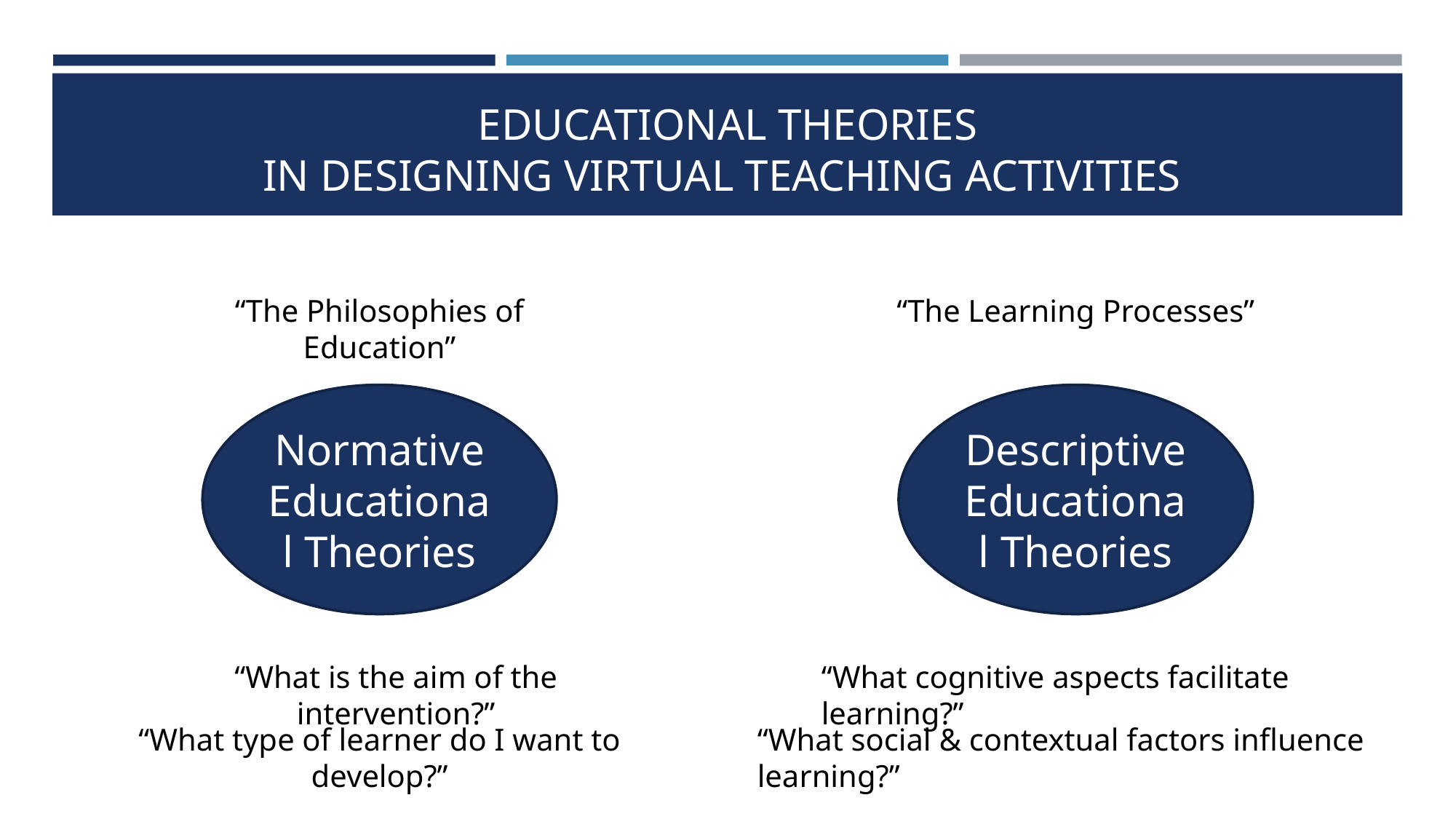

# EDUCATIONAL THEORIES IN DESIGNING VIRTUAL TEACHING ACTIVITIES
“The Philosophies of Education”
“The Learning Processes”
Normative Educational Theories
Descriptive Educational Theories
“What is the aim of the intervention?”
“What cognitive aspects facilitate learning?”
“What type of learner do I want to develop?”
“What social & contextual factors influence learning?”

## Slide 5
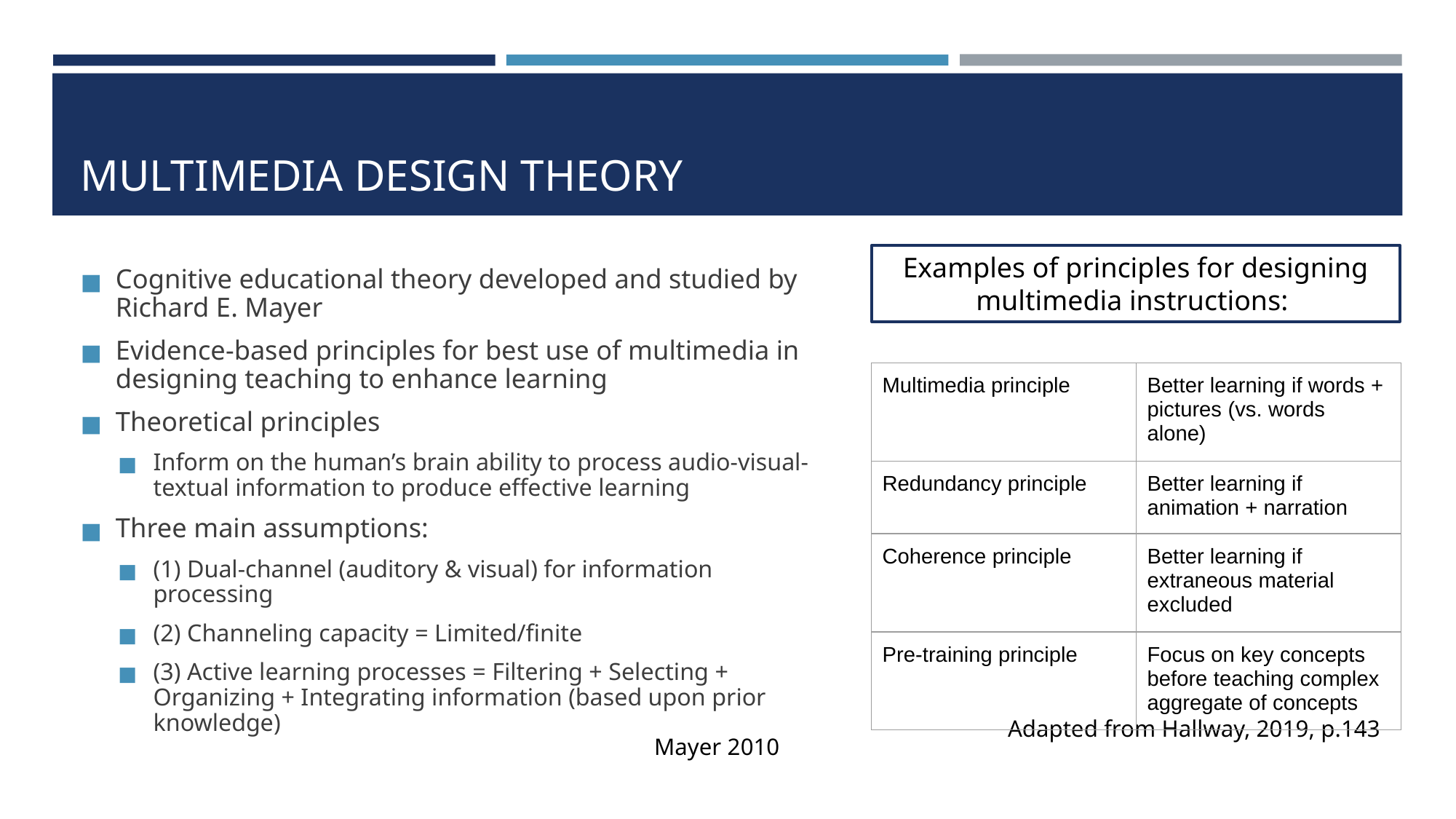

# MULTIMEDIA DESIGN THEORY
Examples of principles for designing multimedia instructions:
Cognitive educational theory developed and studied by Richard E. Mayer
Evidence-based principles for best use of multimedia in designing teaching to enhance learning
Theoretical principles
Inform on the human’s brain ability to process audio-visual-textual information to produce effective learning
Three main assumptions:
(1) Dual-channel (auditory & visual) for information processing
(2) Channeling capacity = Limited/finite
(3) Active learning processes = Filtering + Selecting + Organizing + Integrating information (based upon prior knowledge)
| Multimedia principle | Better learning if words + pictures (vs. words alone) |
| --- | --- |
| Redundancy principle | Better learning if animation + narration |
| Coherence principle | Better learning if extraneous material excluded |
| Pre-training principle | Focus on key concepts before teaching complex aggregate of concepts |
Adapted from Hallway, 2019, p.143
Mayer 2010

## Slide 6
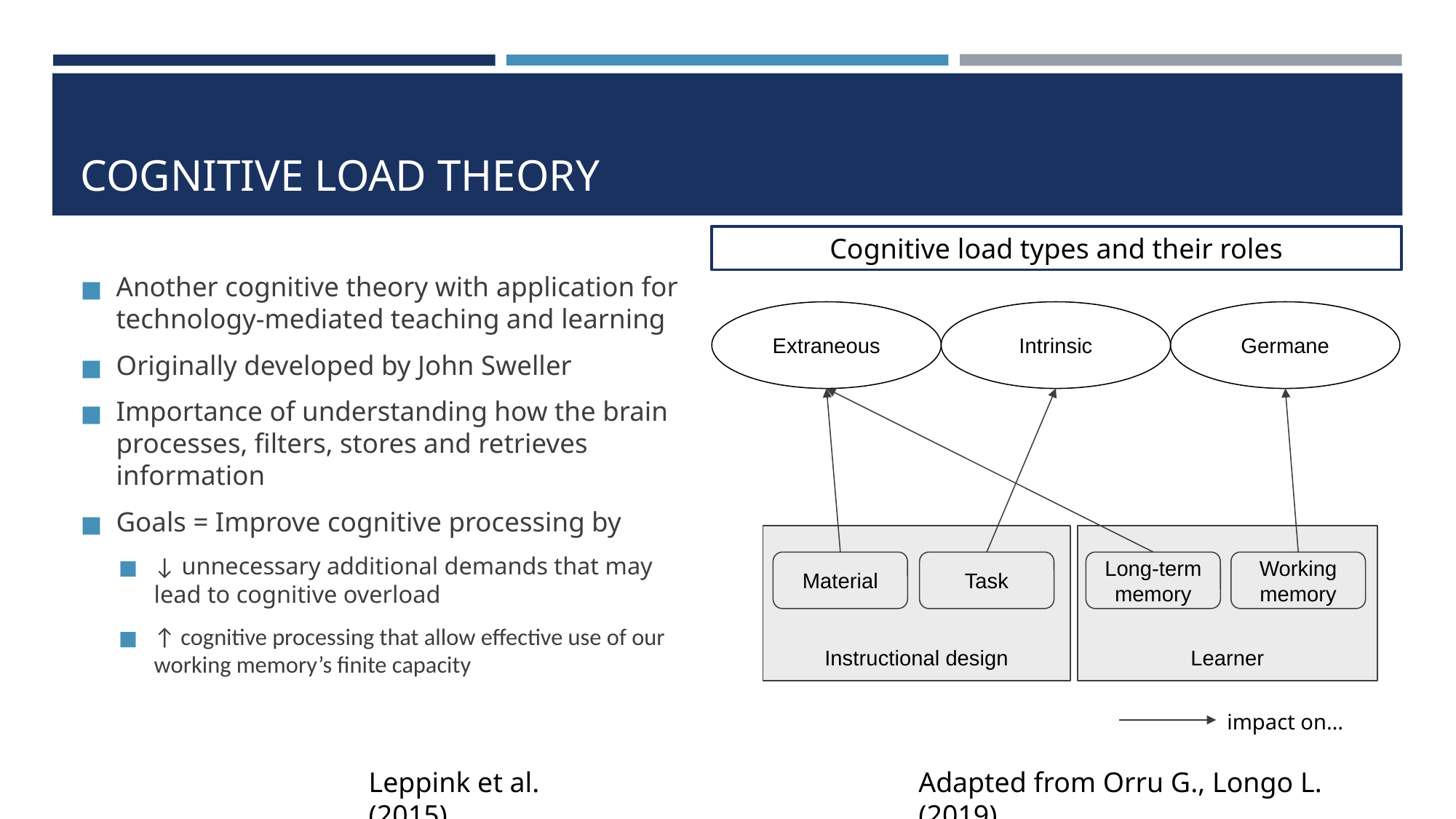

# COGNITIVE LOAD THEORY
Cognitive load types and their roles
Another cognitive theory with application for technology-mediated teaching and learning
Originally developed by John Sweller
Importance of understanding how the brain processes, filters, stores and retrieves information
Goals = Improve cognitive processing by
↓ unnecessary additional demands that may lead to cognitive overload
↑ cognitive processing that allow effective use of our working memory’s finite capacity
Extraneous
Intrinsic
Germane
Instructional design
Learner
Material
Task
Working memory
Long-term memory
impact on…
Leppink et al. (2015)
Adapted from Orru G., Longo L. (2019)

## Slide 7
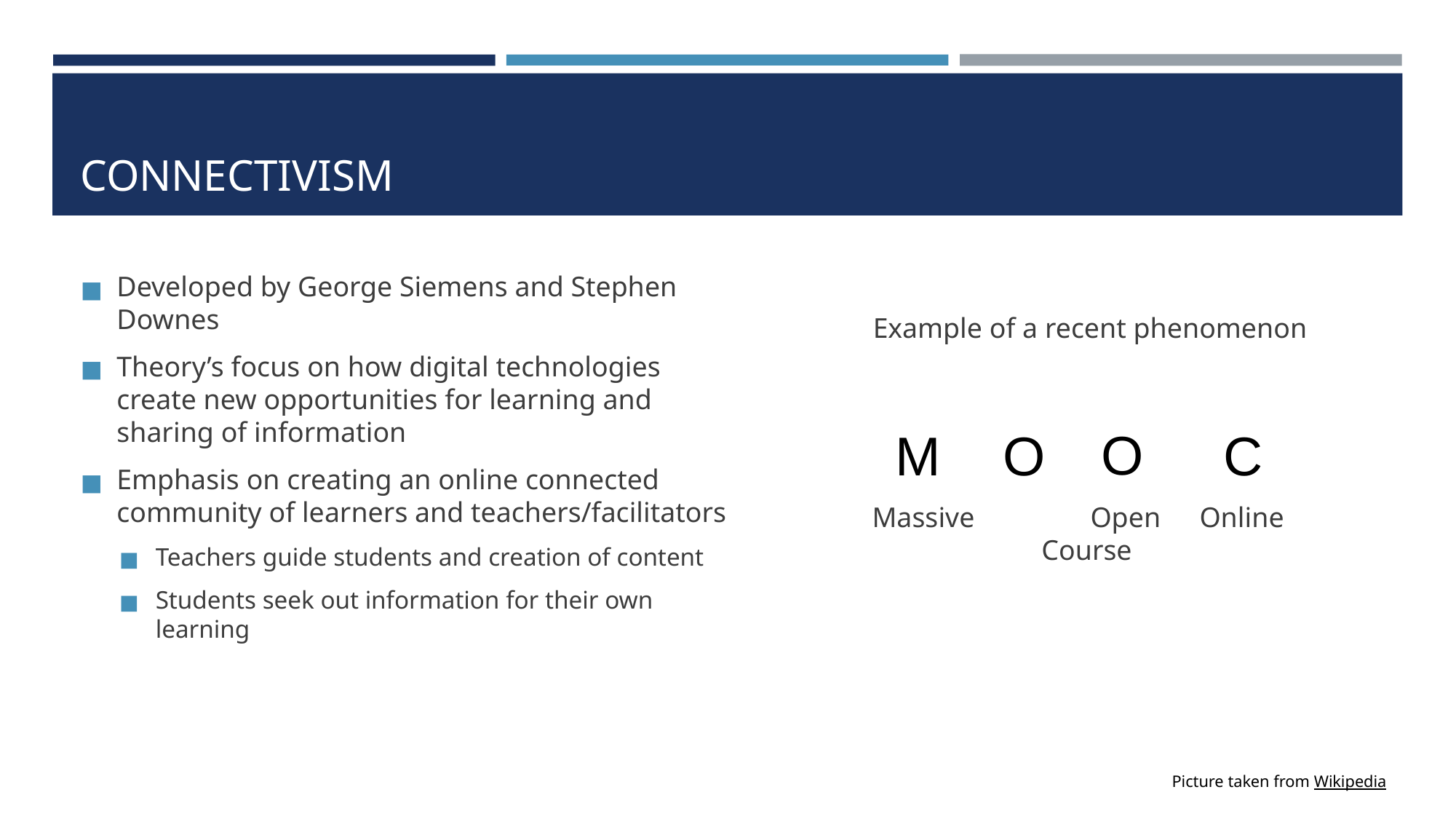

# CONNECTIVISM
Developed by George Siemens and Stephen Downes
Theory’s focus on how digital technologies create new opportunities for learning and sharing of information
Emphasis on creating an online connected community of learners and teachers/facilitators
Teachers guide students and creation of content
Students seek out information for their own learning
Example of a recent phenomenon
Massive 	Open 	Online 	Course
O
O
C
M
Picture taken from Wikipedia

## Slide 8
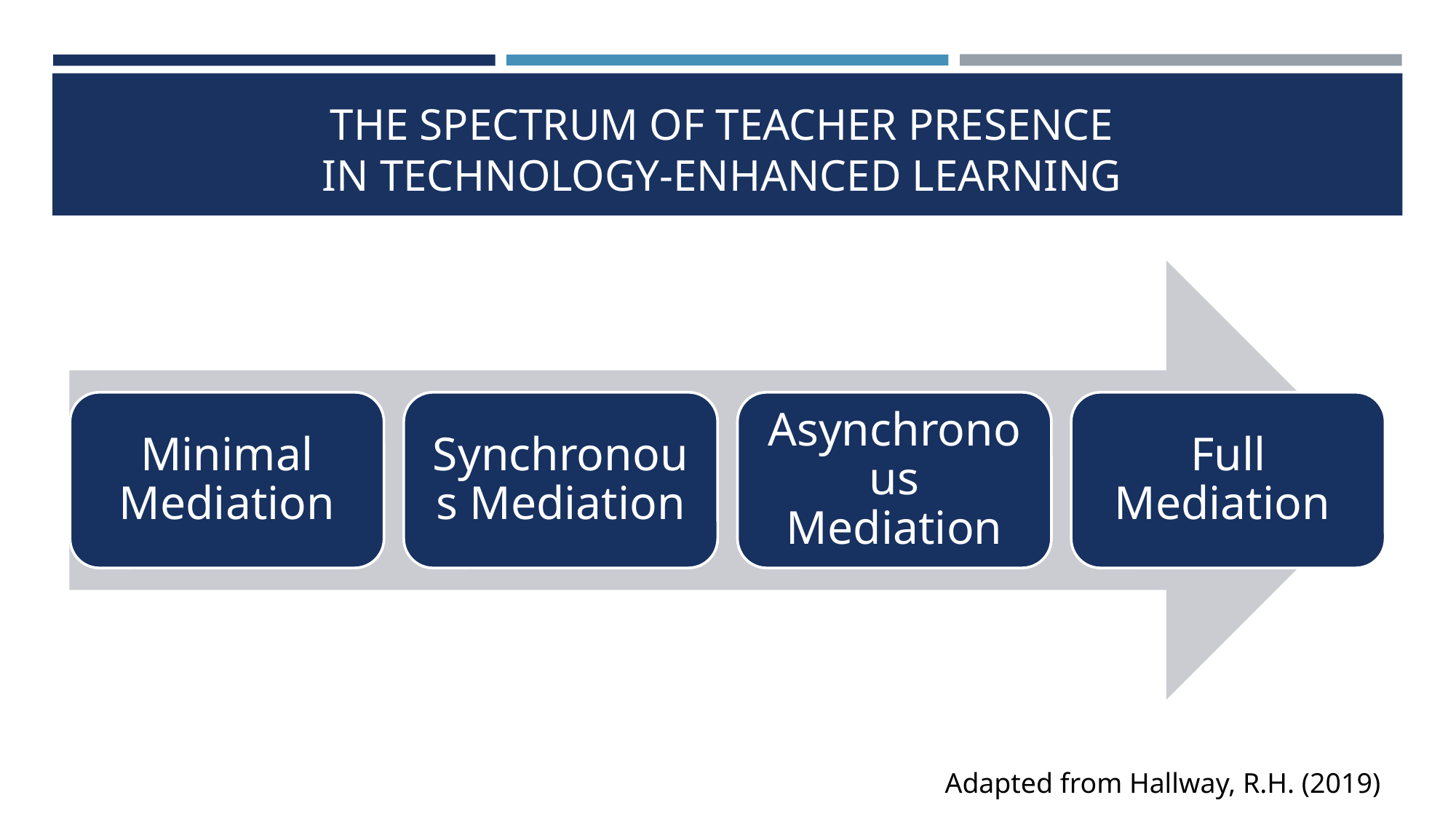

# THE SPECTRUM OF TEACHER PRESENCE IN TECHNOLOGY-ENHANCED LEARNING
Minimal Mediation
Synchronous Mediation
Asynchronous Mediation
Full Mediation
Adapted from Hallway, R.H. (2019)

## Slide 9
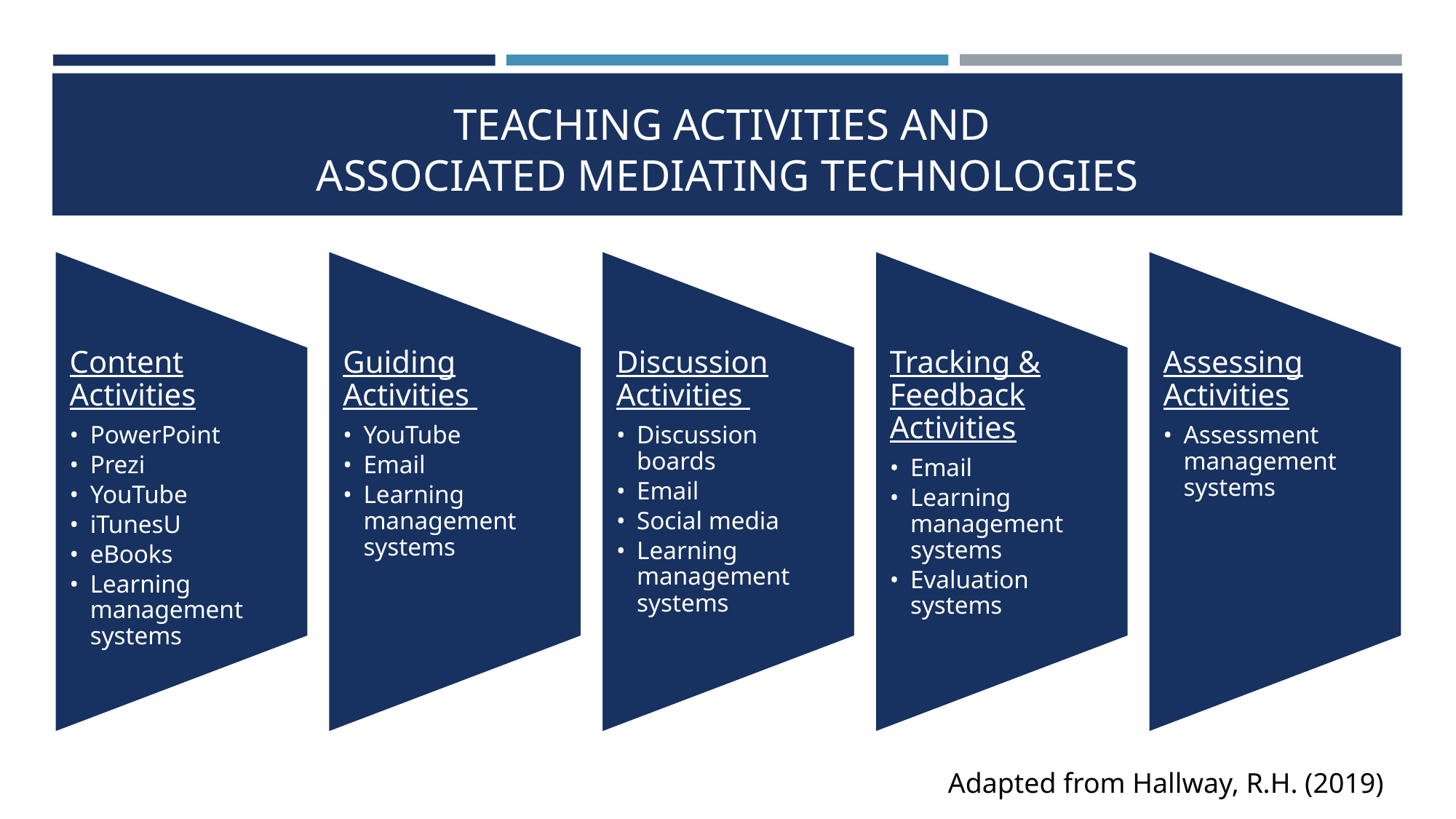

# TEACHING ACTIVITIES AND ASSOCIATED MEDIATING TECHNOLOGIES
Content Activities
PowerPoint
Prezi
YouTube
iTunesU
eBooks
Learning management systems
Guiding Activities
YouTube
Email
Learning management systems
Discussion Activities
Discussion boards
Email
Social media
Learning management systems
Tracking & Feedback Activities
Email
Learning management systems
Evaluation systems
Assessing Activities
Assessment management systems
Adapted from Hallway, R.H. (2019)

## Slide 10
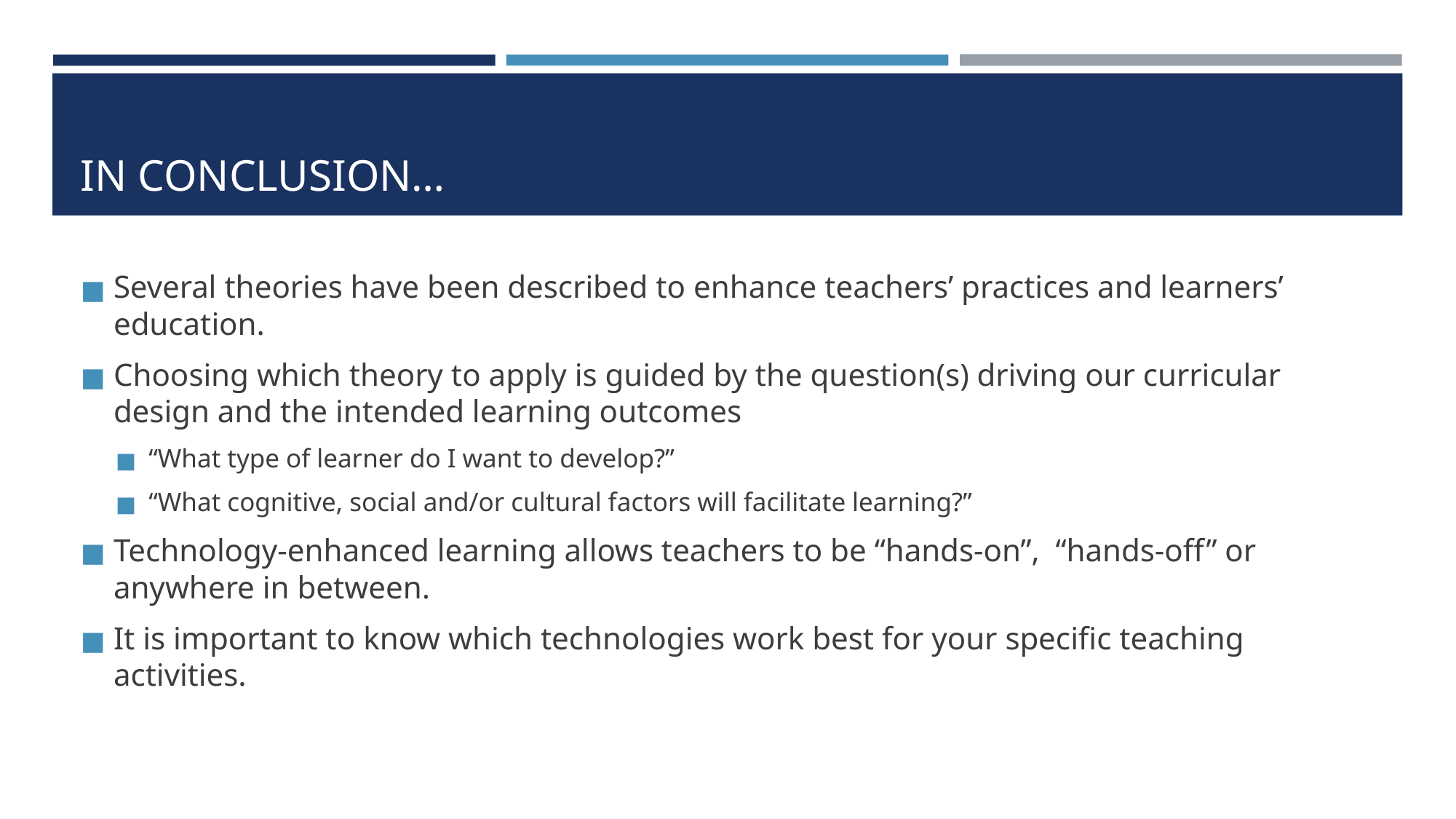

# IN CONCLUSION…
Several theories have been described to enhance teachers’ practices and learners’ education.
Choosing which theory to apply is guided by the question(s) driving our curricular design and the intended learning outcomes
“What type of learner do I want to develop?”
“What cognitive, social and/or cultural factors will facilitate learning?”
Technology-enhanced learning allows teachers to be “hands-on”, “hands-off” or anywhere in between.
It is important to know which technologies work best for your specific teaching activities.

## Slide 11
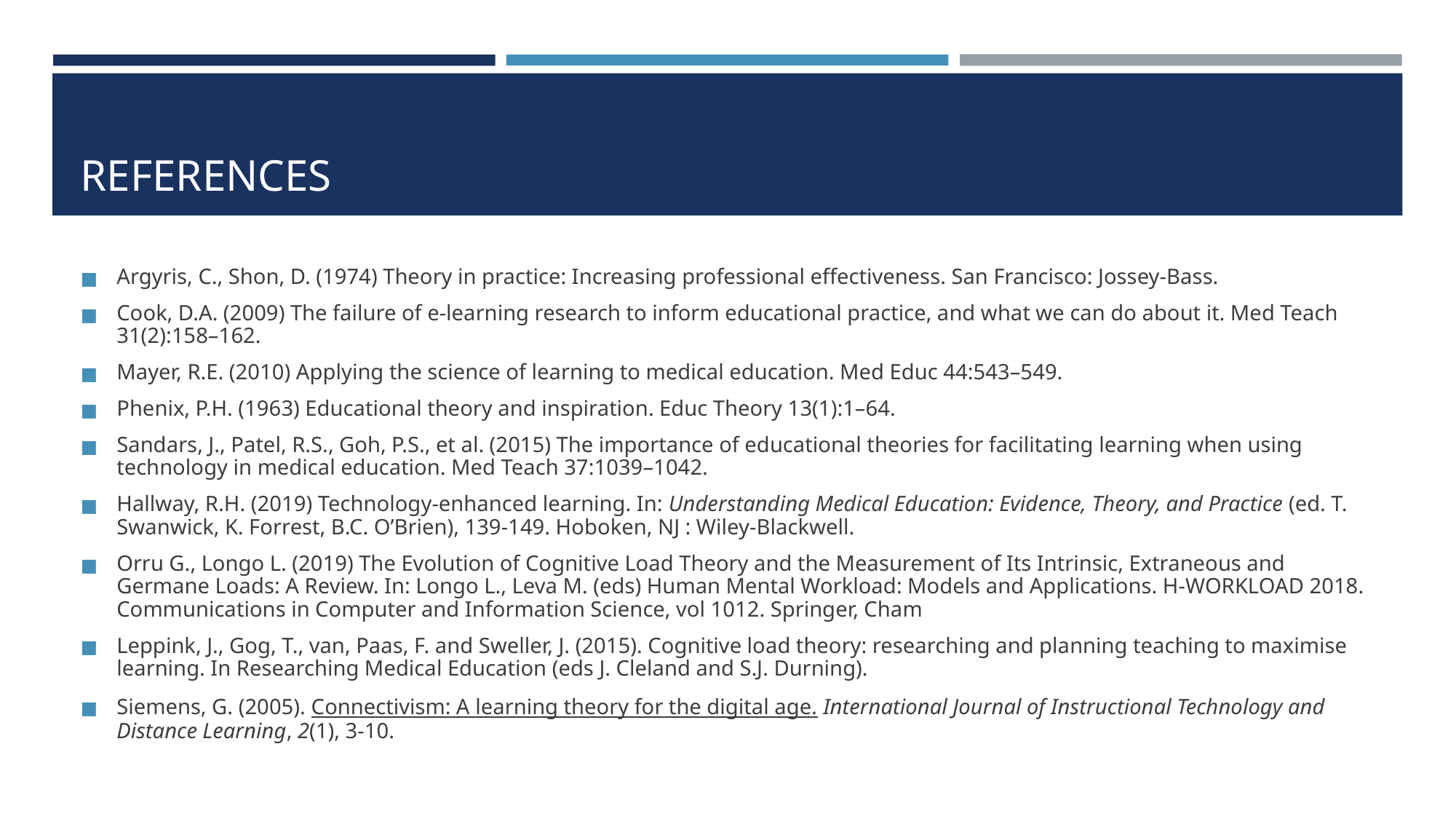

# REFERENCES
Argyris, C., Shon, D. (1974) Theory in practice: Increasing professional effectiveness. San Francisco: Jossey-Bass.
Cook, D.A. (2009) The failure of e-learning research to inform educational practice, and what we can do about it. Med Teach 31(2):158–162.
Mayer, R.E. (2010) Applying the science of learning to medical education. Med Educ 44:543–549.
Phenix, P.H. (1963) Educational theory and inspiration. Educ Theory 13(1):1–64.
Sandars, J., Patel, R.S., Goh, P.S., et al. (2015) The importance of educational theories for facilitating learning when using technology in medical education. Med Teach 37:1039–1042.
Hallway, R.H. (2019) Technology-enhanced learning. In: Understanding Medical Education: Evidence, Theory, and Practice (ed. T. Swanwick, K. Forrest, B.C. O’Brien), 139-149. Hoboken, NJ : Wiley-Blackwell.
Orru G., Longo L. (2019) The Evolution of Cognitive Load Theory and the Measurement of Its Intrinsic, Extraneous and Germane Loads: A Review. In: Longo L., Leva M. (eds) Human Mental Workload: Models and Applications. H-WORKLOAD 2018. Communications in Computer and Information Science, vol 1012. Springer, Cham
Leppink, J., Gog, T., van, Paas, F. and Sweller, J. (2015). Cognitive load theory: researching and planning teaching to maximise learning. In Researching Medical Education (eds J. Cleland and S.J. Durning).
Siemens, G. (2005). Connectivism: A learning theory for the digital age. International Journal of Instructional Technology and Distance Learning, 2(1), 3-10.
