# Supplementary material for: A Faculty Development Workshop for Planning and Implementing Interactive Virtual Case-Based Teaching
Source: MedEdPORTAL. 2021 Mar 17;17:11126. doi: 10.15766/mep_2374-8265.11126 (PMC7970636; doi:10.15766/mep_2374-8265.11126)
Supplement: Supplementary file 1 — Optional Readings.pptxInteractive Tools Worksheet.docxWorkshop Presentation.pptxFacilitator Guide Tech Demo.docxBreakout Session Worksheet.docxWorkshop Evaluation.docx [file mep_2374-8265.11126-s001.zip › B. Interactive Tools Worksheet.docx]

Appendix B: Interactive Tools for Virtual Teaching Worksheet

| **Tool** | **# Participants** (free account) | **Participant Anonymity?** | **Pros** | **Cons** | **Potential Uses**  (brainstorming/discussing/deciding) |
| --- | --- | --- | --- | --- | --- |
| Zoom Chat |  |  |  |  |  |
| Backchannel Chat |  |  |  |  |  |
| Zoom whiteboard with annotations |  |  |  |  |  |
| Padlet |  |  |  |  |  |
| Zoom Poll |  |  |  |  |  |
| PollEverywhere |  |  |  |  |  |
