# Supplementary material for: A Faculty Development Workshop for Planning and Implementing Interactive Virtual Case-Based Teaching
Source: MedEdPORTAL. 2021 Mar 17;17:11126. doi: 10.15766/mep_2374-8265.11126 (PMC7970636; doi:10.15766/mep_2374-8265.11126)
Supplement: Supplementary file 1 — Optional Readings.pptxInteractive Tools Worksheet.docxWorkshop Presentation.pptxFacilitator Guide Tech Demo.docxBreakout Session Worksheet.docxWorkshop Evaluation.docx [file mep_2374-8265.11126-s001.zip › C. Workshop Presentation.pptx]

## Slide 1
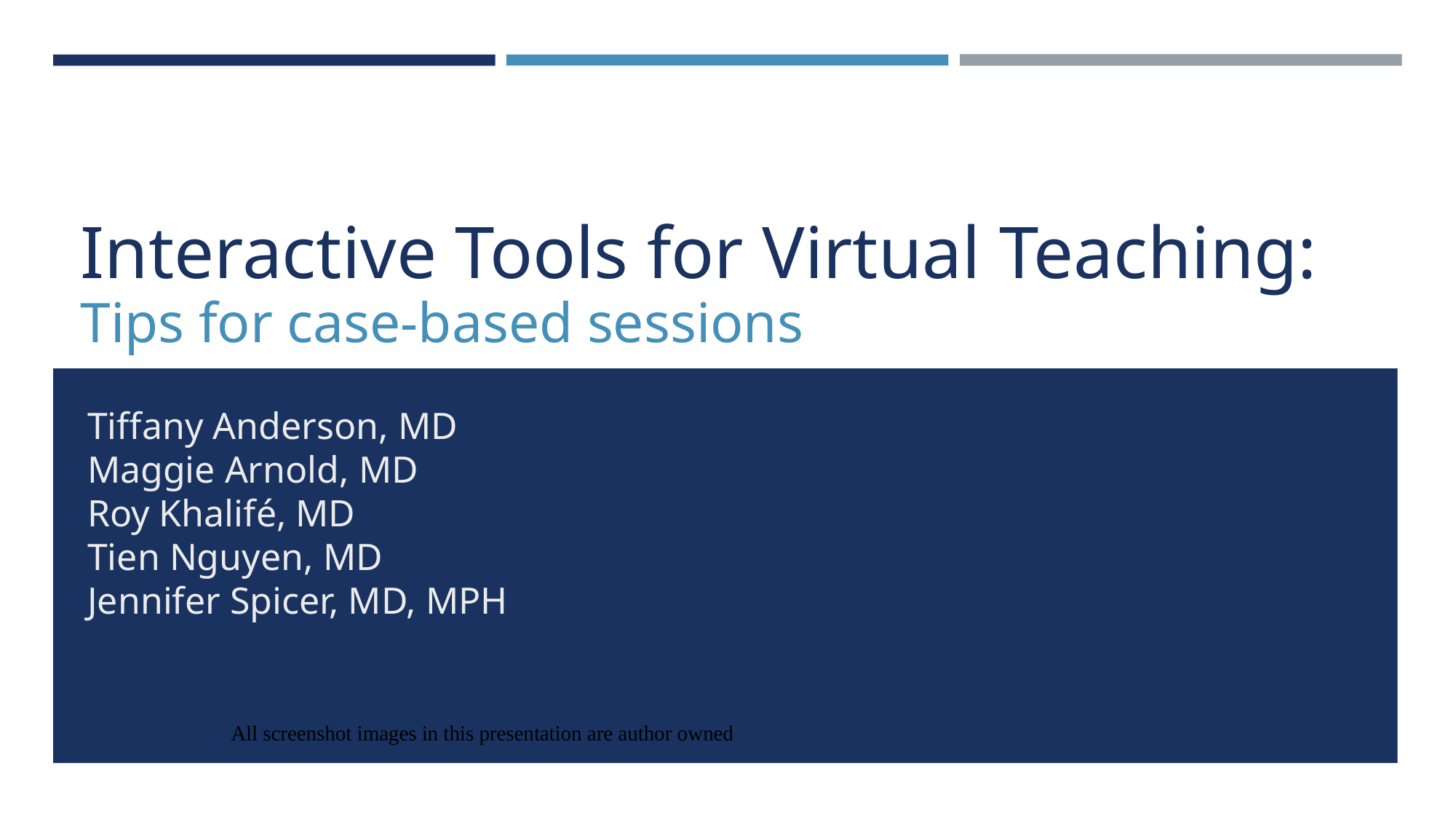

# Interactive Tools for Virtual Teaching:
Tips for case-based sessions
Tiffany Anderson, MD
Maggie Arnold, MD
Roy Khalifé, MD
Tien Nguyen, MD
Jennifer Spicer, MD, MPH
All screenshot images in this presentation are author owned

## Slide 2
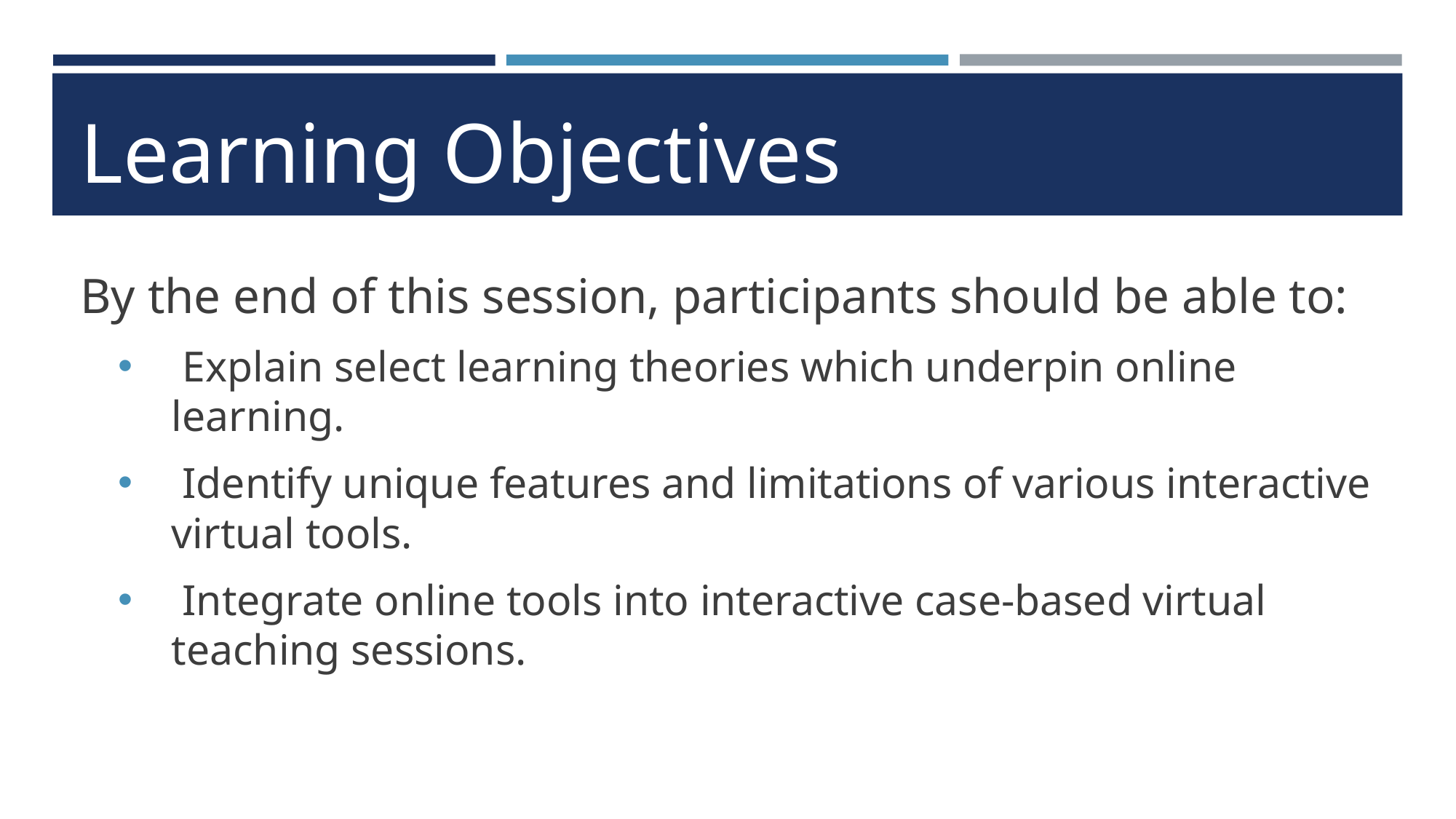

# Learning Objectives
By the end of this session, participants should be able to:
 Explain select learning theories which underpin online learning.
 Identify unique features and limitations of various interactive virtual tools.
 Integrate online tools into interactive case-based virtual teaching sessions.

## Slide 3
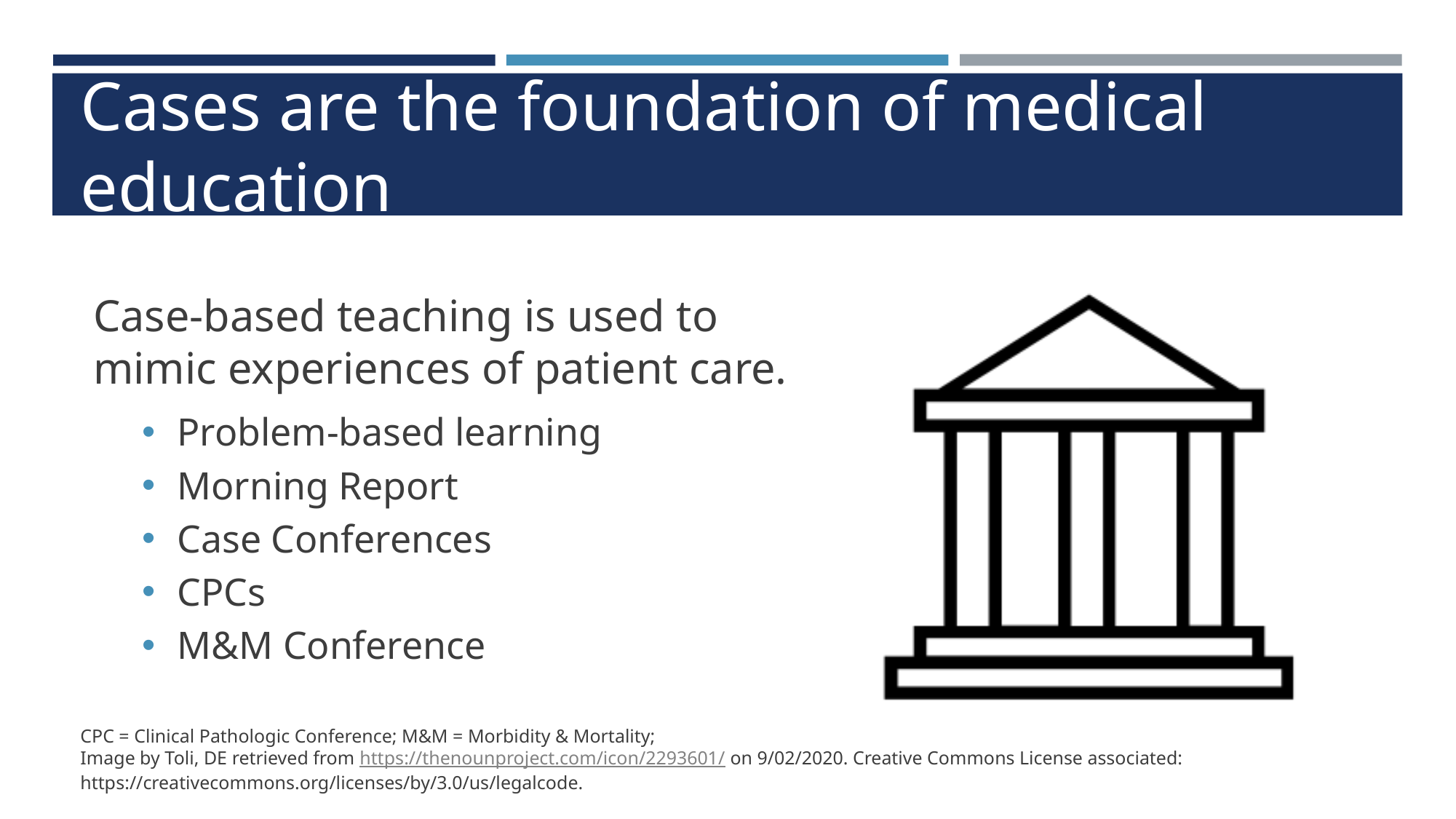

# Cases are the foundation of medical education
Case-based teaching is used to mimic experiences of patient care.
Problem-based learning
Morning Report
Case Conferences
CPCs
M&M Conference
CPC = Clinical Pathologic Conference; M&M = Morbidity & Mortality;
Image by Toli, DE retrieved from https://thenounproject.com/icon/2293601/ on 9/02/2020. Creative Commons License associated: https://creativecommons.org/licenses/by/3.0/us/legalcode.

## Slide 4
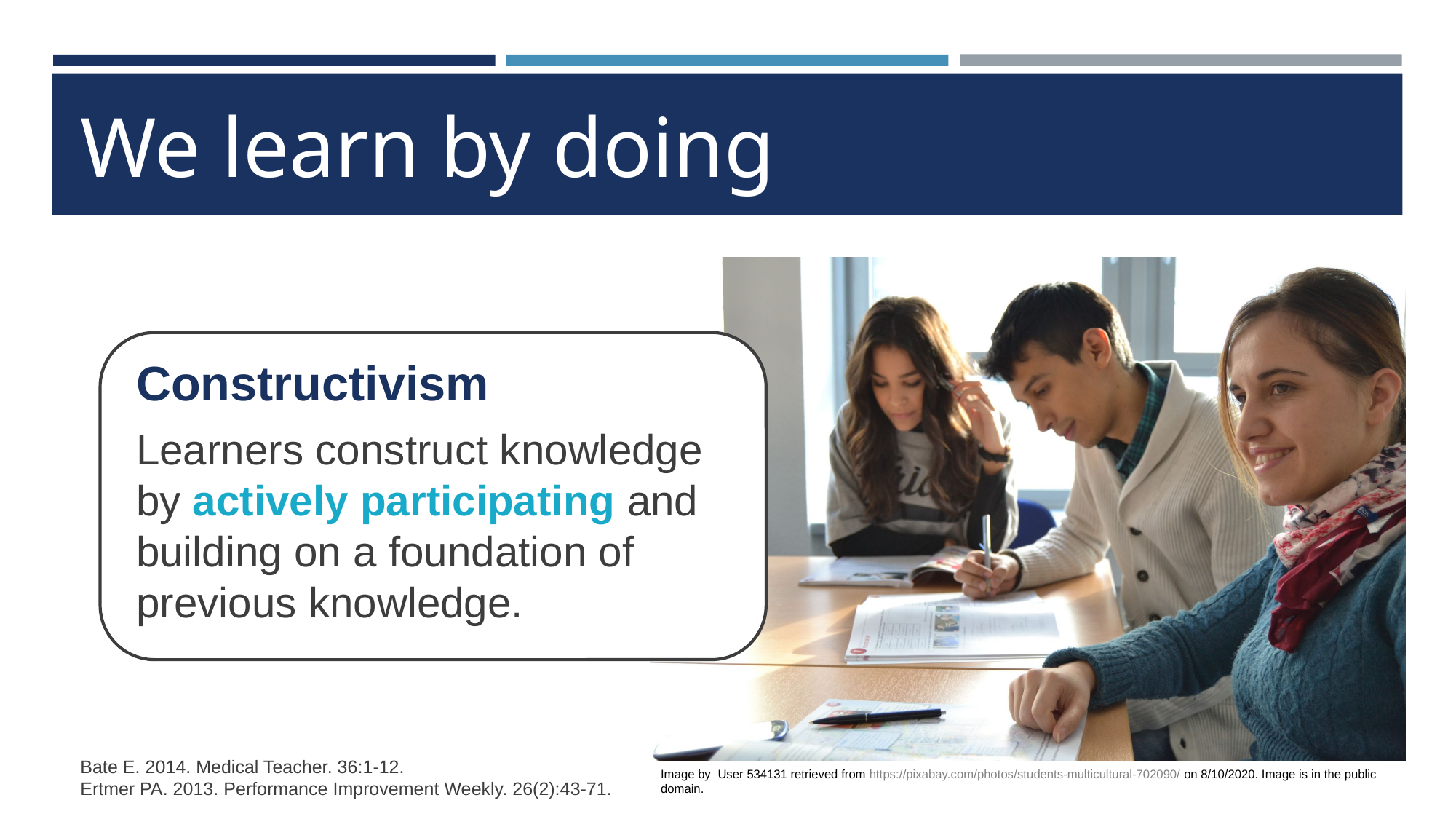

# We learn by doing
Constructivism
Learners construct knowledge by actively participating and building on a foundation of previous knowledge.
Bate E. 2014. Medical Teacher. 36:1-12.
Ertmer PA. 2013. Performance Improvement Weekly. 26(2):43-71.
Image by User 534131 retrieved from https://pixabay.com/photos/students-multicultural-702090/ on 8/10/2020. Image is in the public domain.

## Slide 5
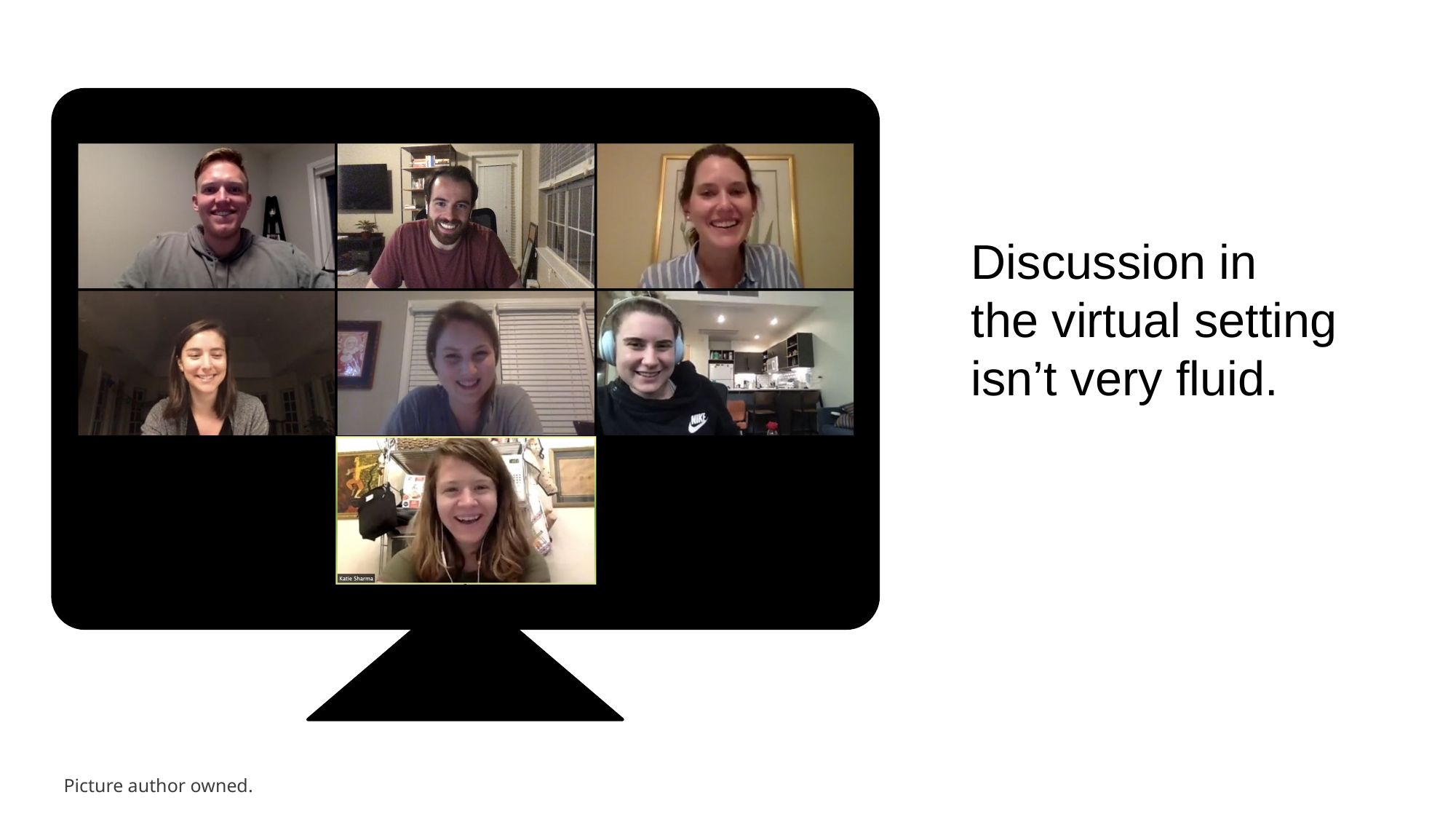

Discussion in
the virtual setting
isn’t very fluid.
Picture author owned.

## Slide 6
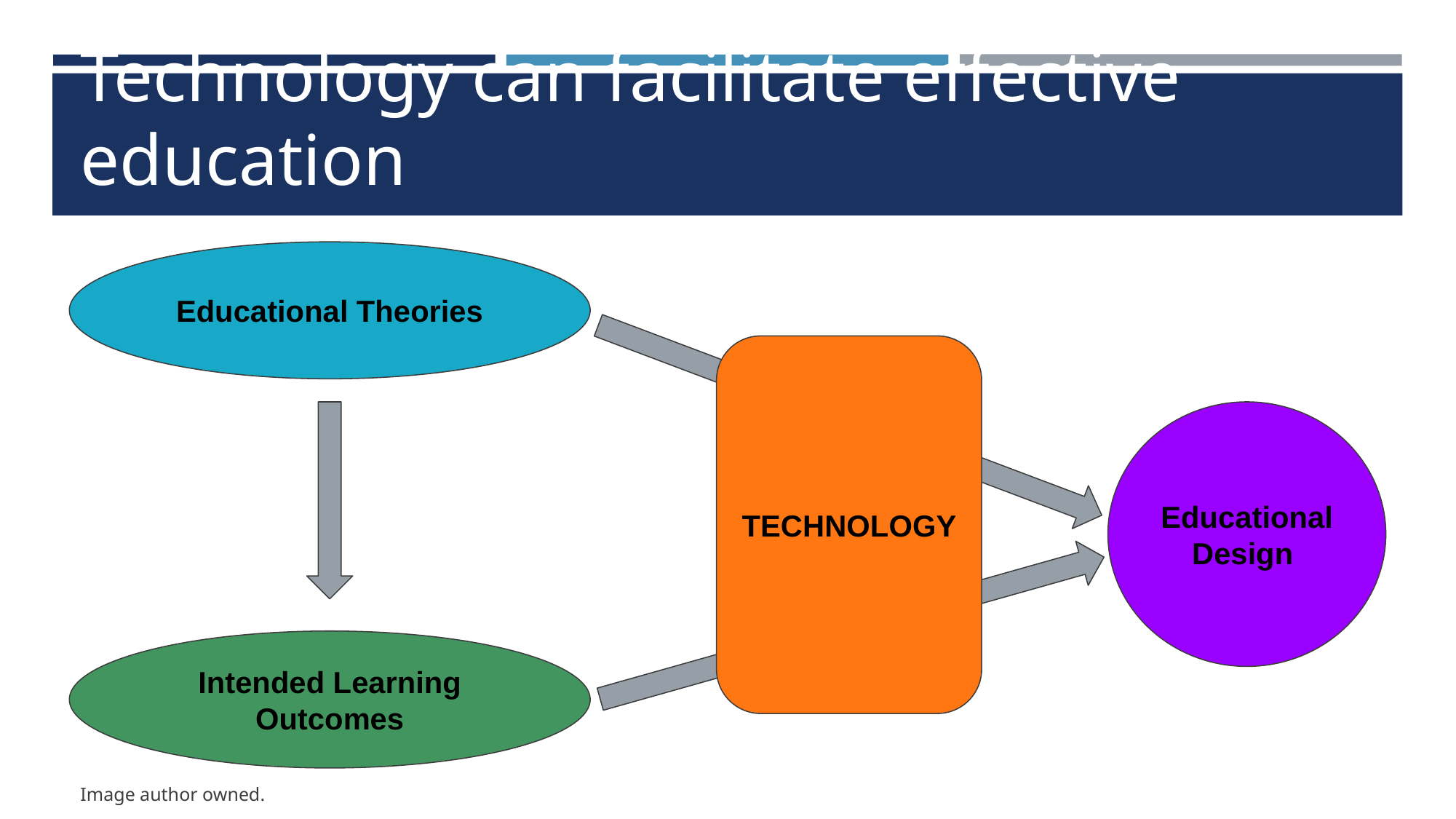

# Technology can facilitate effective education
Educational Theories
TECHNOLOGY
Vestibulum congue tempus
Lorem ipsum dolor sit amet, consectetur adipiscing elit, sed do eiusmod tempor.
Educational Design
Intended Learning Outcomes
Image author owned.

## Slide 7
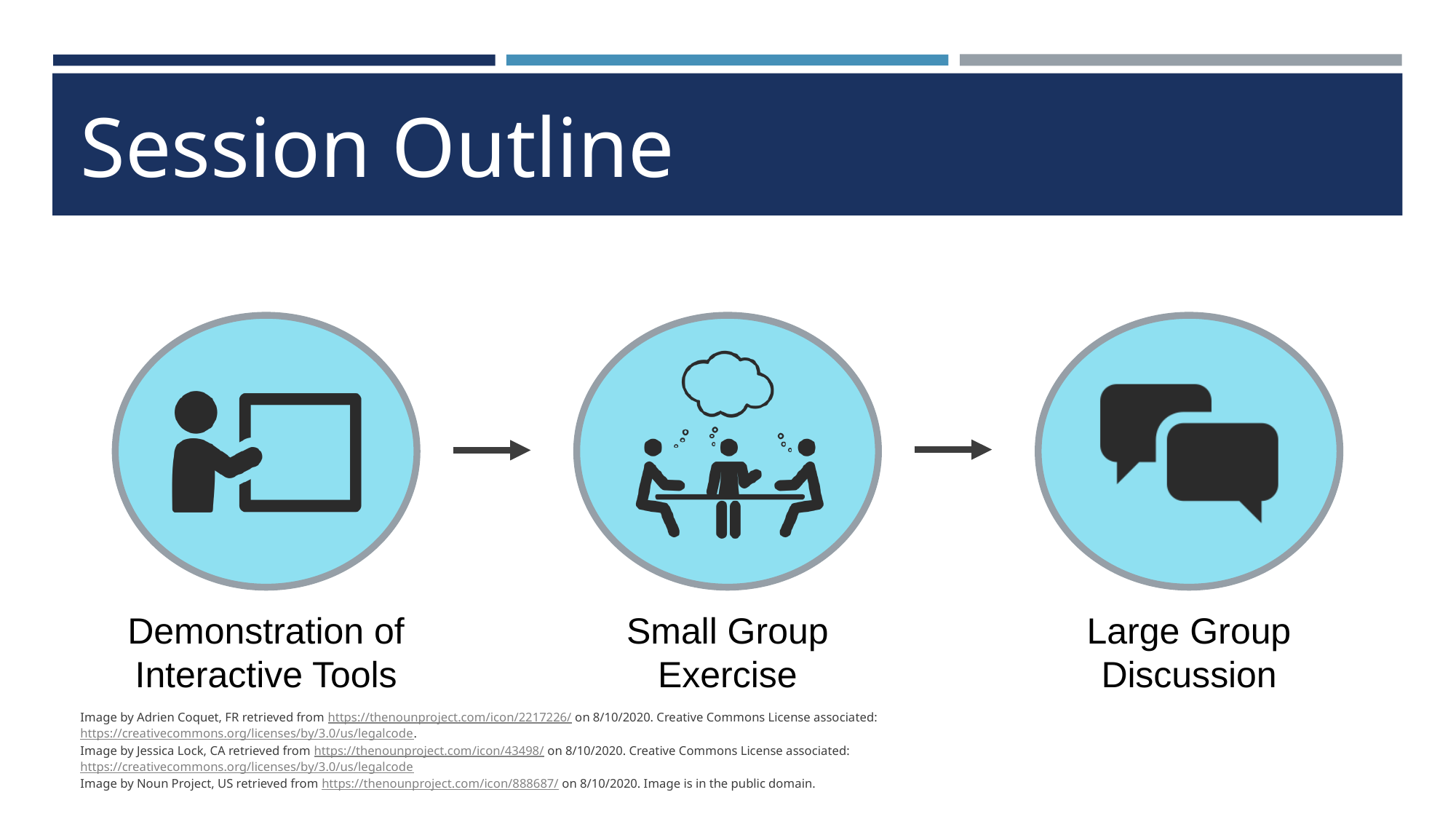

# Session Outline
Demonstration of Interactive Tools
Small Group Exercise
Large Group Discussion
Image by Adrien Coquet, FR retrieved from https://thenounproject.com/icon/2217226/ on 8/10/2020. Creative Commons License associated: https://creativecommons.org/licenses/by/3.0/us/legalcode.
Image by Jessica Lock, CA retrieved from https://thenounproject.com/icon/43498/ on 8/10/2020. Creative Commons License associated: https://creativecommons.org/licenses/by/3.0/us/legalcode
Image by Noun Project, US retrieved from https://thenounproject.com/icon/888687/ on 8/10/2020. Image is in the public domain.

## Slide 8
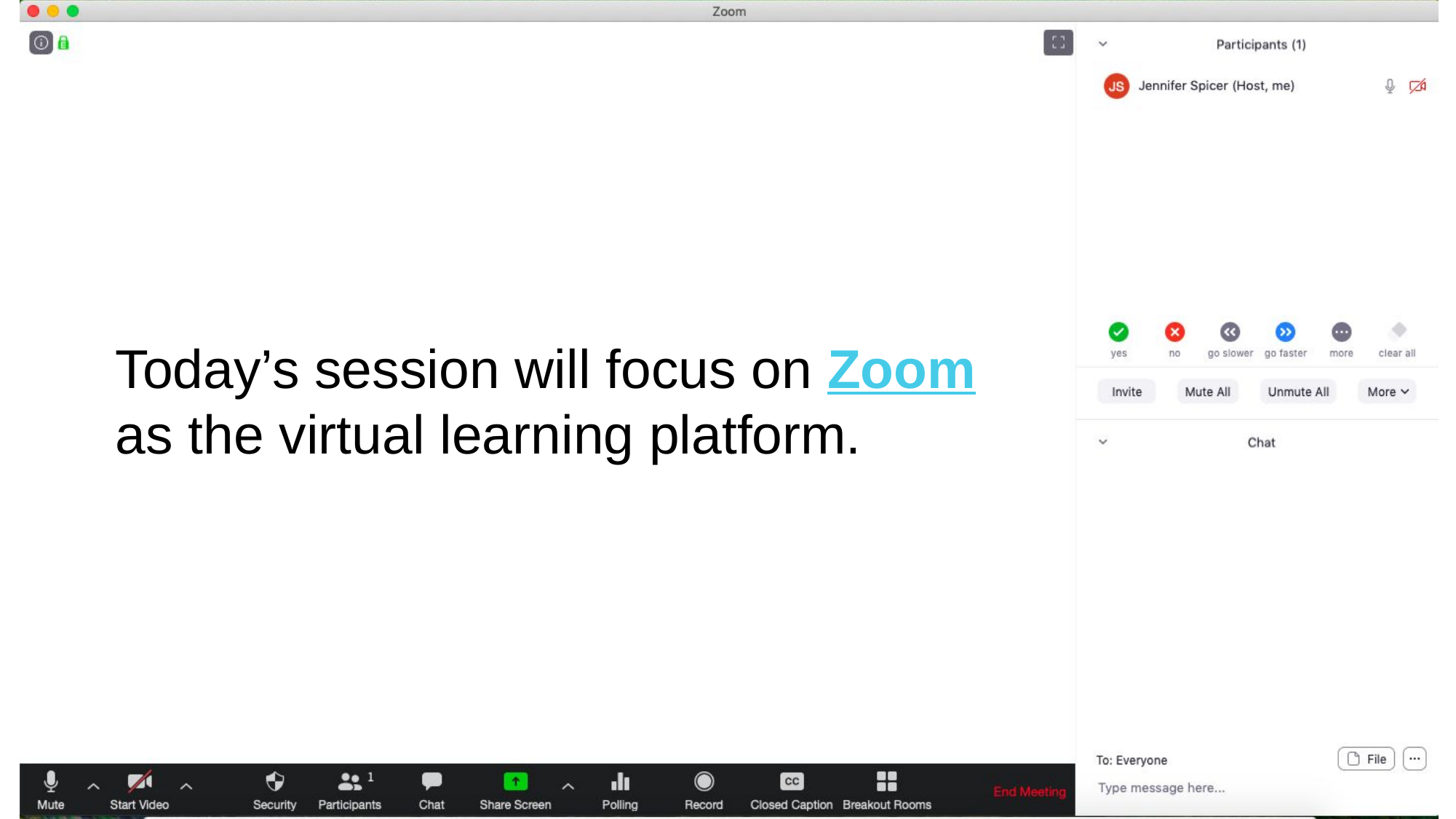

#
Today’s session will focus on Zoom as the virtual learning platform.

## Slide 9
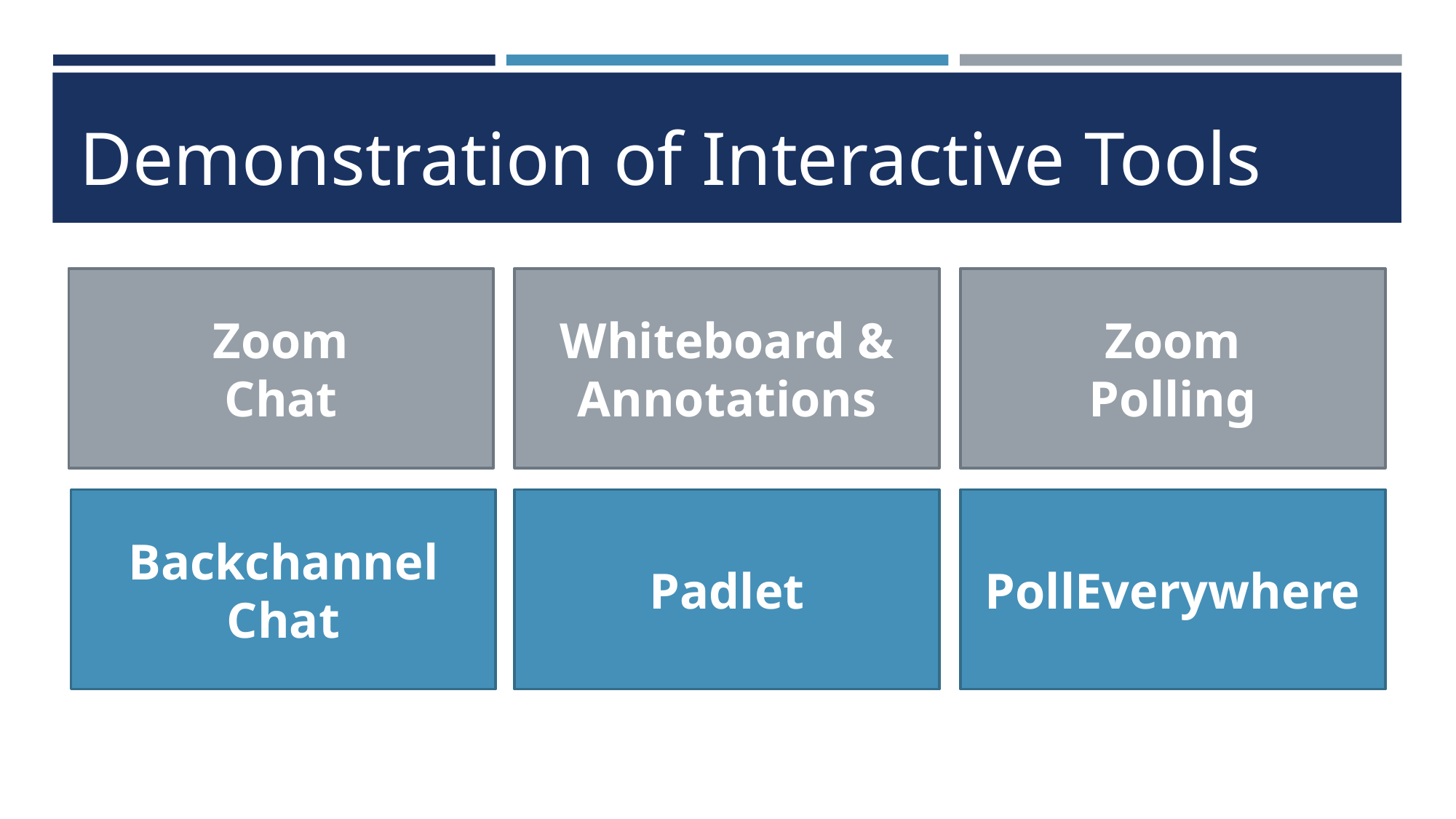

# Demonstration of Interactive Tools
Zoom
Chat
Whiteboard & Annotations
Zoom
Polling
Backchannel
Chat
Padlet
PollEverywhere

## Slide 10
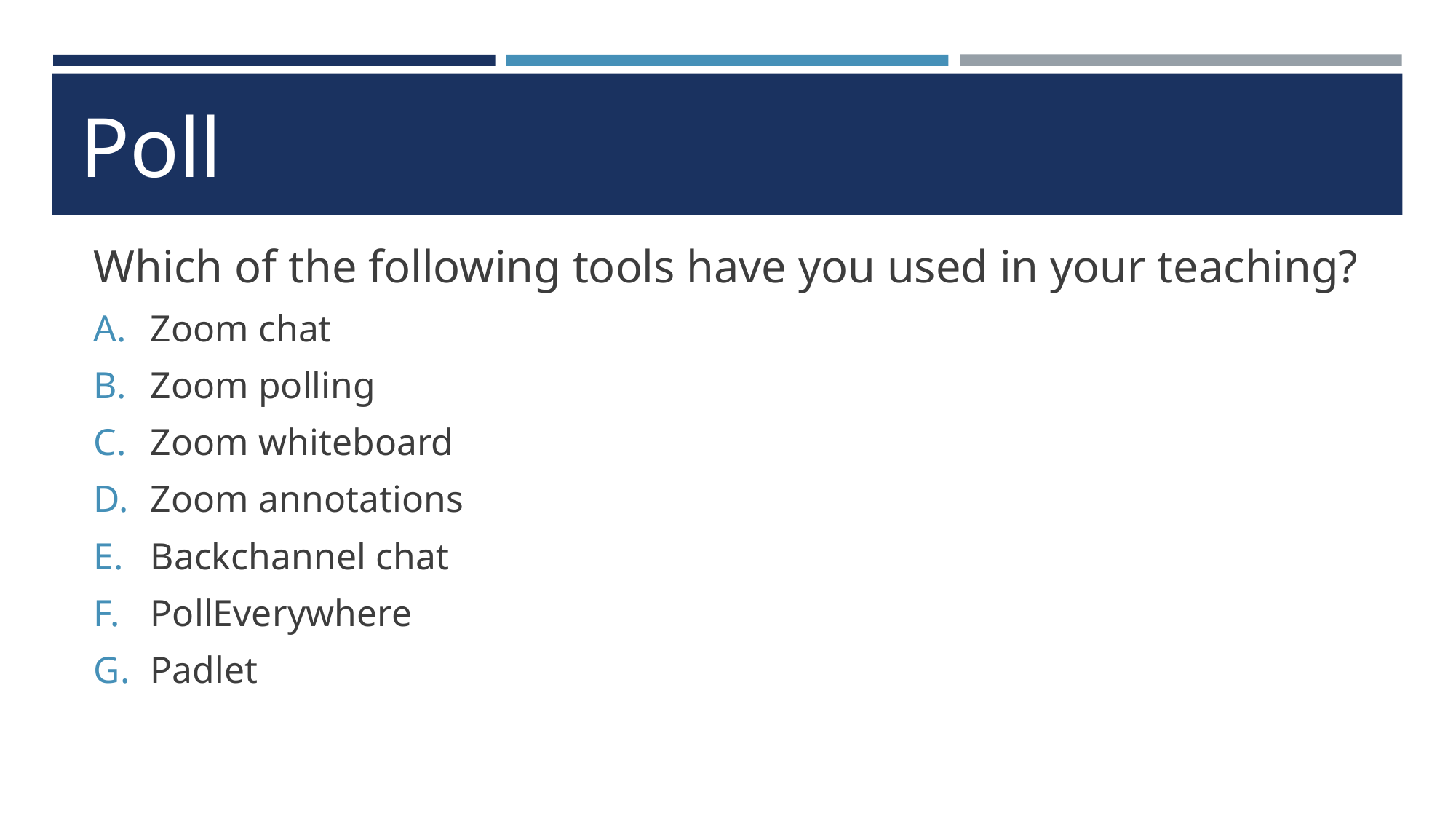

# Poll
Which of the following tools have you used in your teaching?
Zoom chat
Zoom polling
Zoom whiteboard
Zoom annotations
Backchannel chat
PollEverywhere
Padlet

## Slide 11
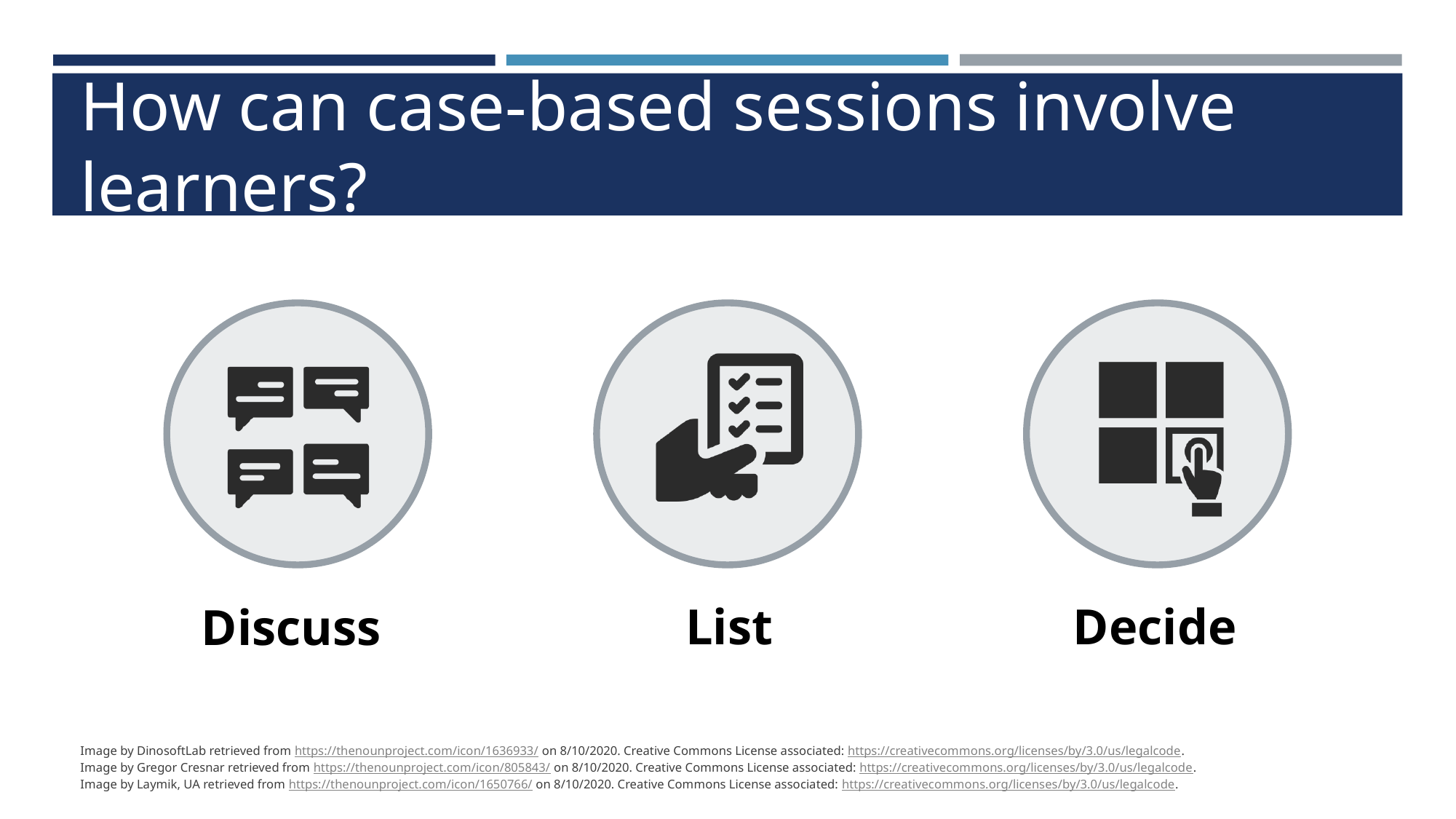

# How can case-based sessions involve learners?
Decide
List
Discuss
Image by DinosoftLab retrieved from https://thenounproject.com/icon/1636933/ on 8/10/2020. Creative Commons License associated: https://creativecommons.org/licenses/by/3.0/us/legalcode.
Image by Gregor Cresnar retrieved from https://thenounproject.com/icon/805843/ on 8/10/2020. Creative Commons License associated: https://creativecommons.org/licenses/by/3.0/us/legalcode.
Image by Laymik, UA retrieved from https://thenounproject.com/icon/1650766/ on 8/10/2020. Creative Commons License associated: https://creativecommons.org/licenses/by/3.0/us/legalcode.

## Slide 12
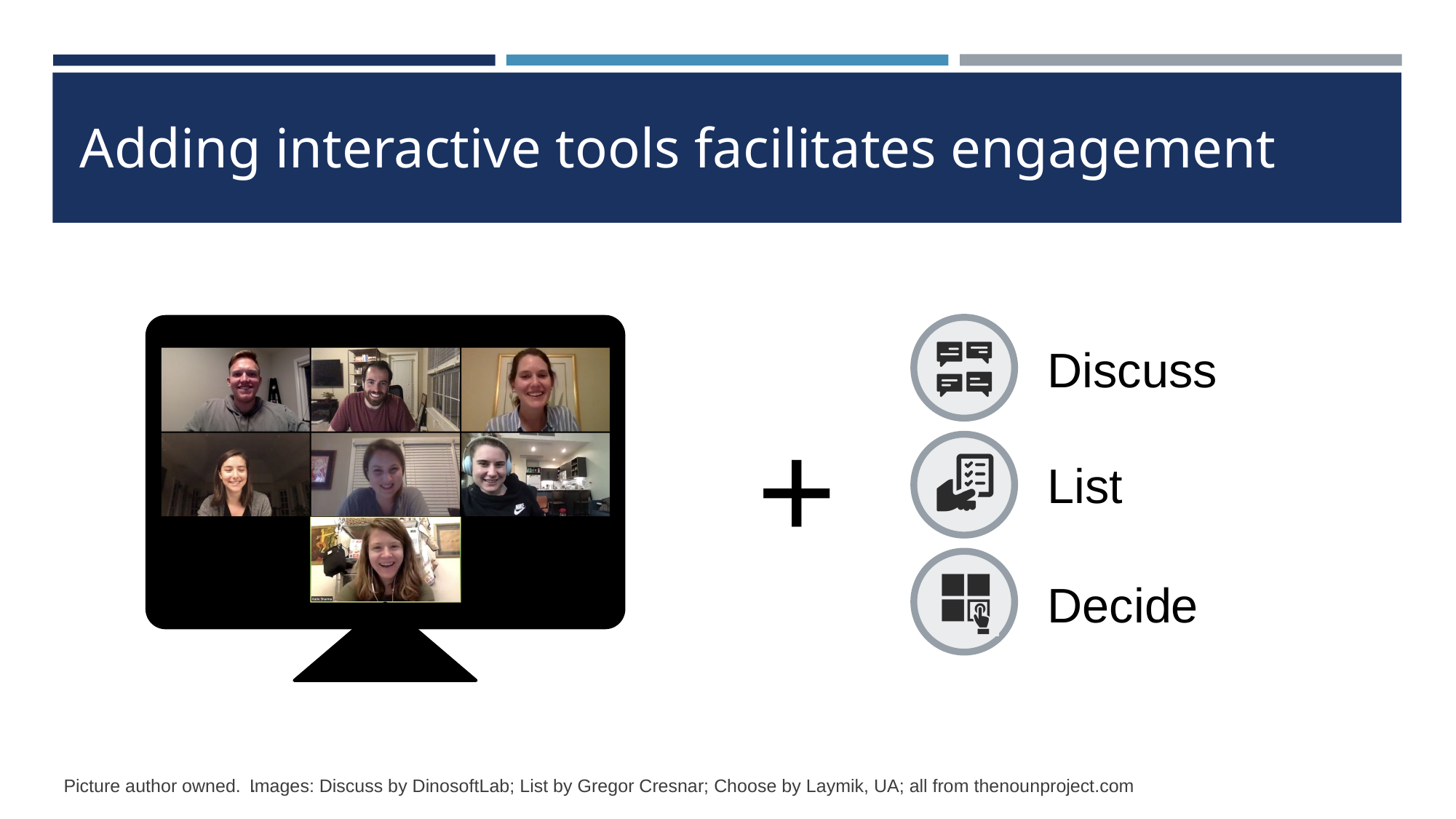

# Adding interactive tools facilitates engagement
Discuss
+
List
Decide
Images: Discuss by DinosoftLab; List by Gregor Cresnar; Choose by Laymik, UA; all from thenounproject.com
Picture author owned. .

## Slide 13
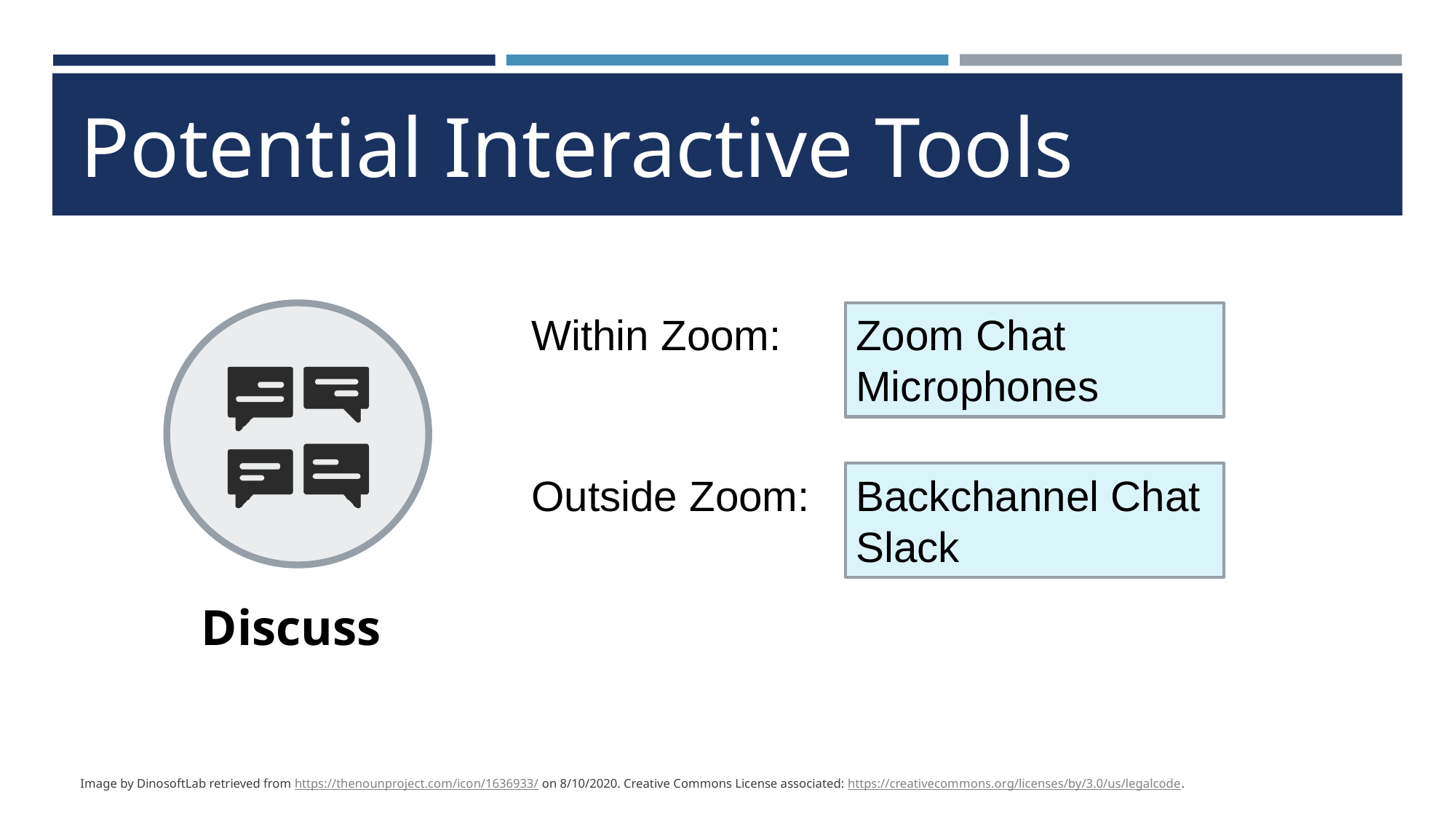

# Potential Interactive Tools
Within Zoom:
Zoom Chat
Microphones
Outside Zoom:
Backchannel Chat
Slack
Discuss
Image by DinosoftLab retrieved from https://thenounproject.com/icon/1636933/ on 8/10/2020. Creative Commons License associated: https://creativecommons.org/licenses/by/3.0/us/legalcode.

## Slide 14
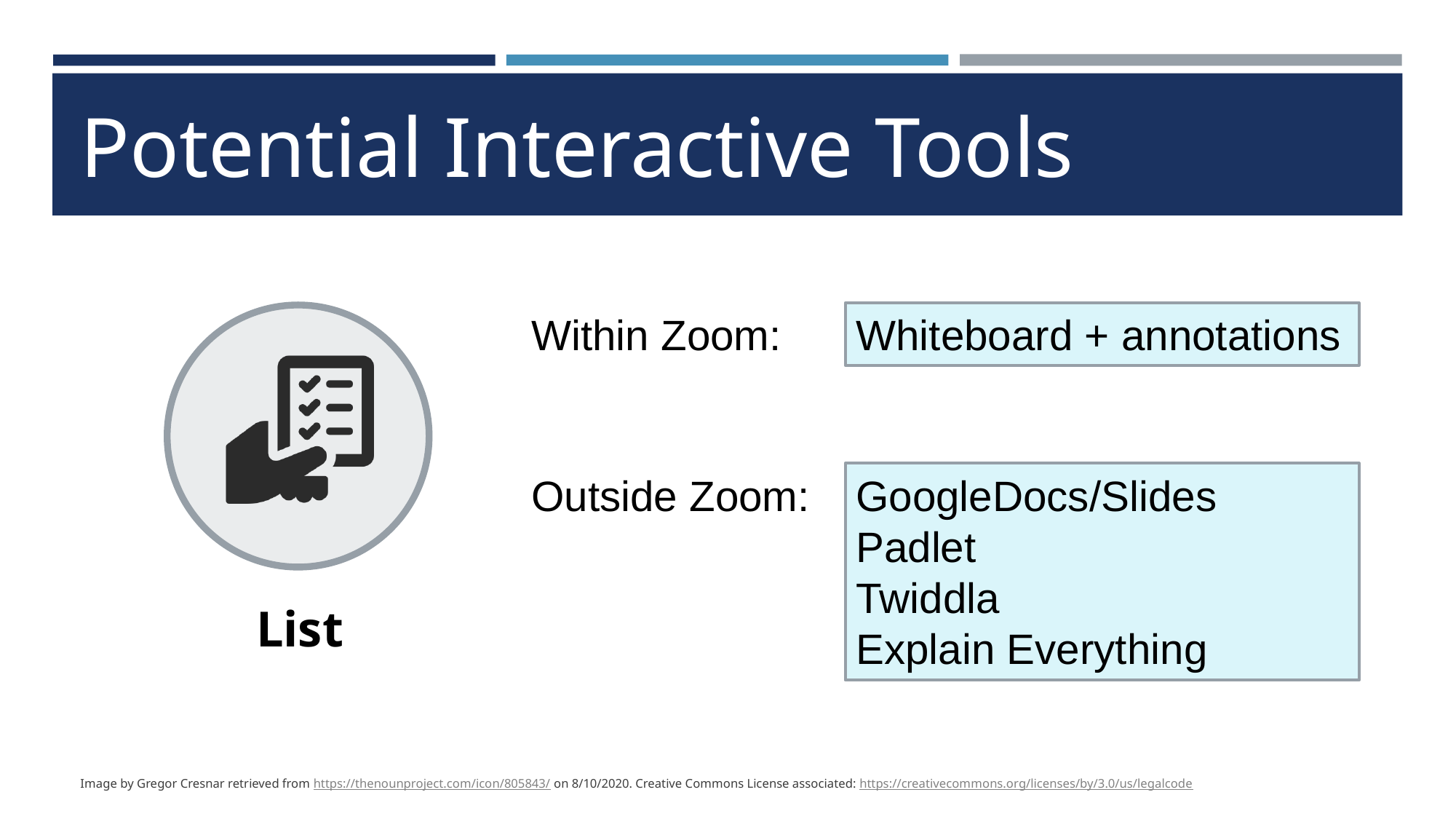

# Potential Interactive Tools
Within Zoom:
Whiteboard + annotations
Outside Zoom:
GoogleDocs/Slides
Padlet
Twiddla
Explain Everything
List
Image by Gregor Cresnar retrieved from https://thenounproject.com/icon/805843/ on 8/10/2020. Creative Commons License associated: https://creativecommons.org/licenses/by/3.0/us/legalcode

## Slide 15
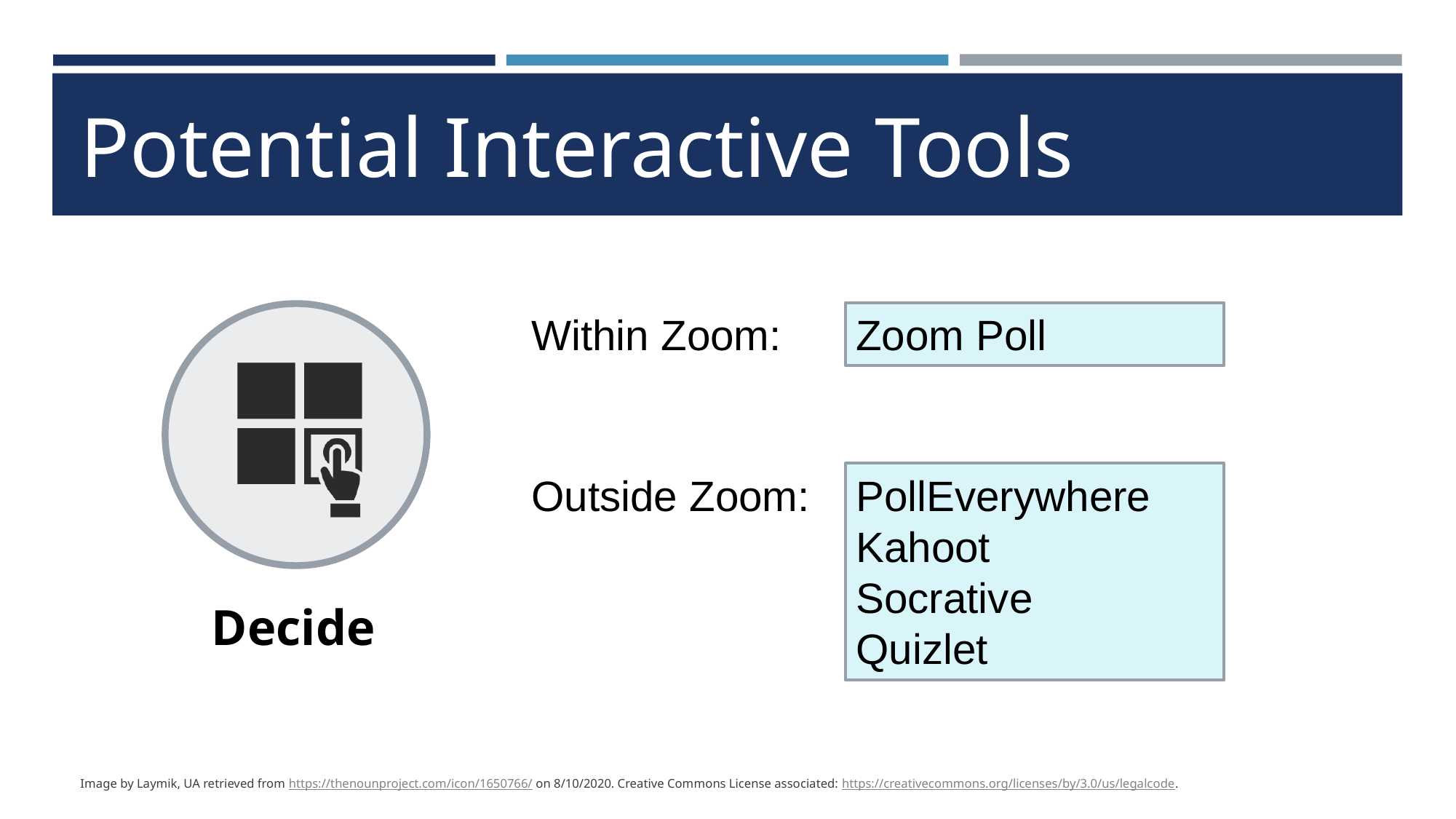

# Potential Interactive Tools
Within Zoom:
Zoom Poll
Outside Zoom:
PollEverywhere
Kahoot
Socrative
Quizlet
Decide
Image by Laymik, UA retrieved from https://thenounproject.com/icon/1650766/ on 8/10/2020. Creative Commons License associated: https://creativecommons.org/licenses/by/3.0/us/legalcode.

## Slide 16
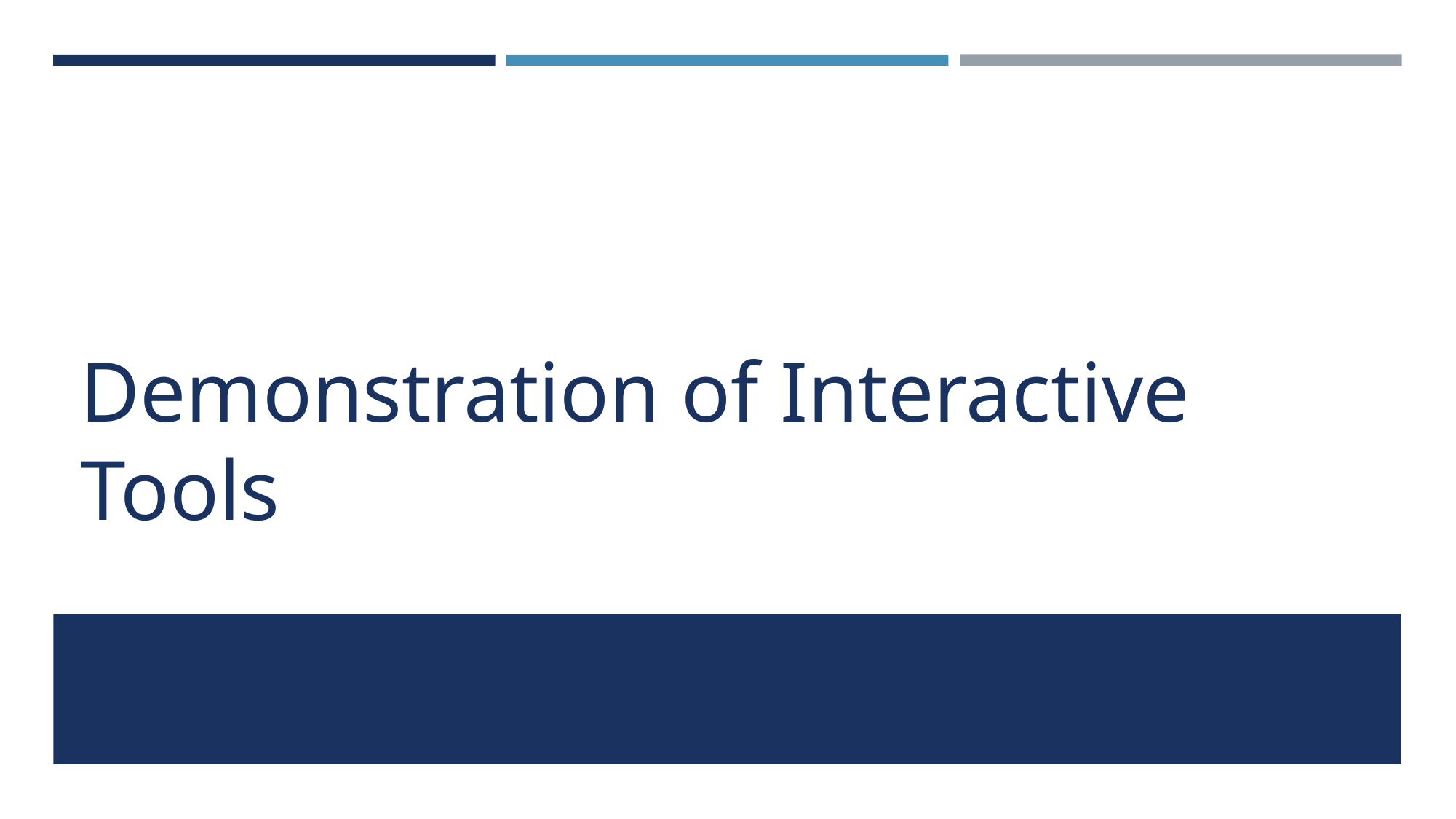

# Demonstration of Interactive Tools

## Slide 17
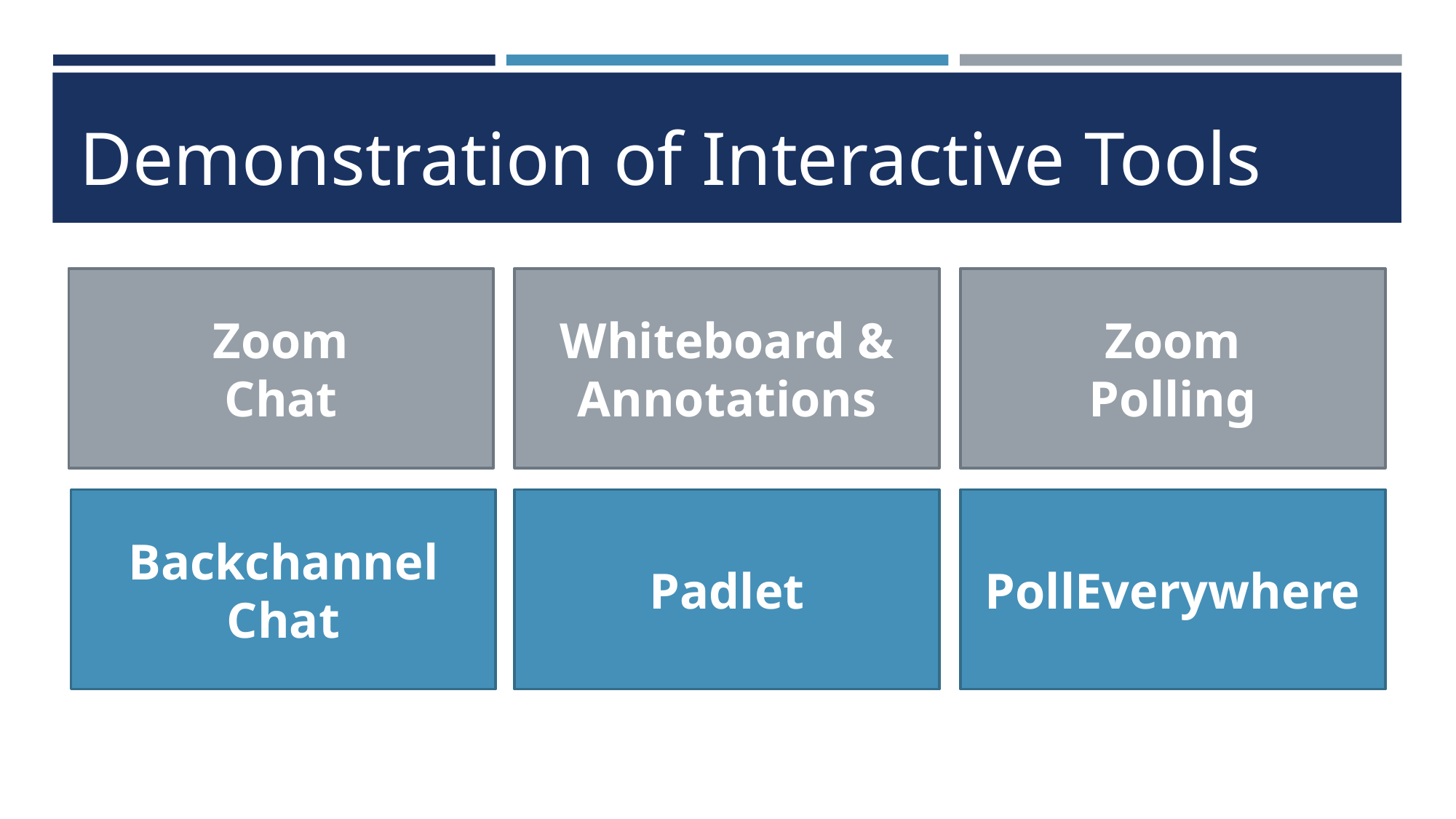

# Demonstration of Interactive Tools
Zoom
Chat
Whiteboard & Annotations
Zoom
Polling
Backchannel
Chat
Padlet
PollEverywhere

## Slide 18
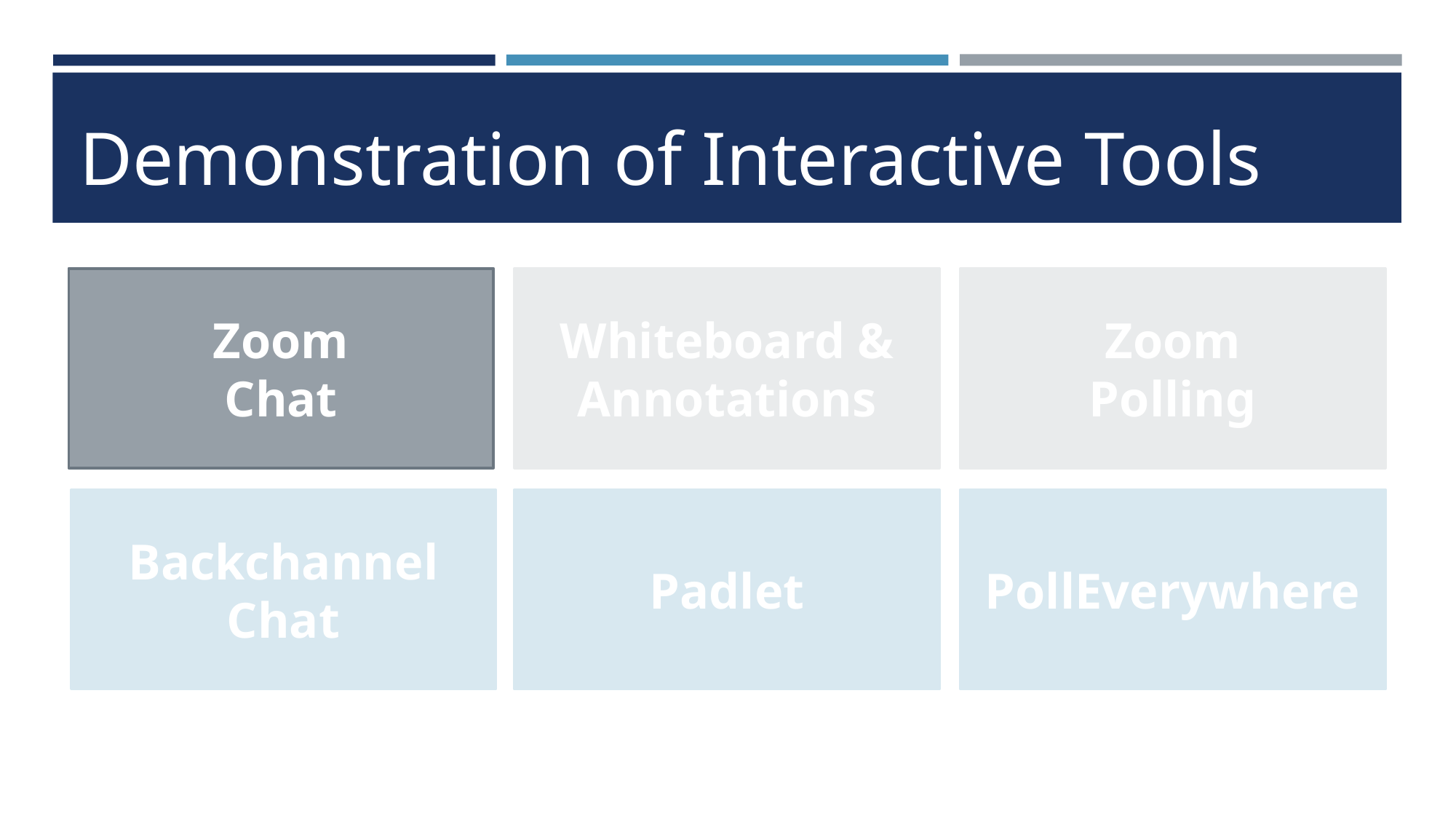

# Demonstration of Interactive Tools
Zoom
Chat
Whiteboard & Annotations
Zoom
Polling
Backchannel
Chat
Padlet
PollEverywhere

## Slide 19
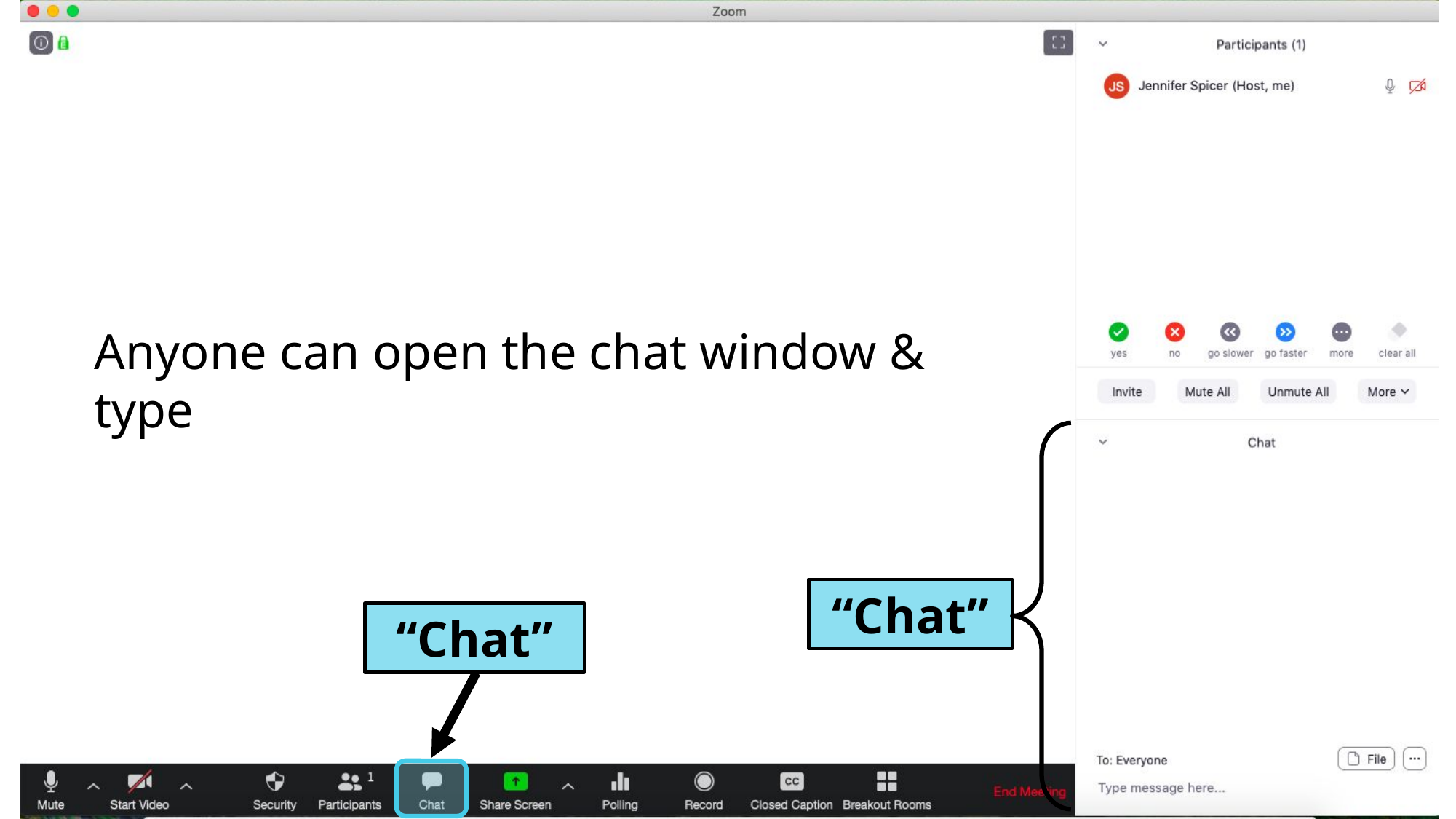

Anyone can open the chat window & type
“Chat”
“Chat”

## Slide 20
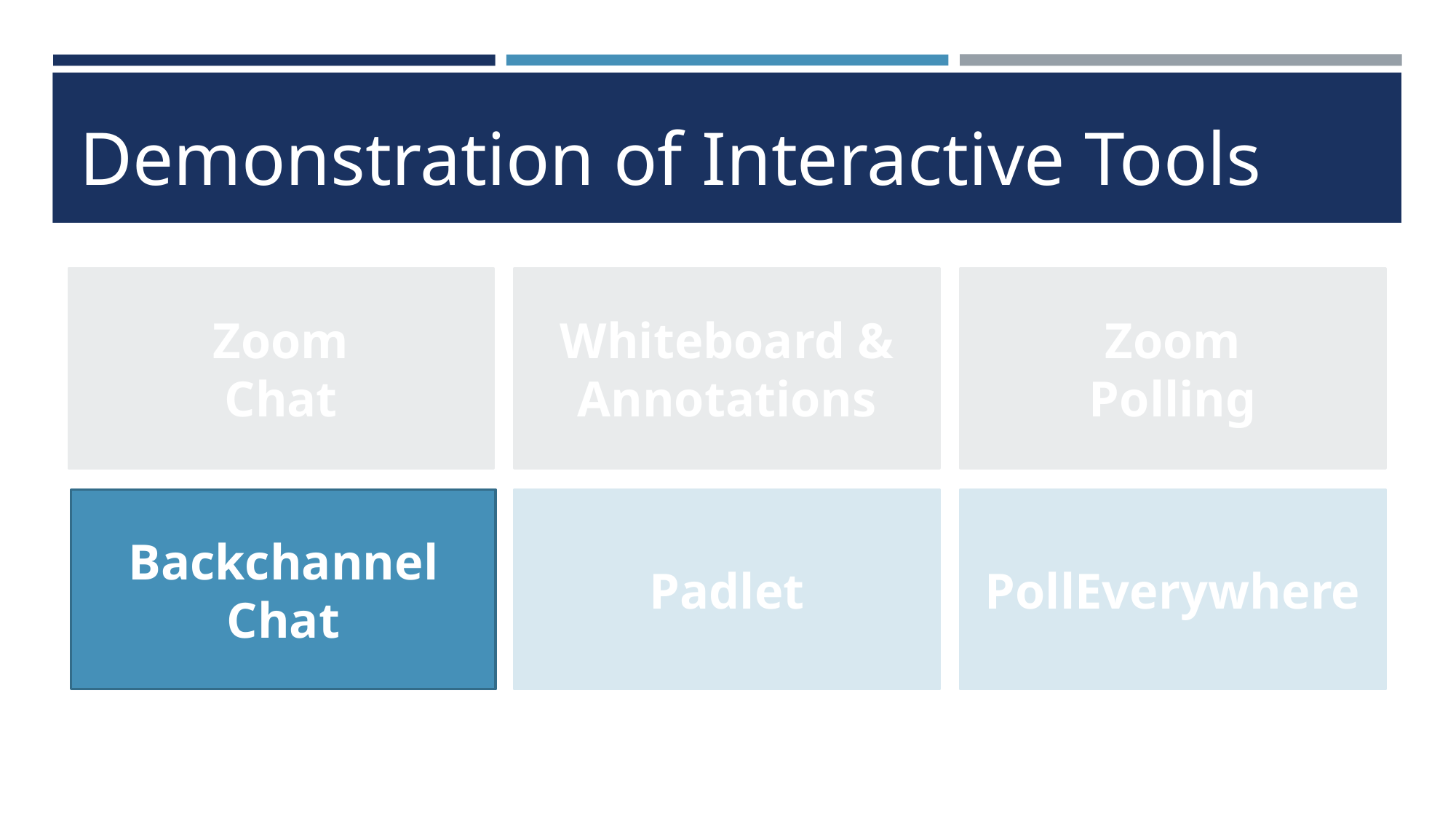

# Demonstration of Interactive Tools
Zoom
Chat
Whiteboard & Annotations
Zoom
Polling
Backchannel
Chat
Padlet
PollEverywhere

## Slide 21
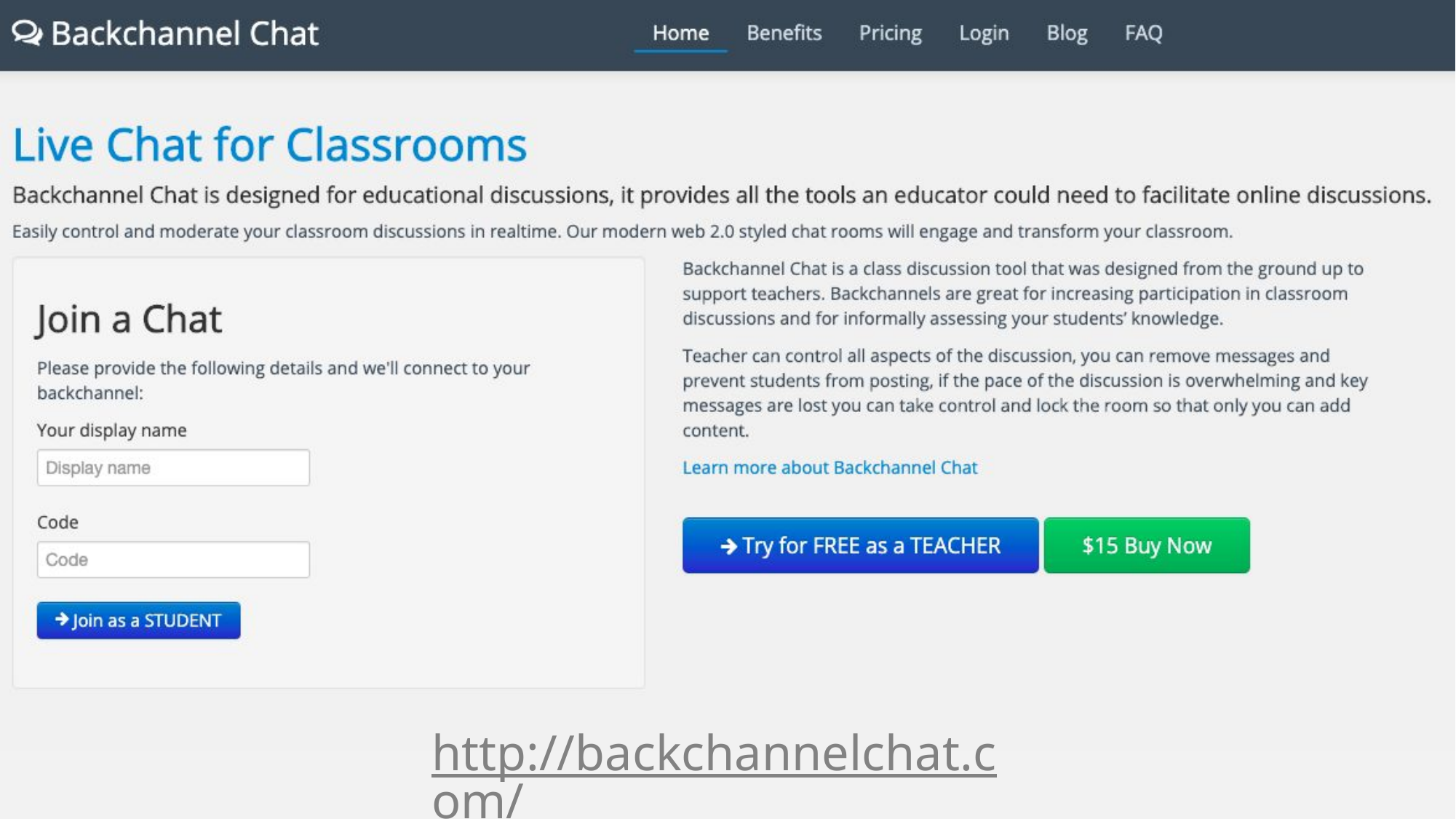

http://backchannelchat.com/

## Slide 22
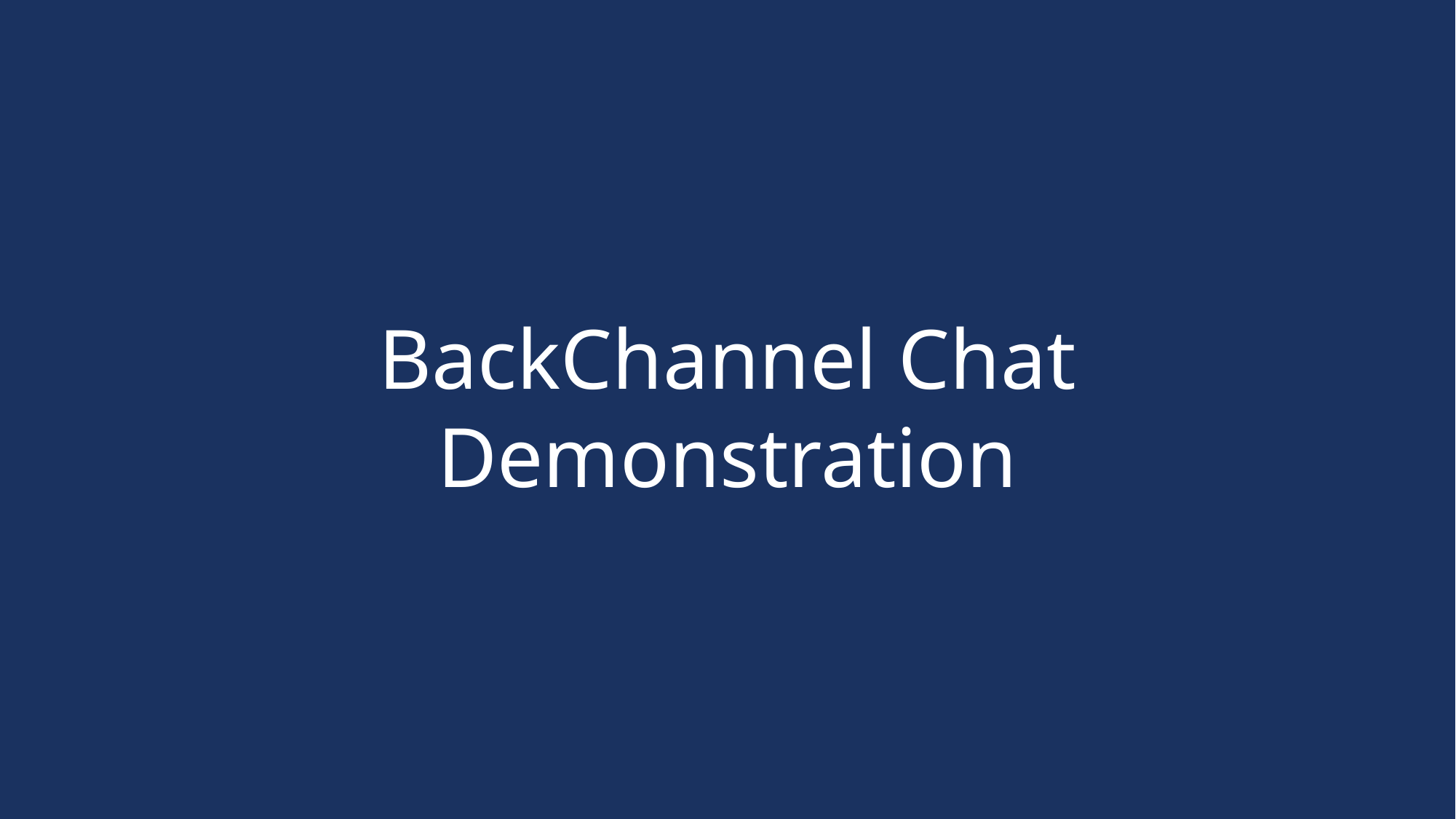

# BackChannel Chat Demonstration

## Slide 23
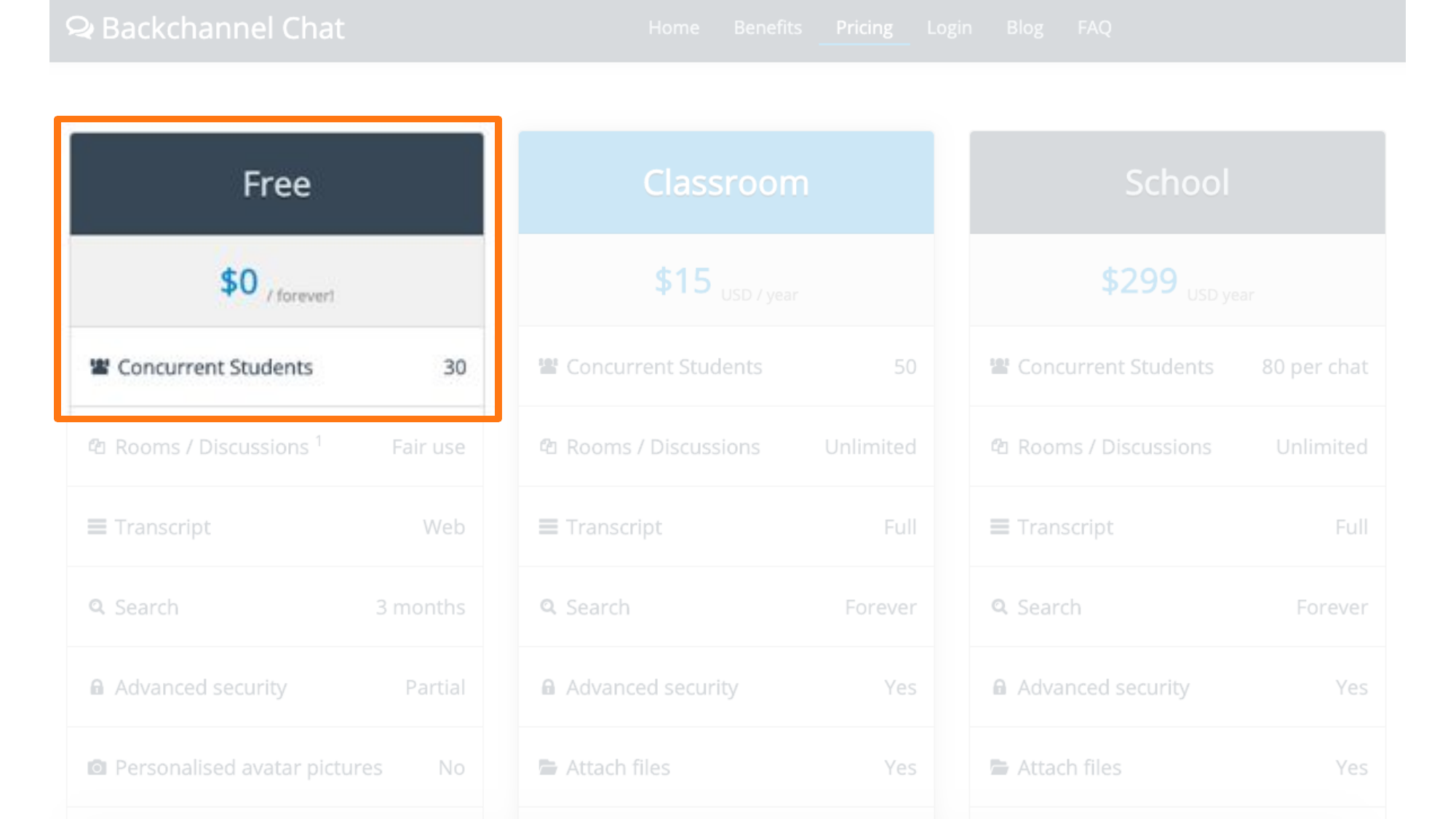

## Slide 24
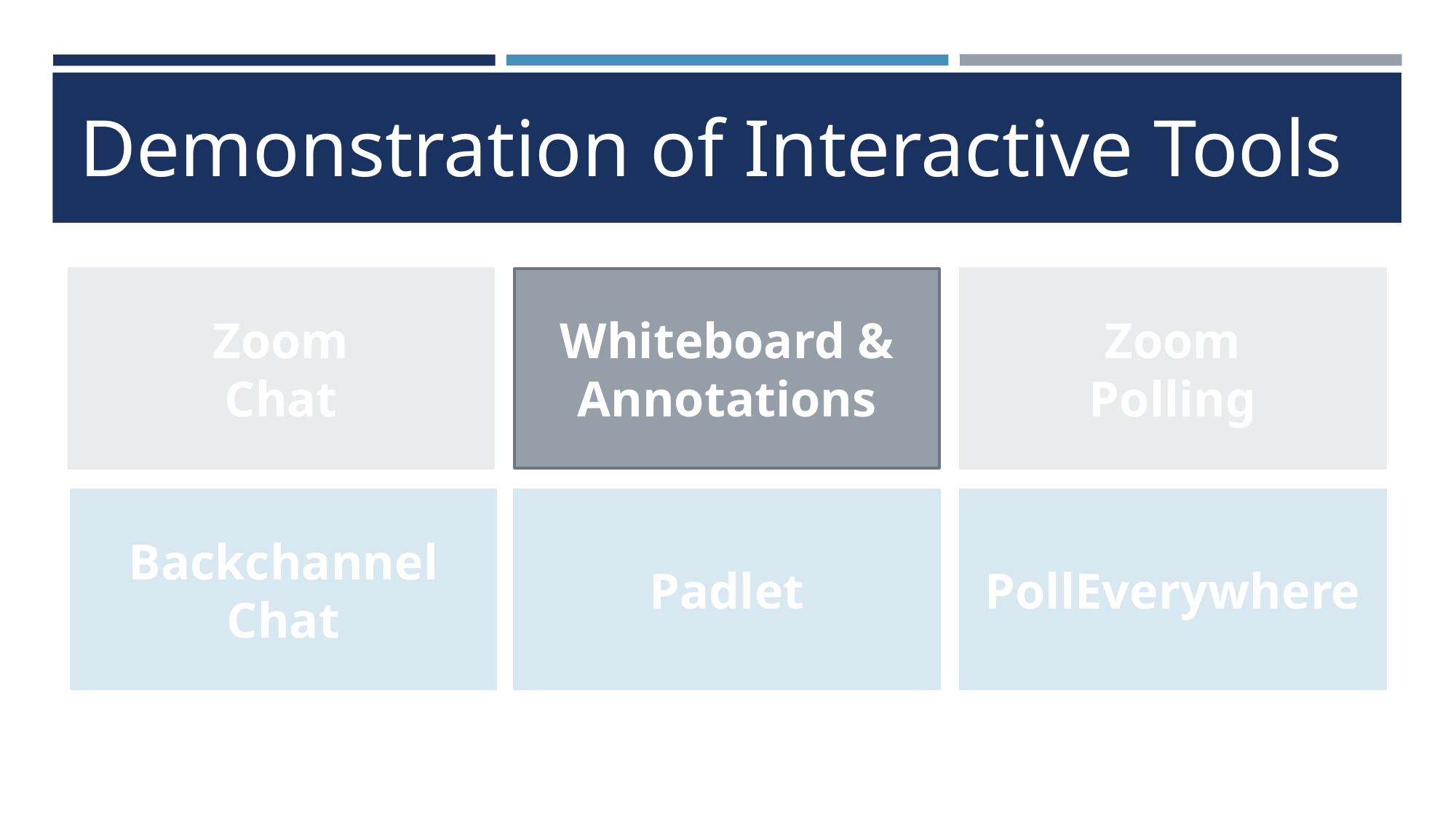

# Demonstration of Interactive Tools
Zoom
Chat
Whiteboard & Annotations
Zoom
Polling
Backchannel
Chat
Padlet
PollEverywhere

## Slide 25
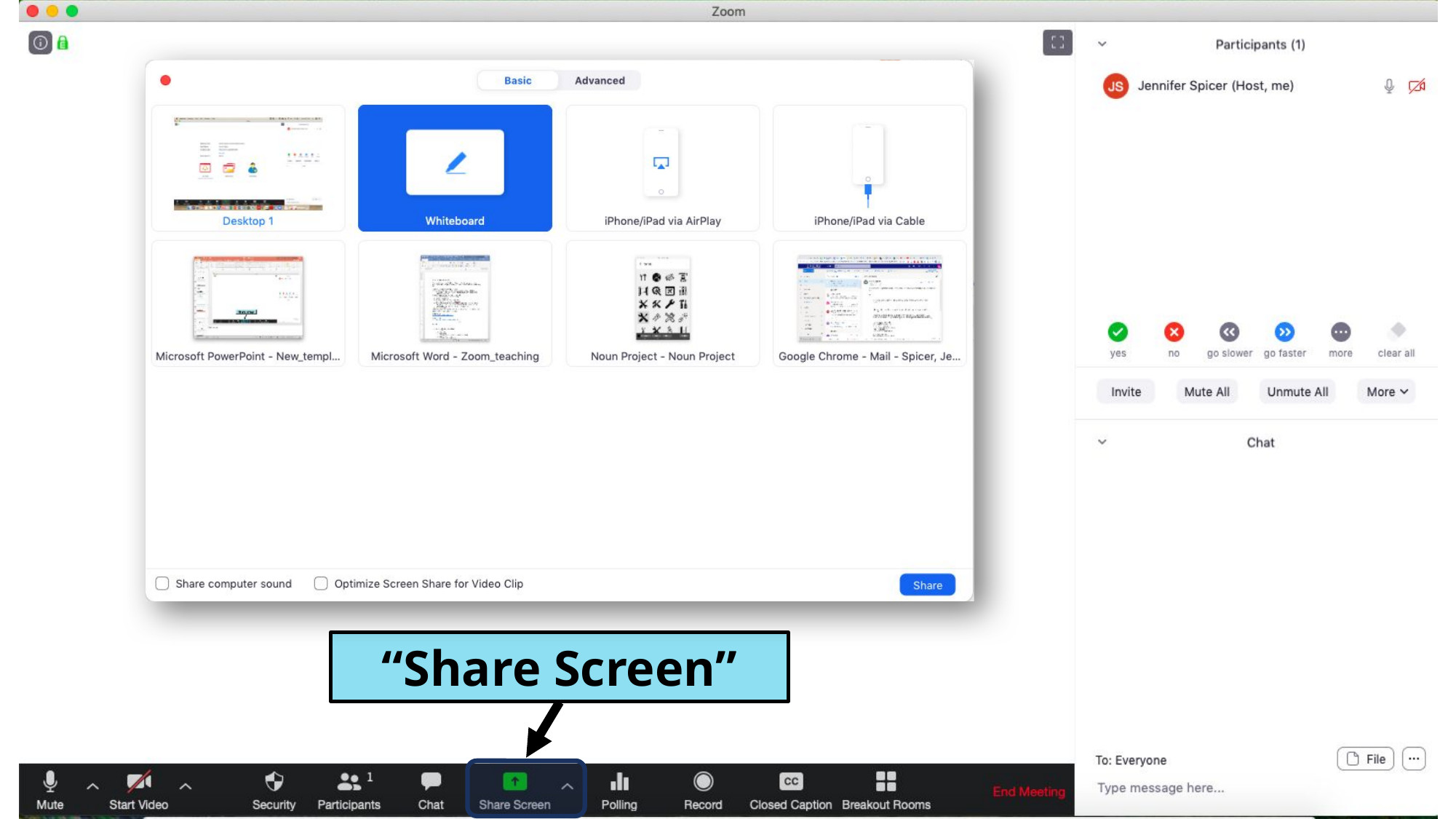

“Share Screen”

## Slide 26
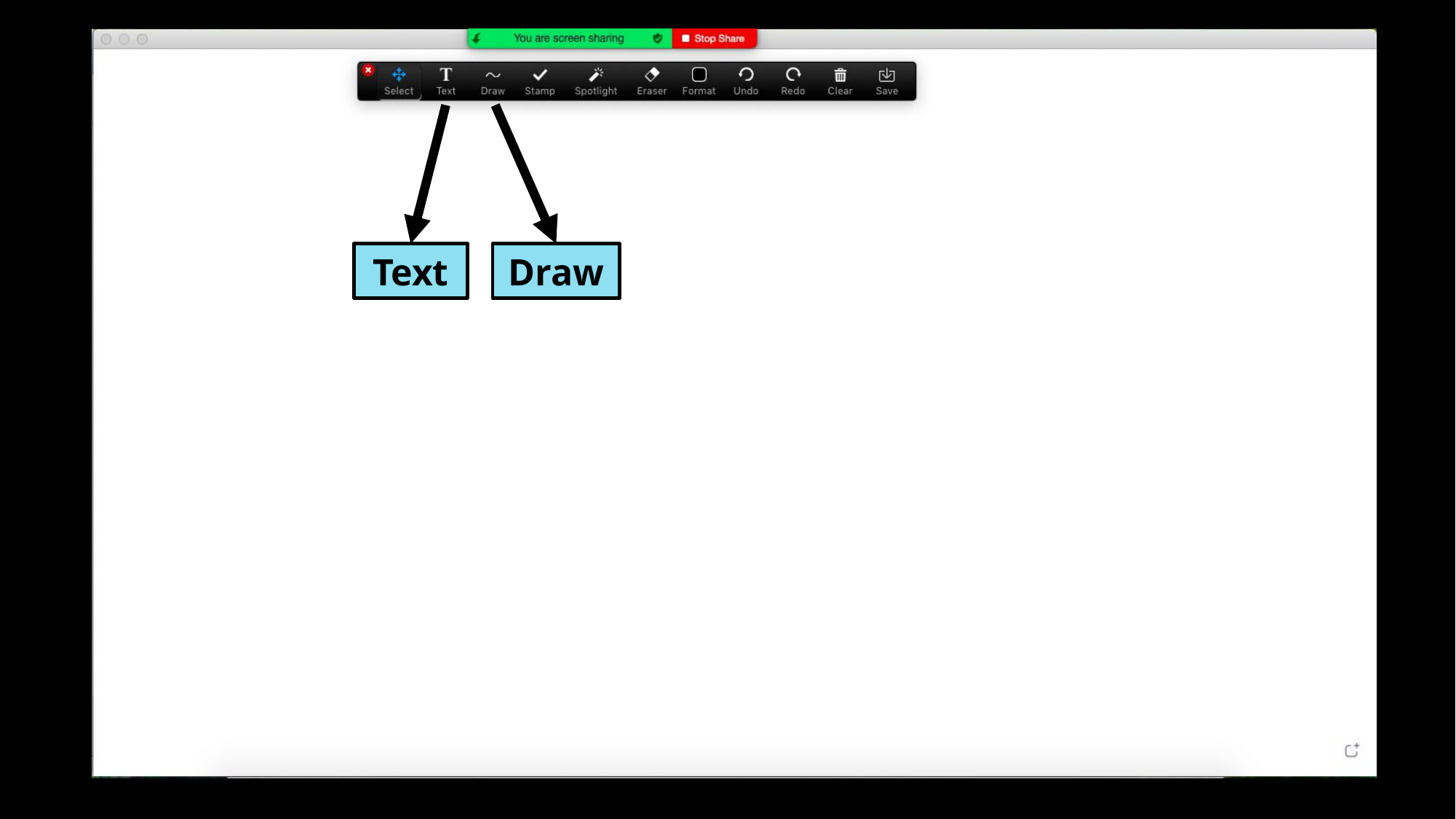

Text
Draw

## Slide 27
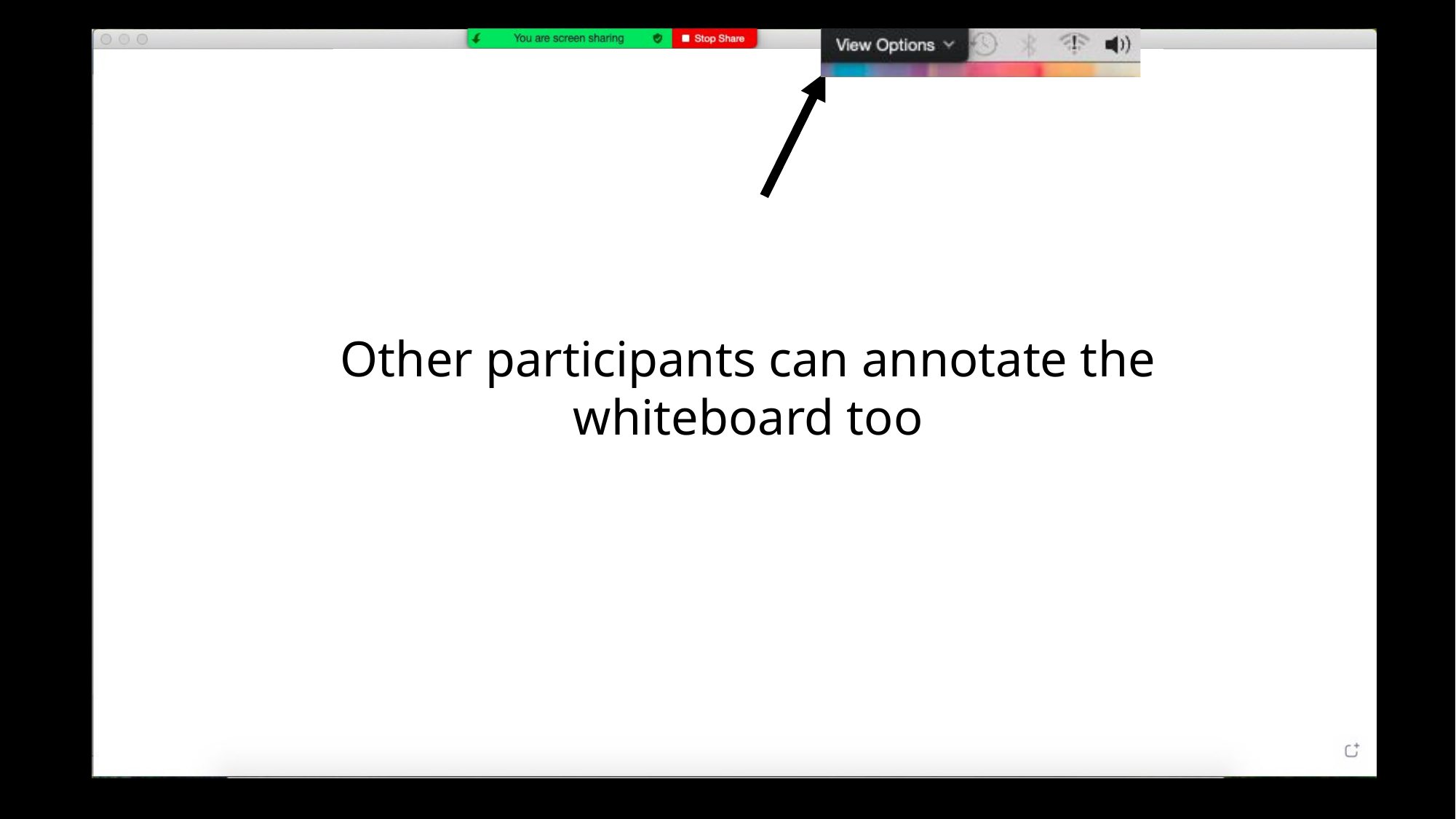

Other participants can annotate the whiteboard too

## Slide 28
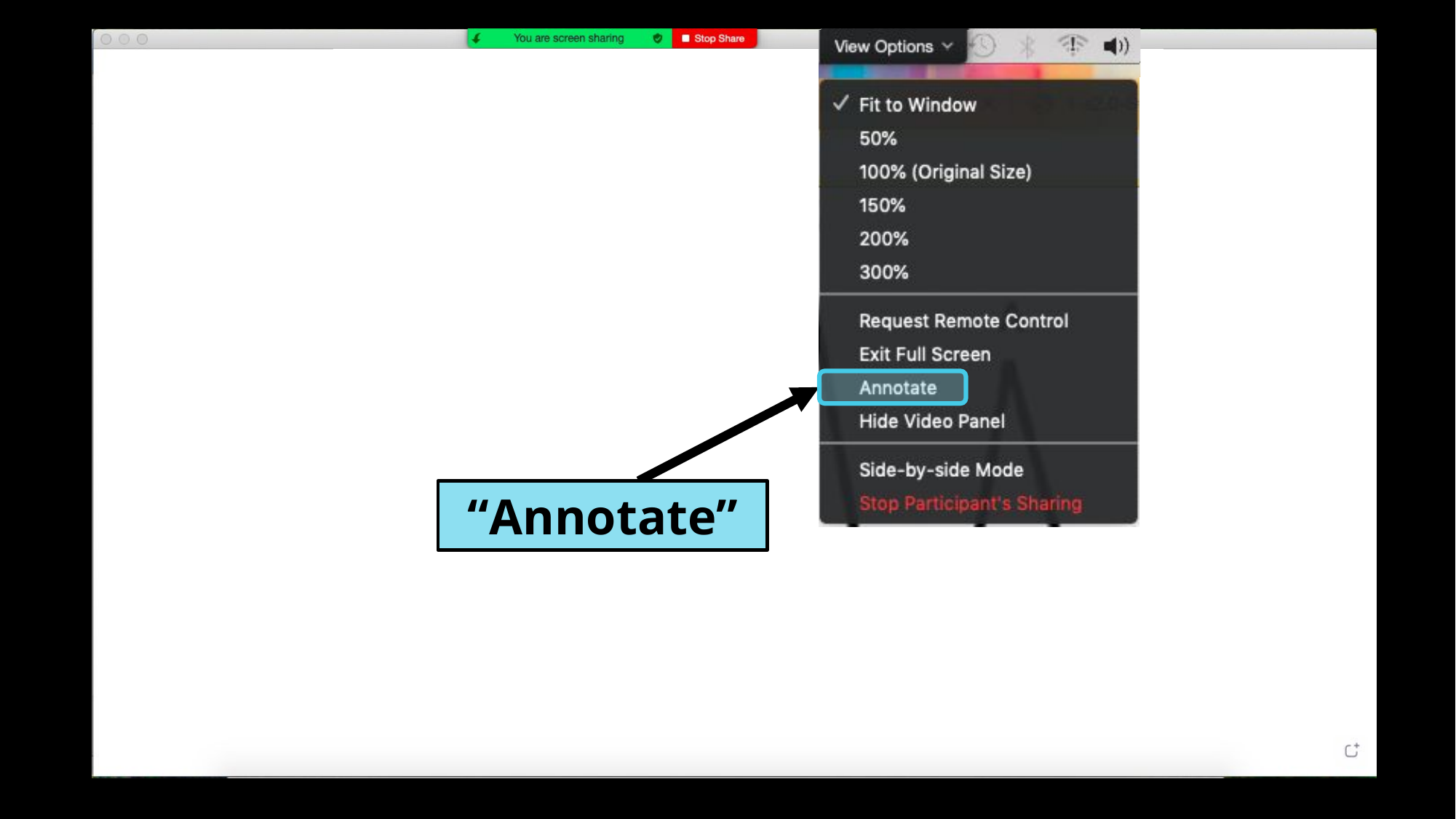

“Annotate”

## Slide 29
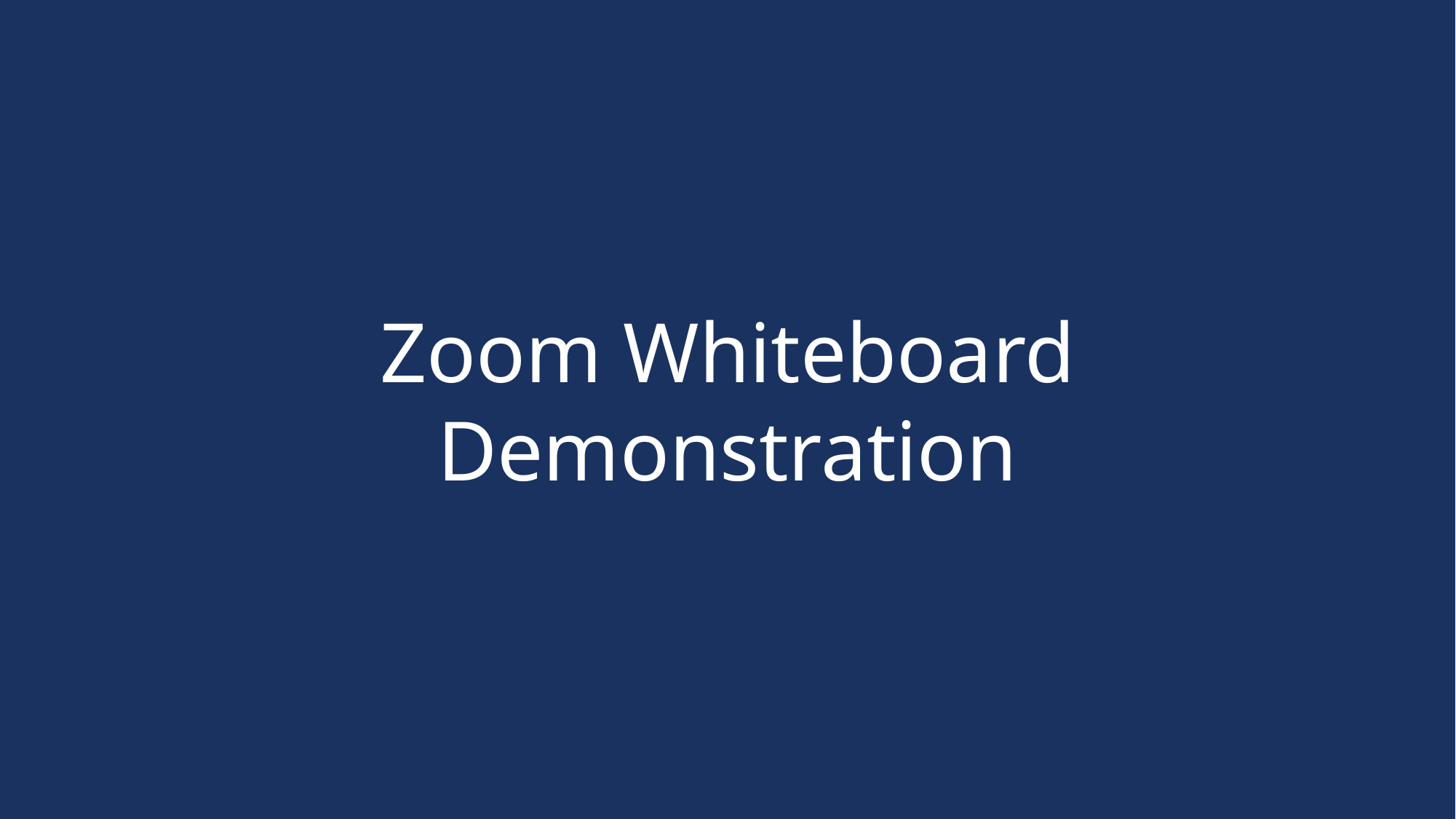

# Zoom Whiteboard Demonstration

## Slide 30
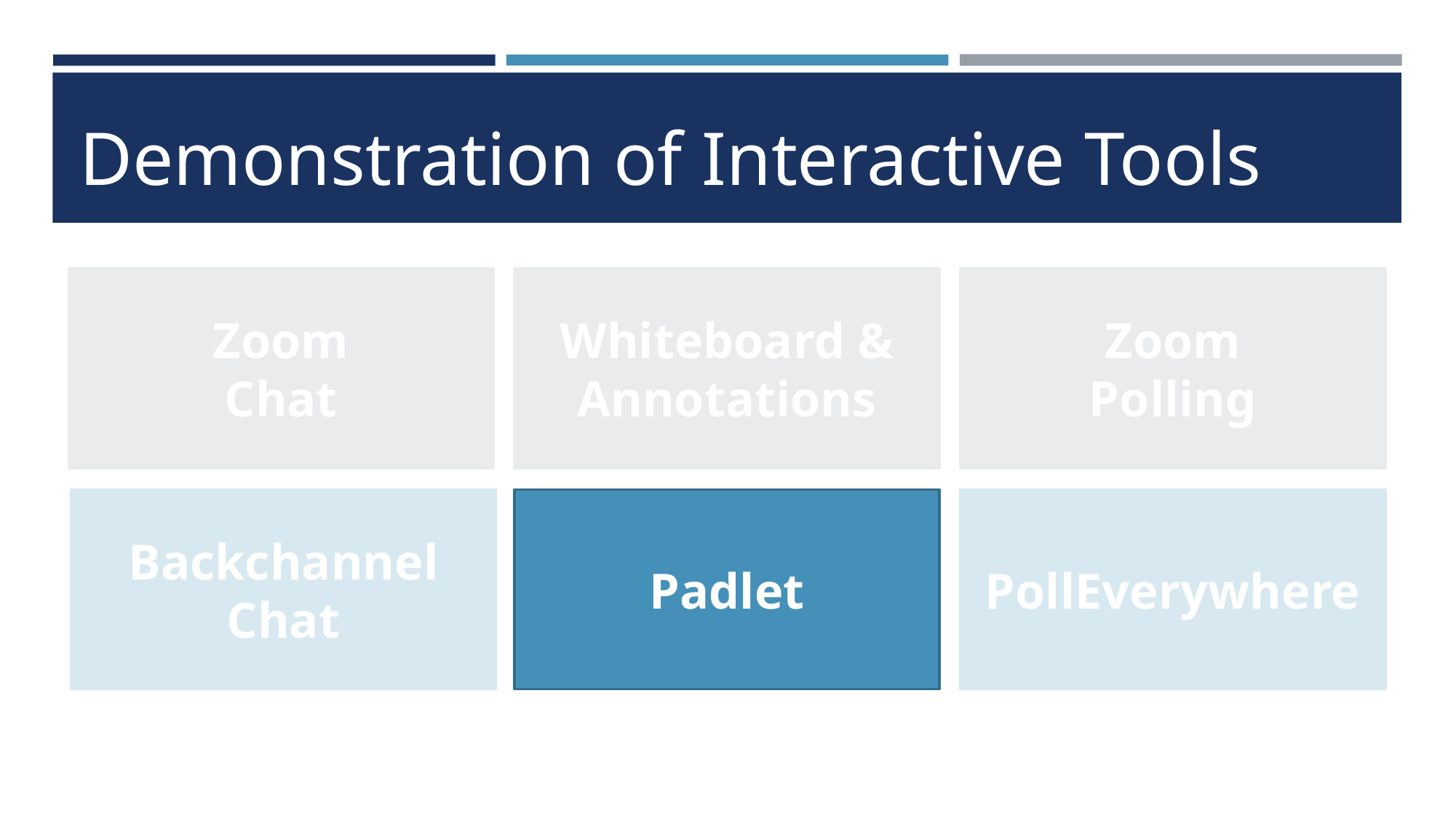

# Demonstration of Interactive Tools
Zoom
Chat
Whiteboard & Annotations
Zoom
Polling
Backchannel
Chat
Padlet
PollEverywhere

## Slide 31
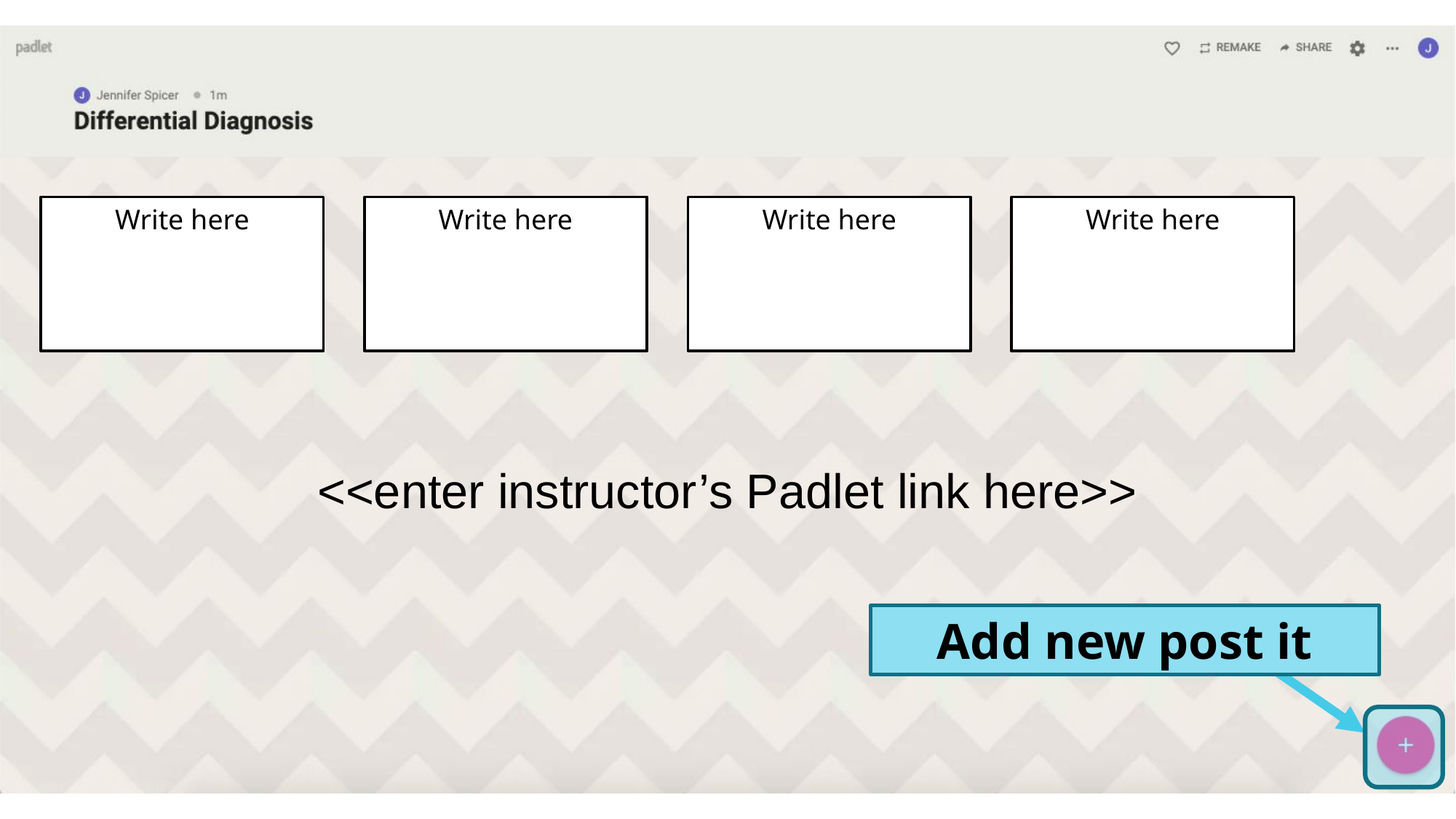

Write here
Write here
Write here
Write here
<<enter instructor’s Padlet link here>>
Add new post it

## Slide 32
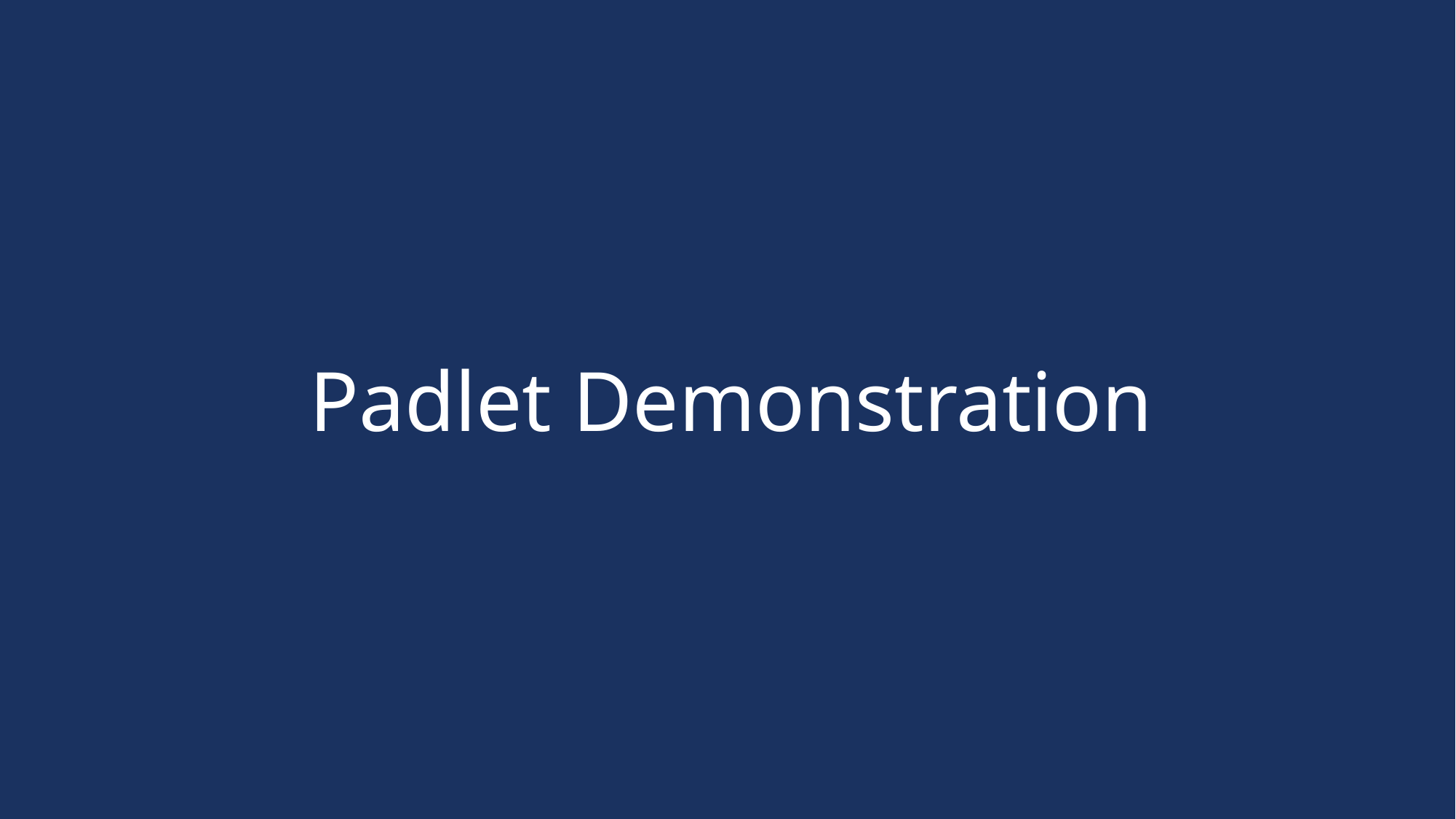

# Padlet Demonstration

## Slide 33
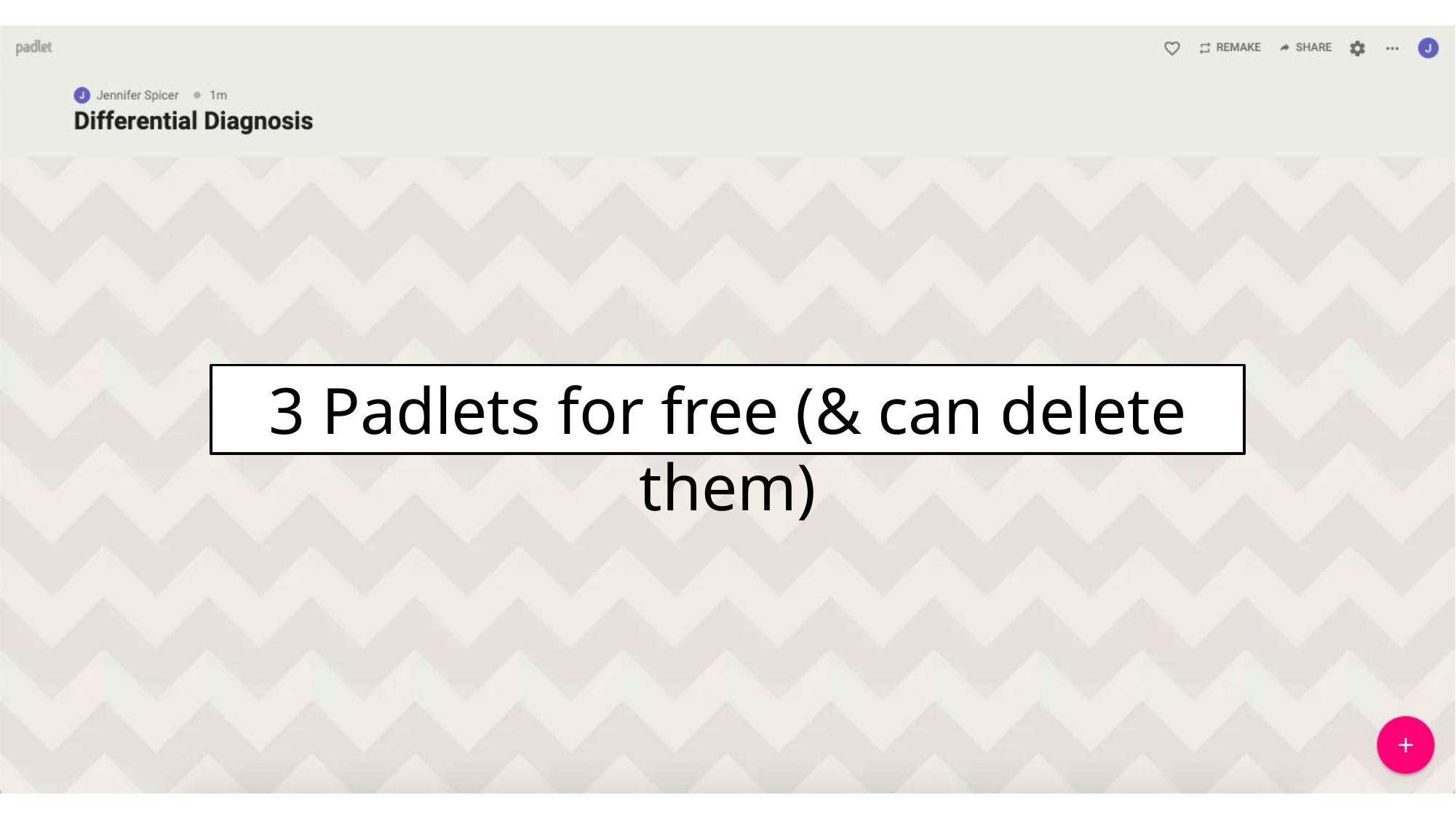

3 Padlets for free (& can delete them)

## Slide 34
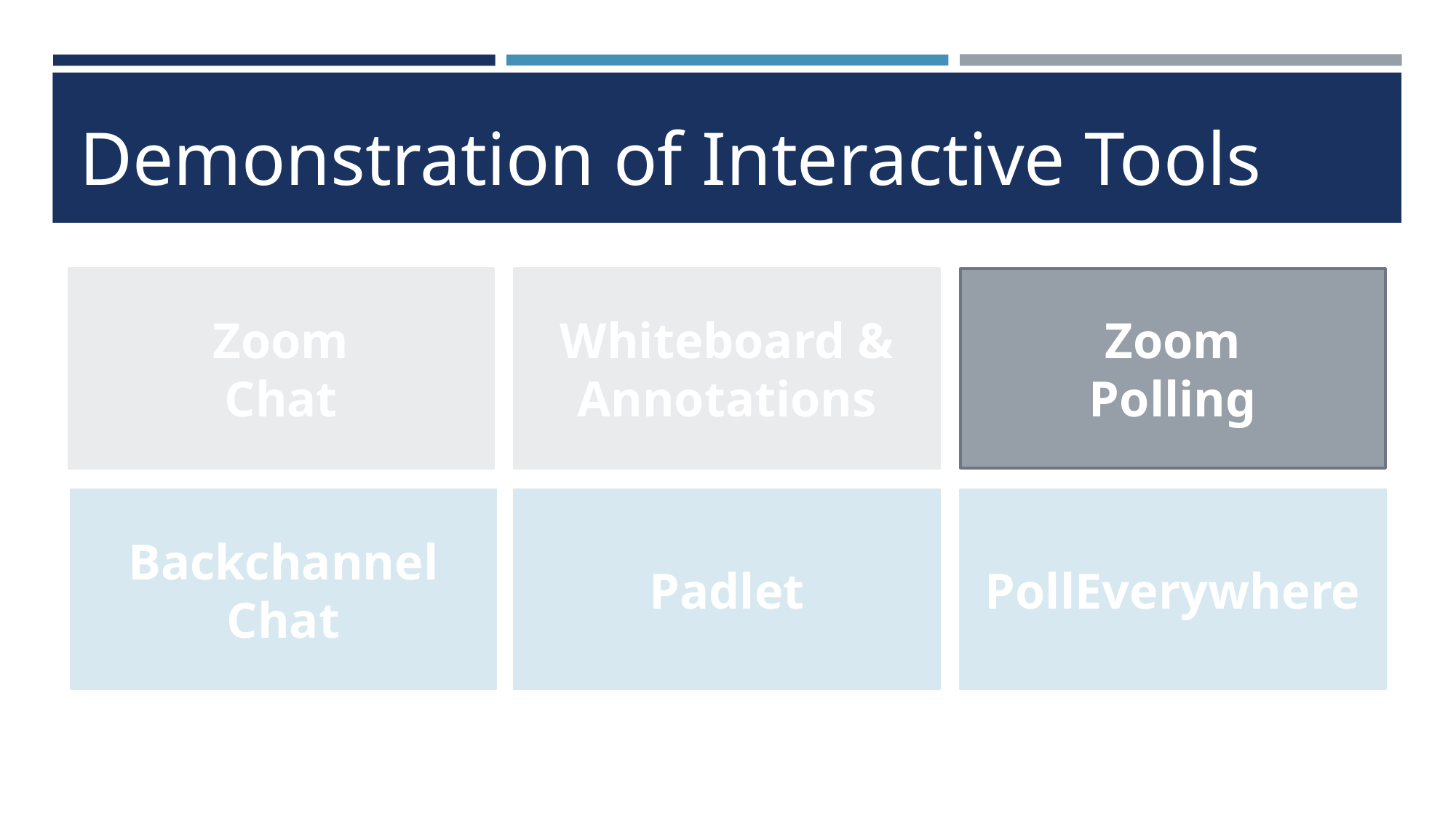

# Demonstration of Interactive Tools
Zoom
Chat
Whiteboard & Annotations
Zoom
Polling
Backchannel
Chat
Padlet
PollEverywhere

## Slide 35
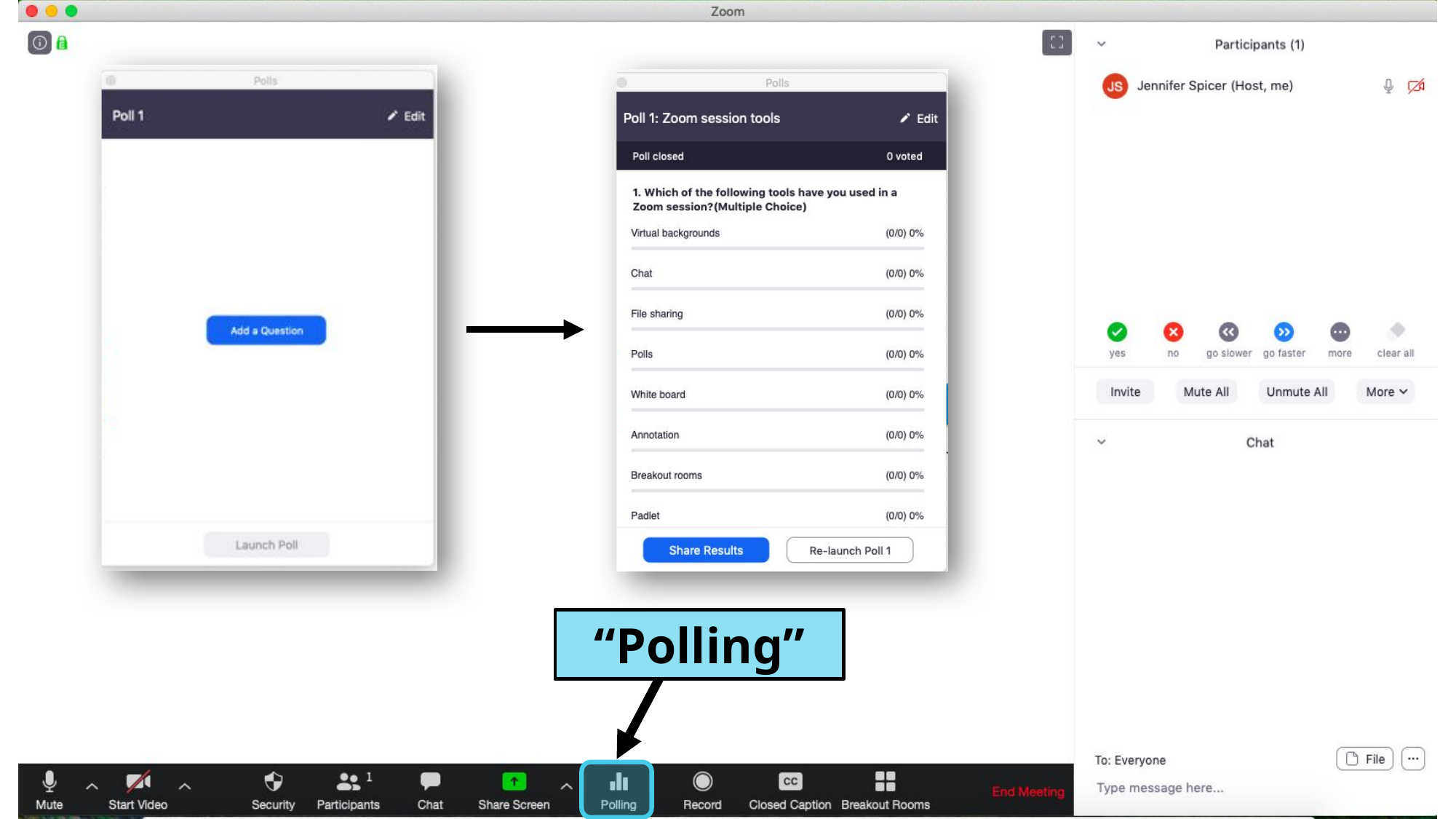

“Polling”

## Slide 36
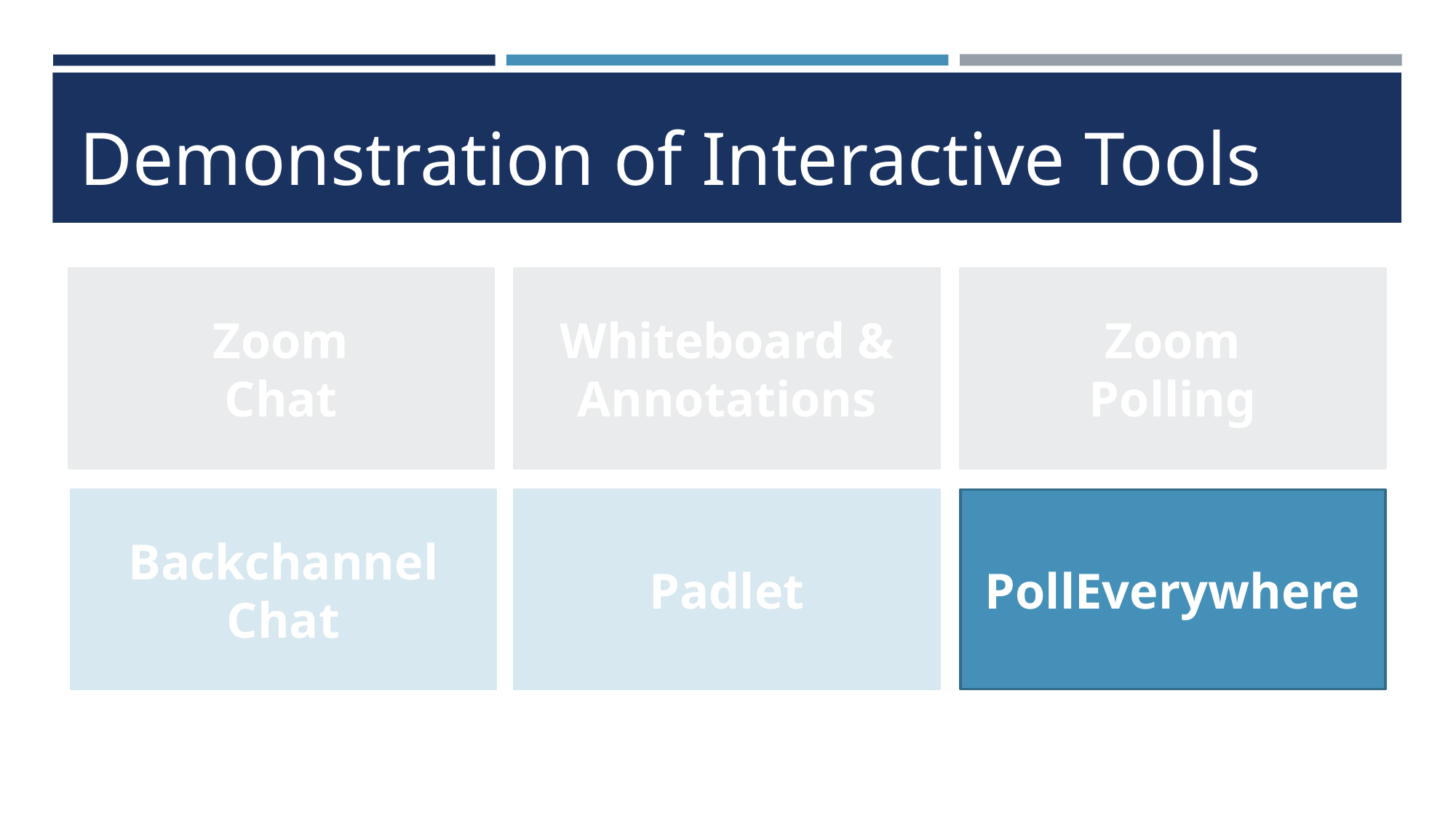

# Demonstration of Interactive Tools
Zoom
Chat
Whiteboard & Annotations
Zoom
Polling
Backchannel
Chat
Padlet
PollEverywhere

## Slide 37
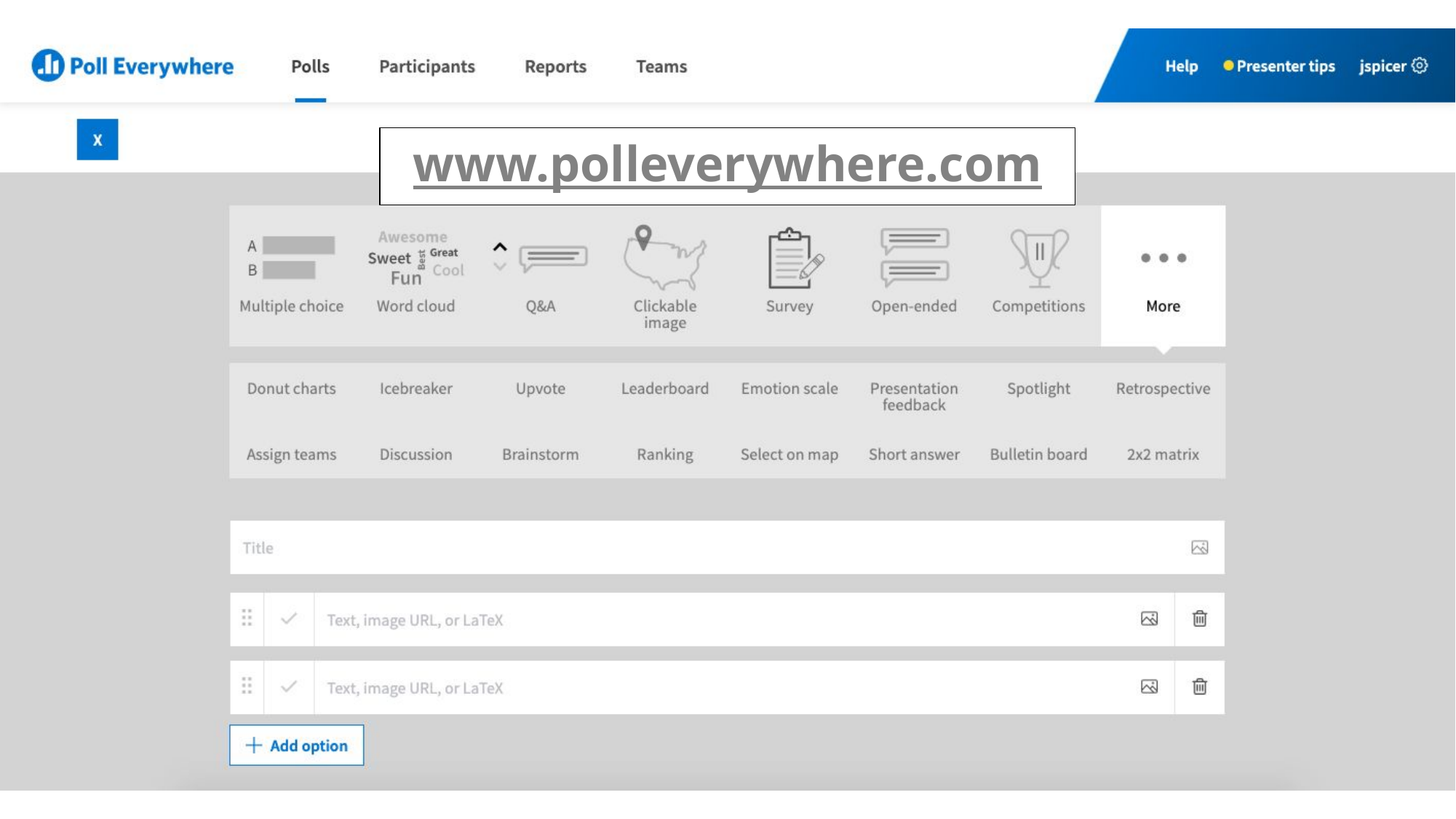

www.polleverywhere.com

## Slide 38
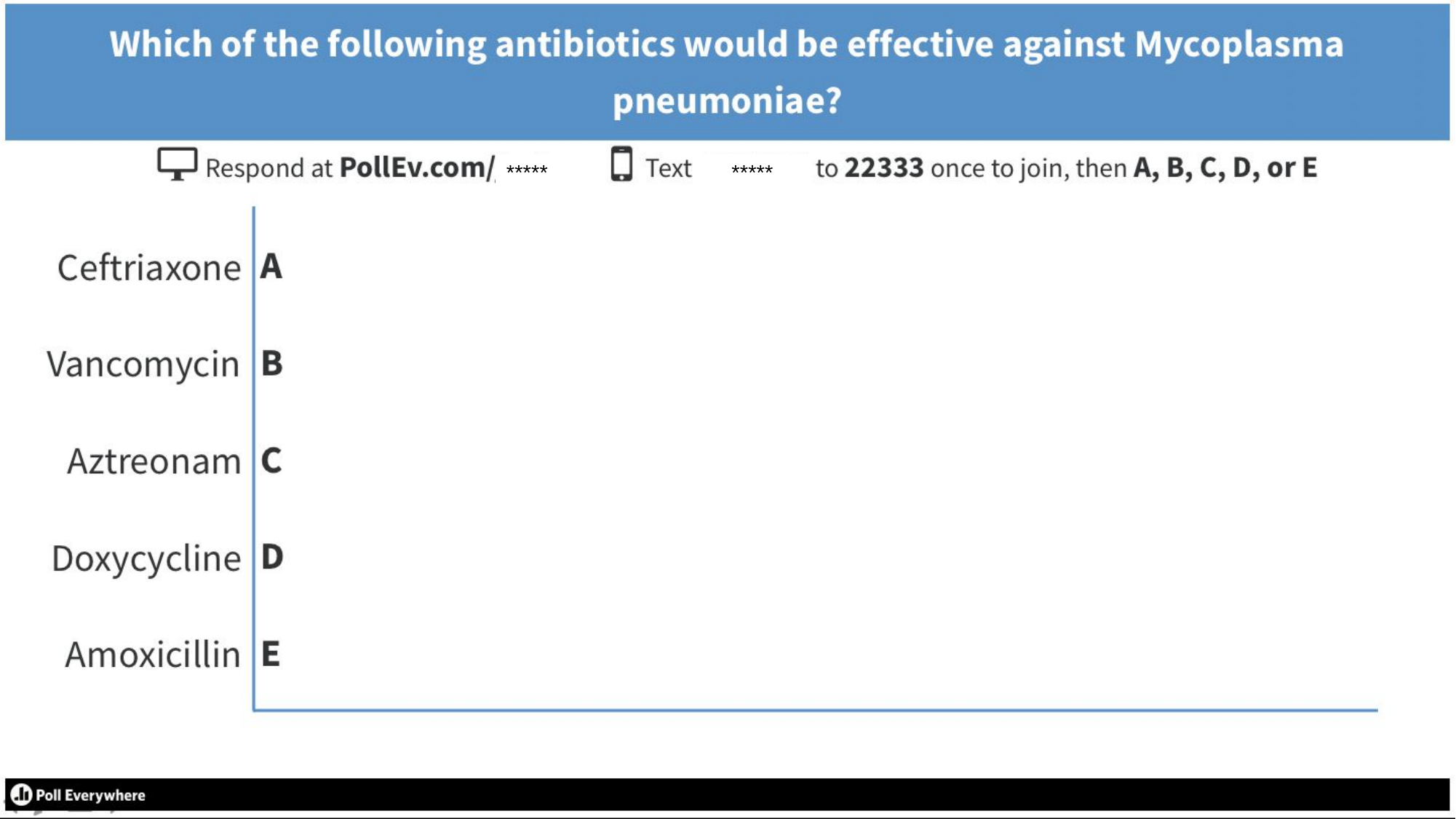

*****
*****

## Slide 39
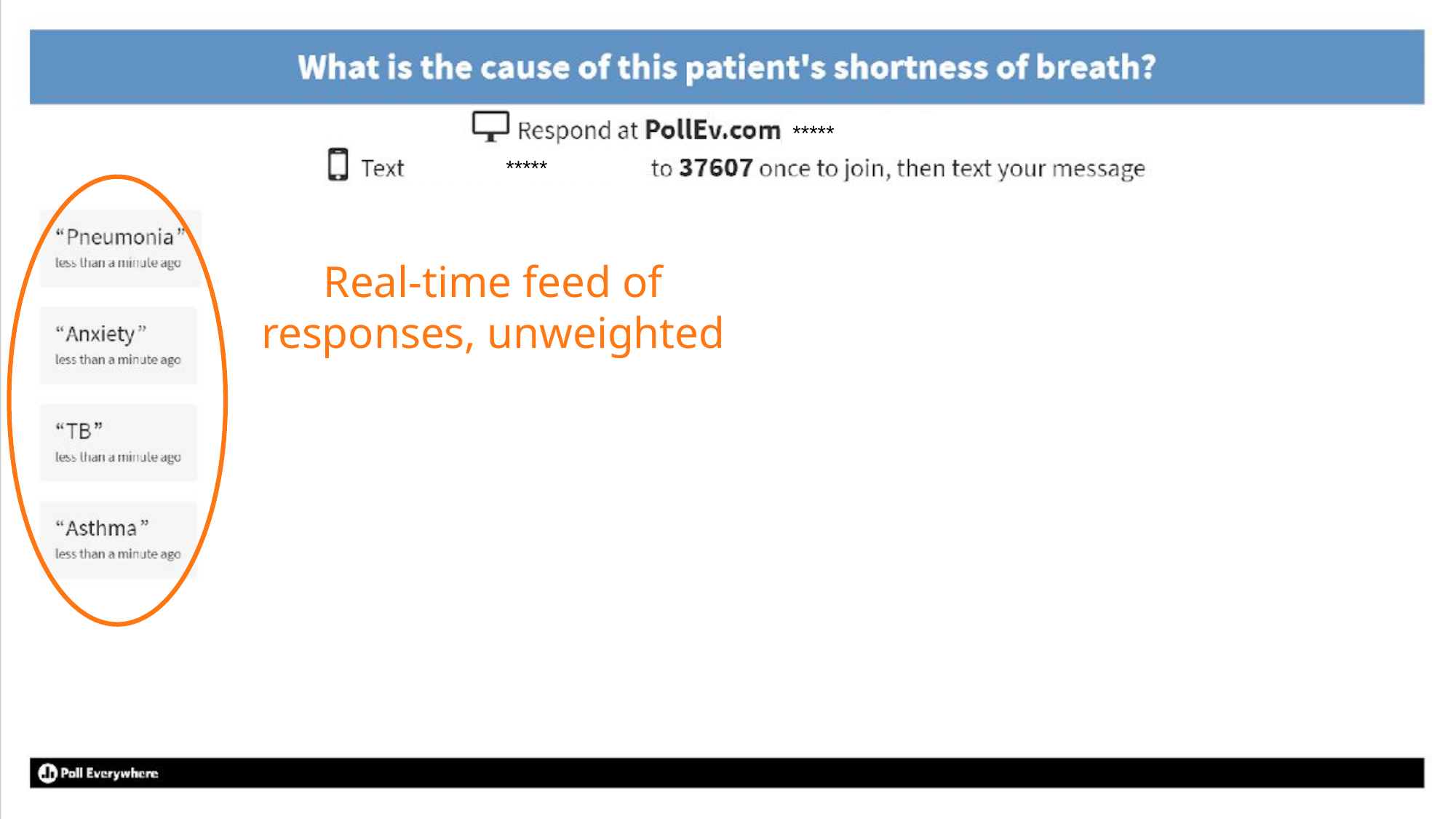

*****
*****
Real-time feed of responses, unweighted

## Slide 40
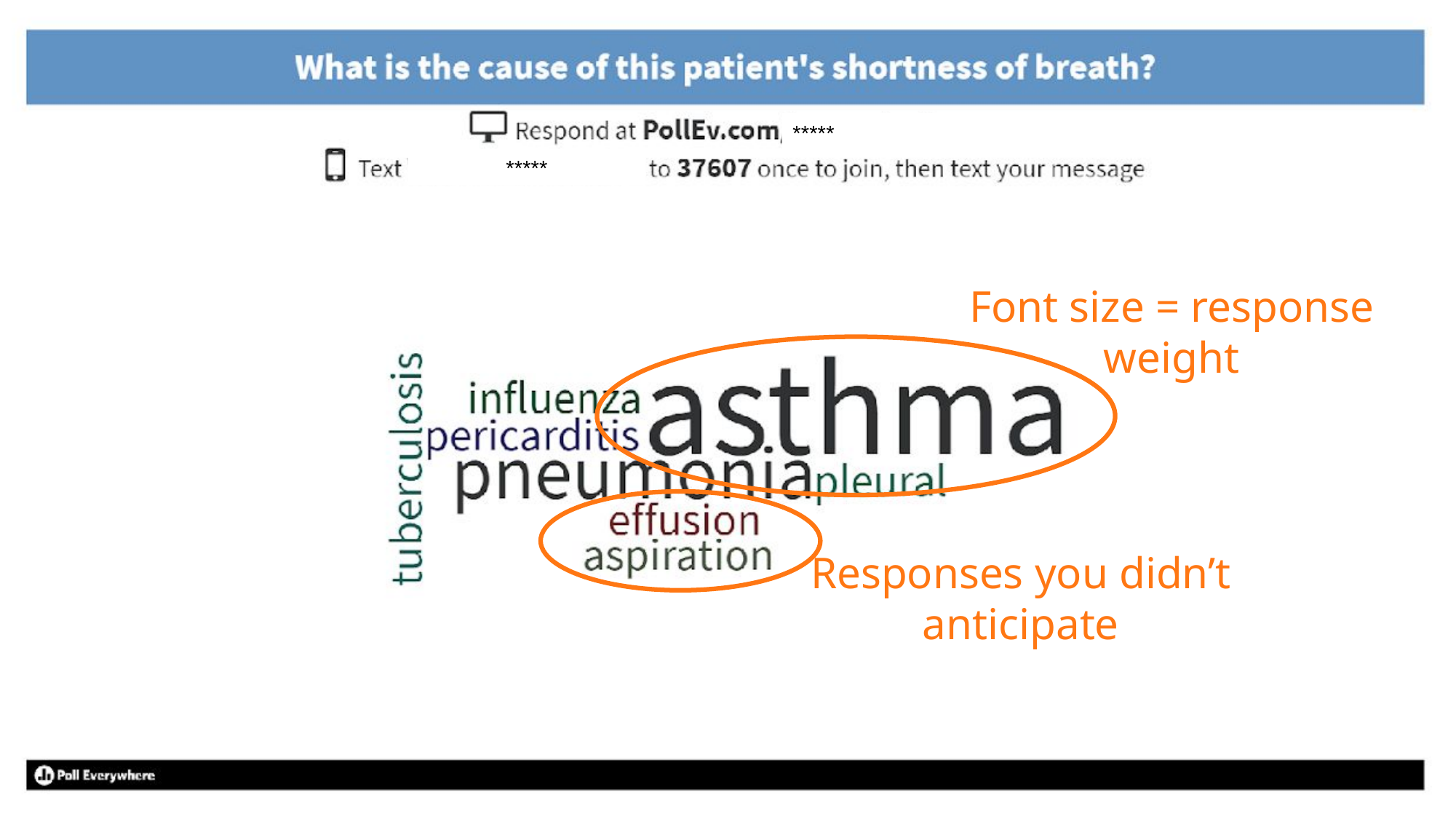

*****
*****
Font size = response weight
Responses you didn’t anticipate

## Slide 41
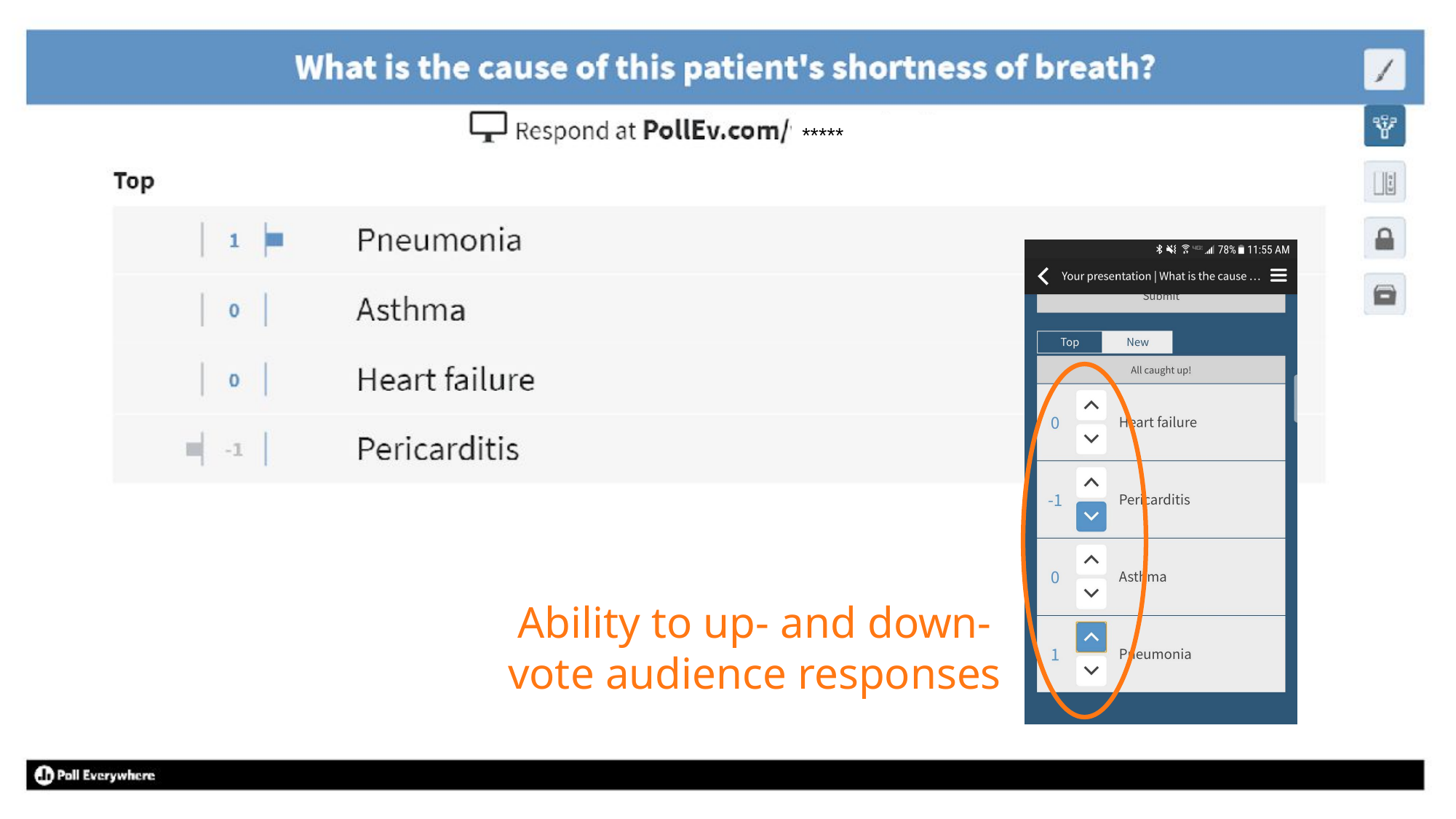

*****
Ability to up- and down-vote audience responses

## Slide 42
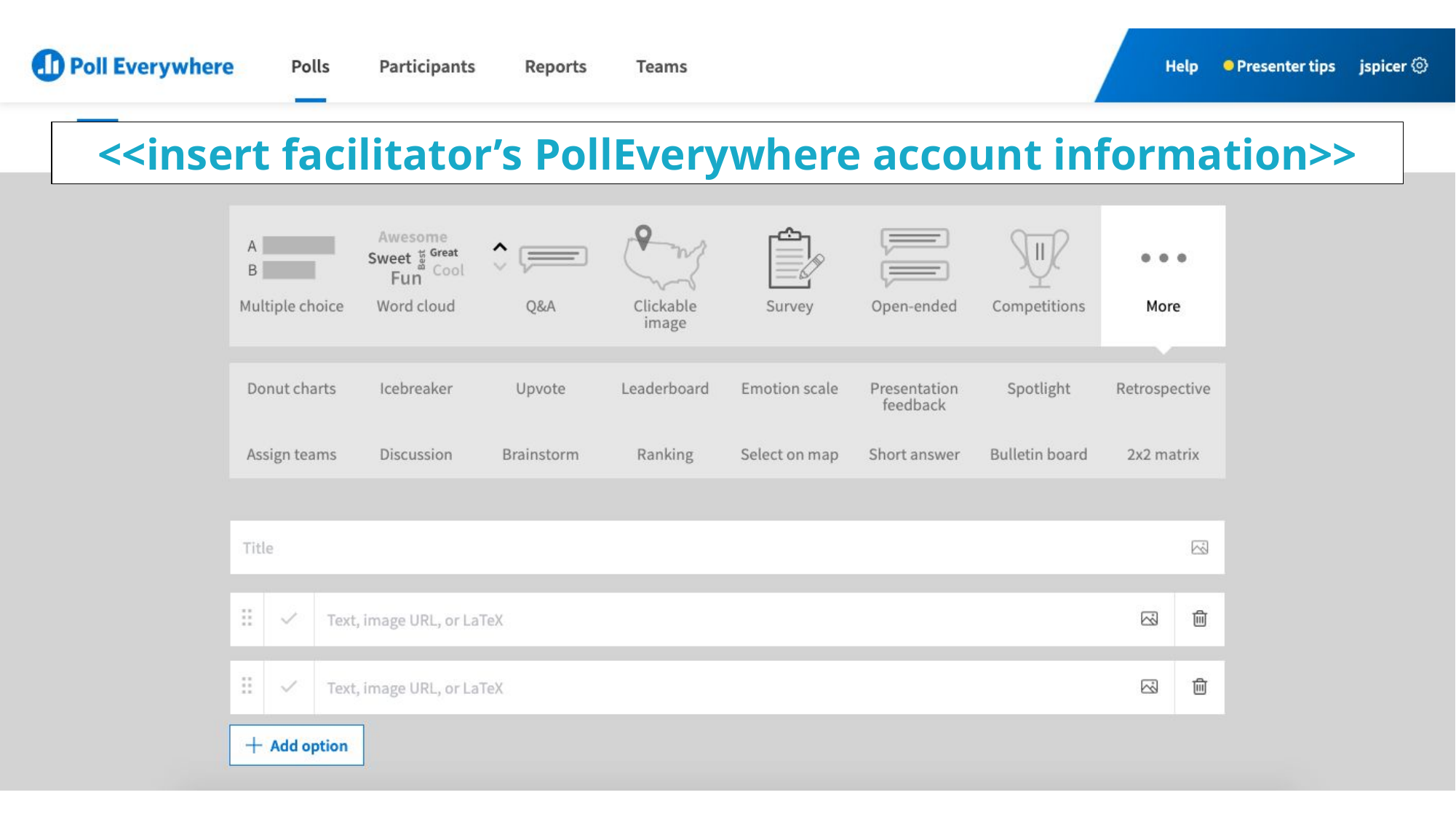

<<insert facilitator’s PollEverywhere account information>>

## Slide 43
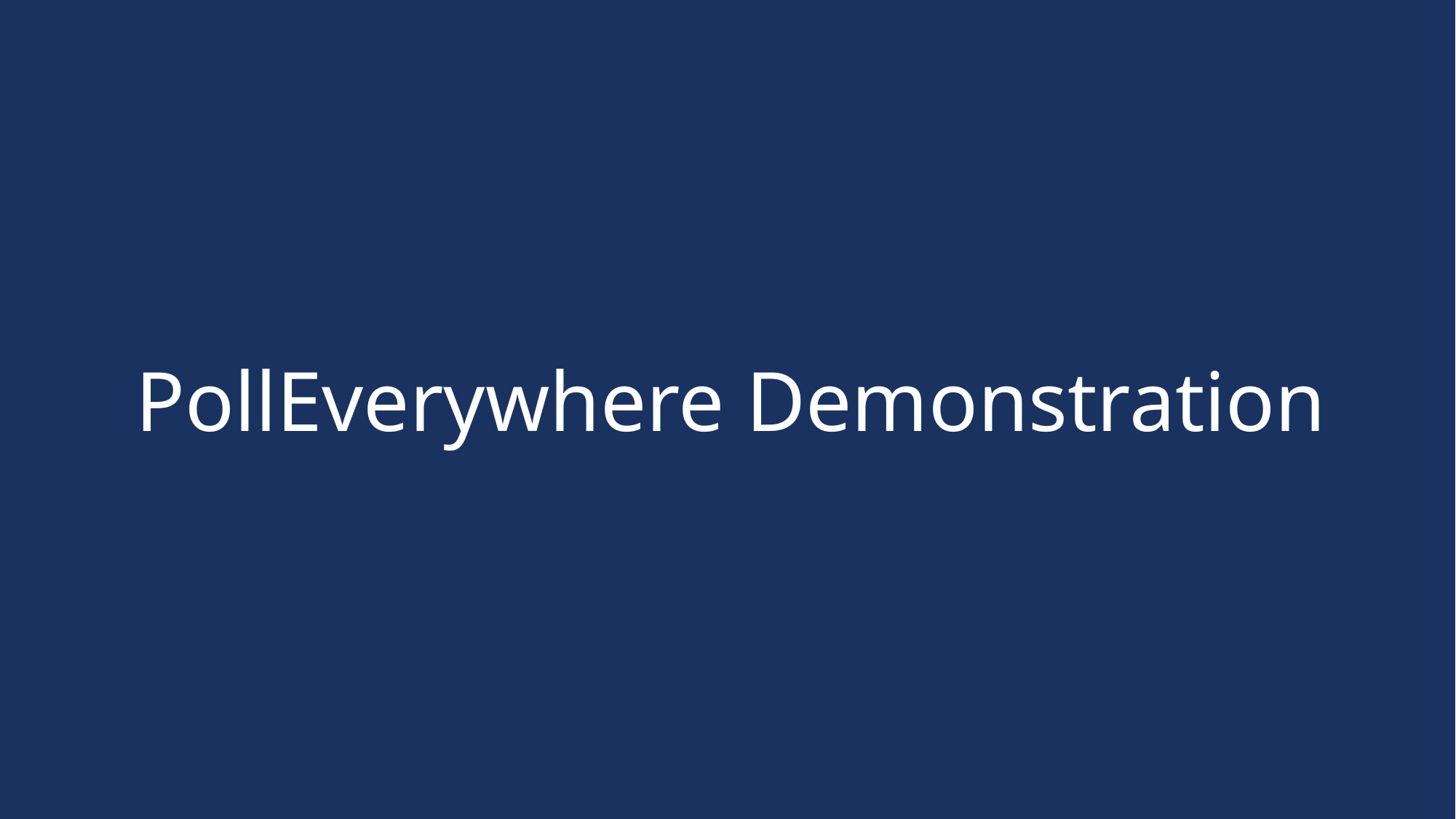

# PollEverywhere Demonstration

## Slide 44
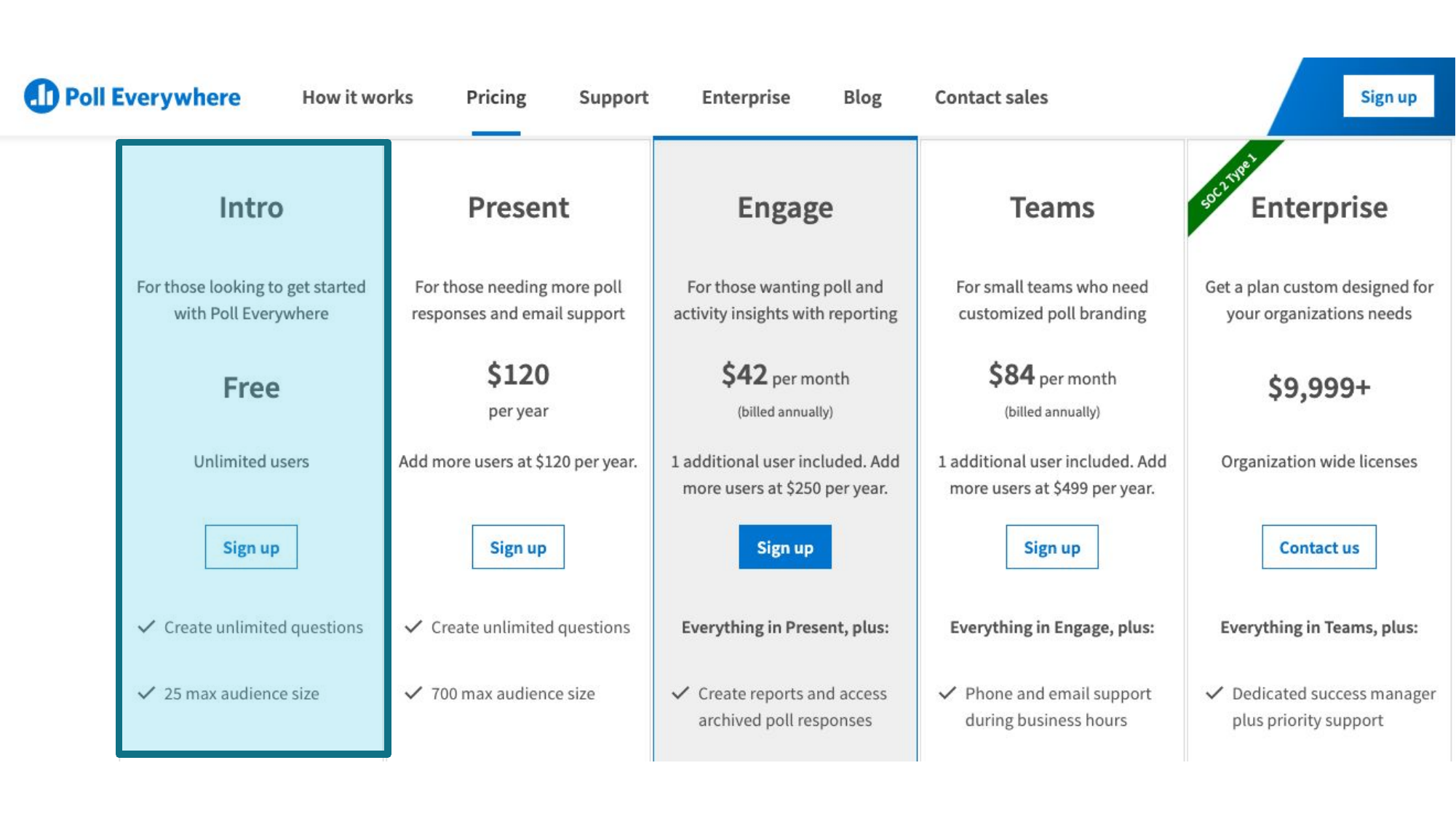

## Slide 45
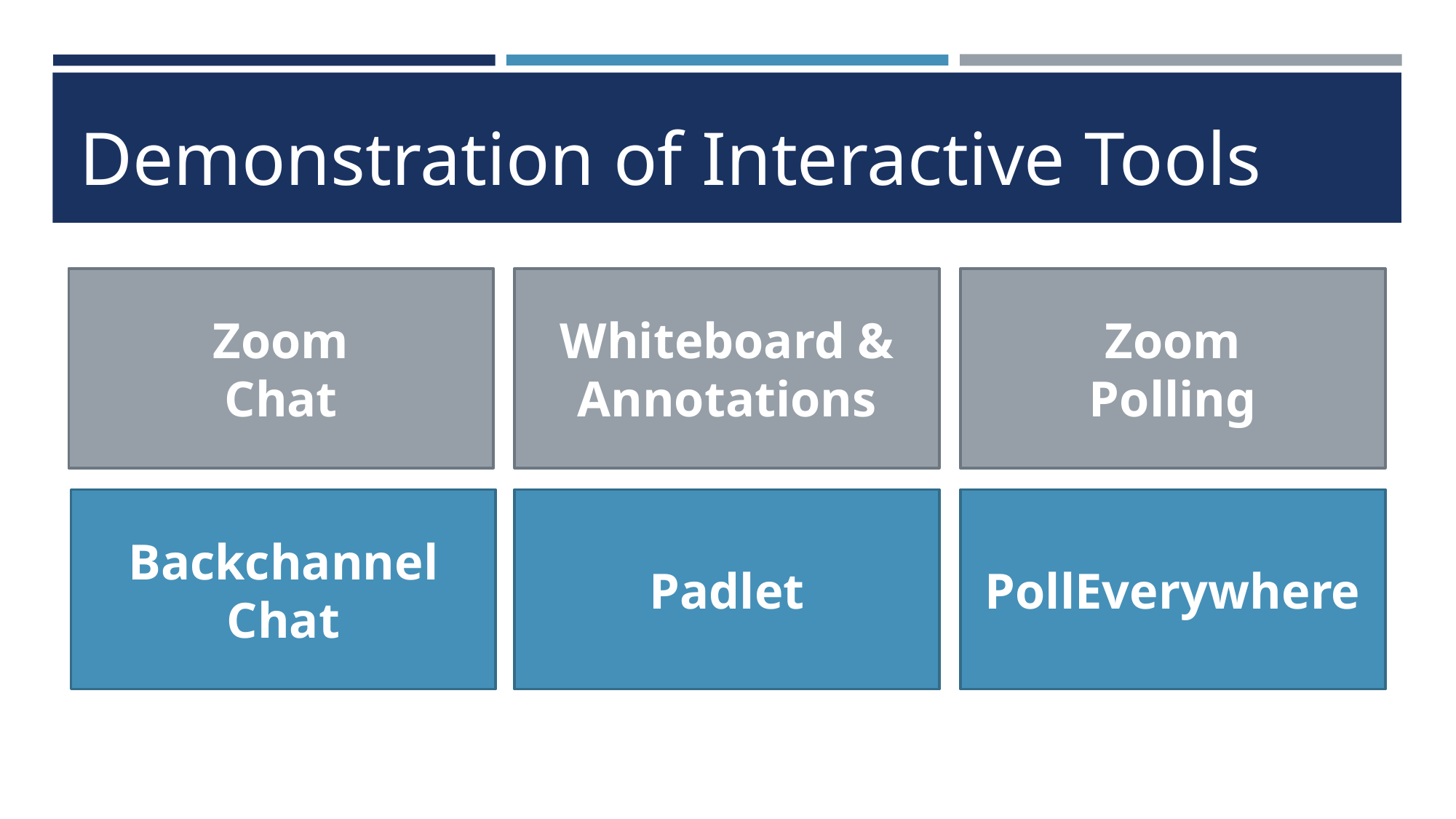

# Demonstration of Interactive Tools
Zoom
Chat
Whiteboard & Annotations
Zoom
Polling
Backchannel
Chat
Padlet
PollEverywhere

## Slide 46
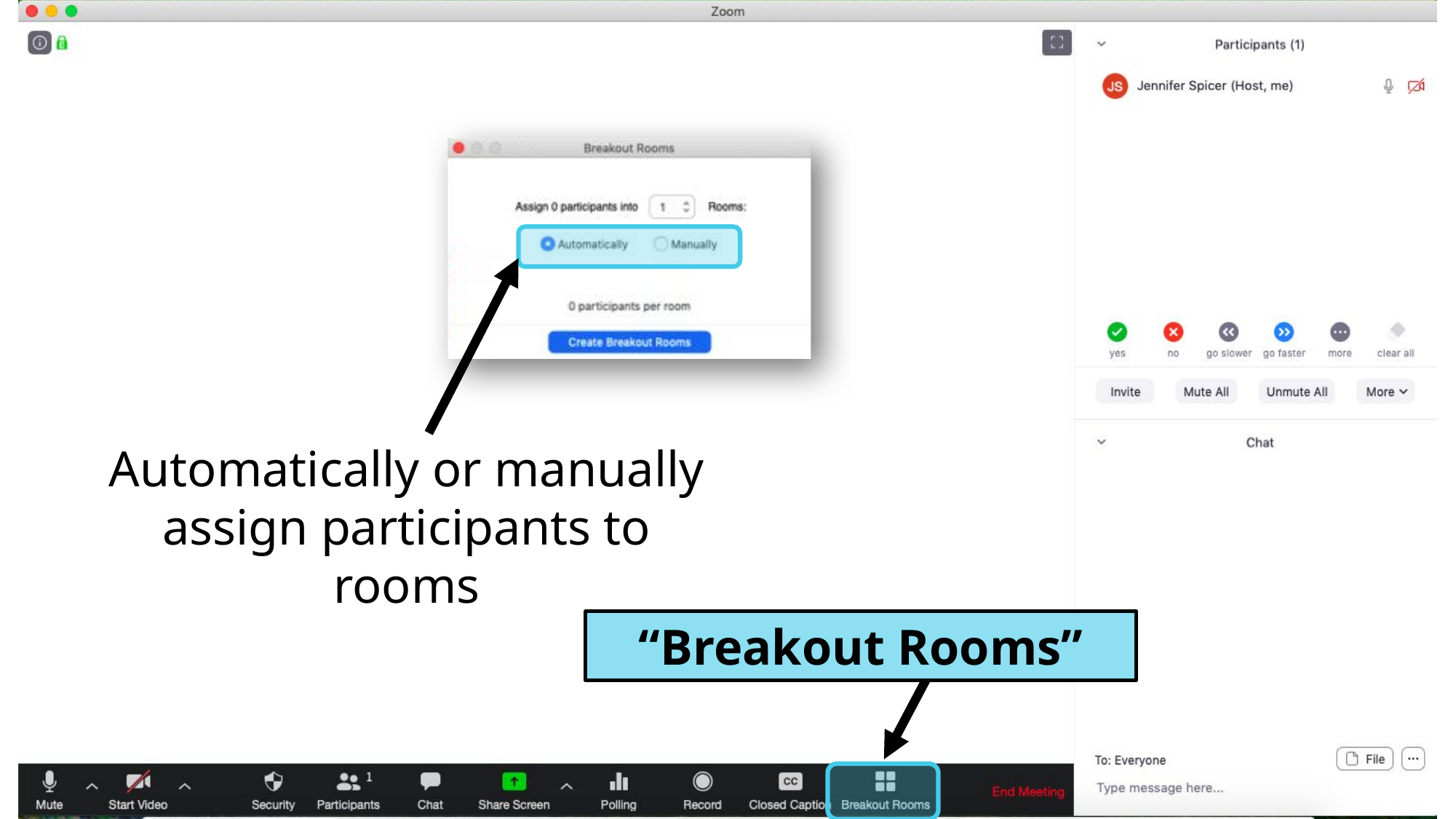

Automatically or manually assign participants to rooms
“Breakout Rooms”

## Slide 47
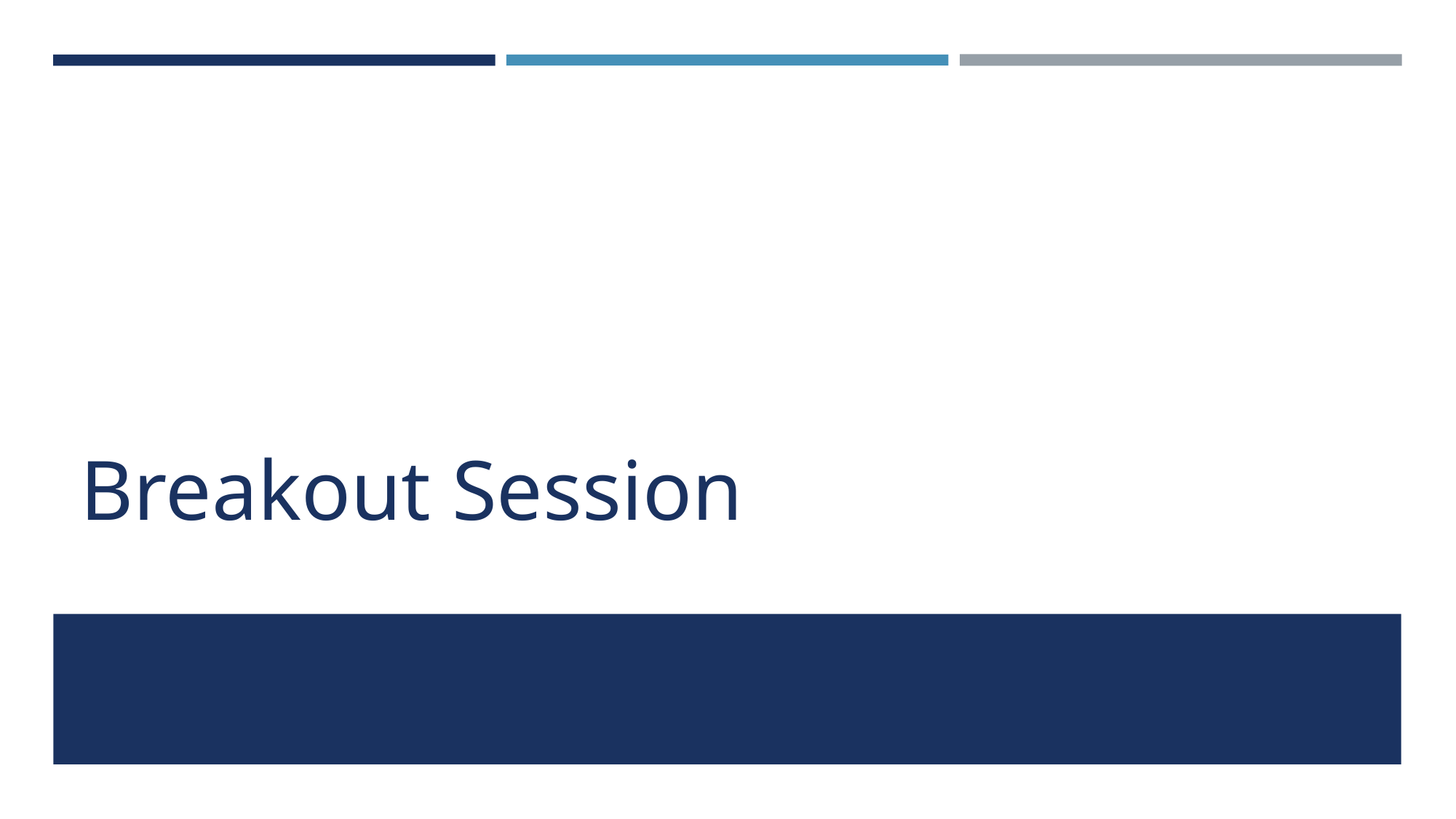

# Breakout Session

## Slide 48
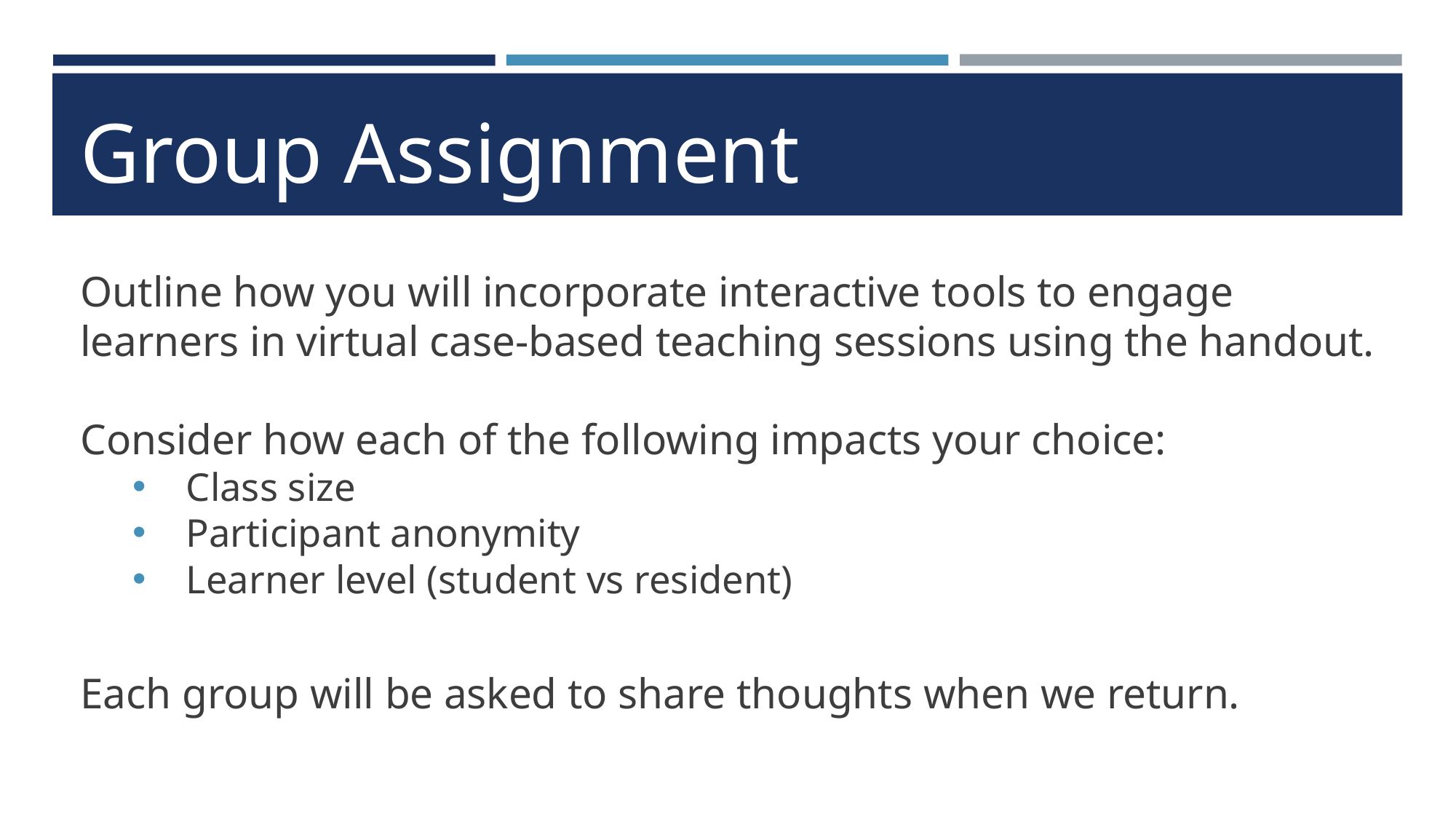

# Group Assignment
Outline how you will incorporate interactive tools to engage learners in virtual case-based teaching sessions using the handout.
Consider how each of the following impacts your choice:
Class size
Participant anonymity
Learner level (student vs resident)
Each group will be asked to share thoughts when we return.

## Slide 49
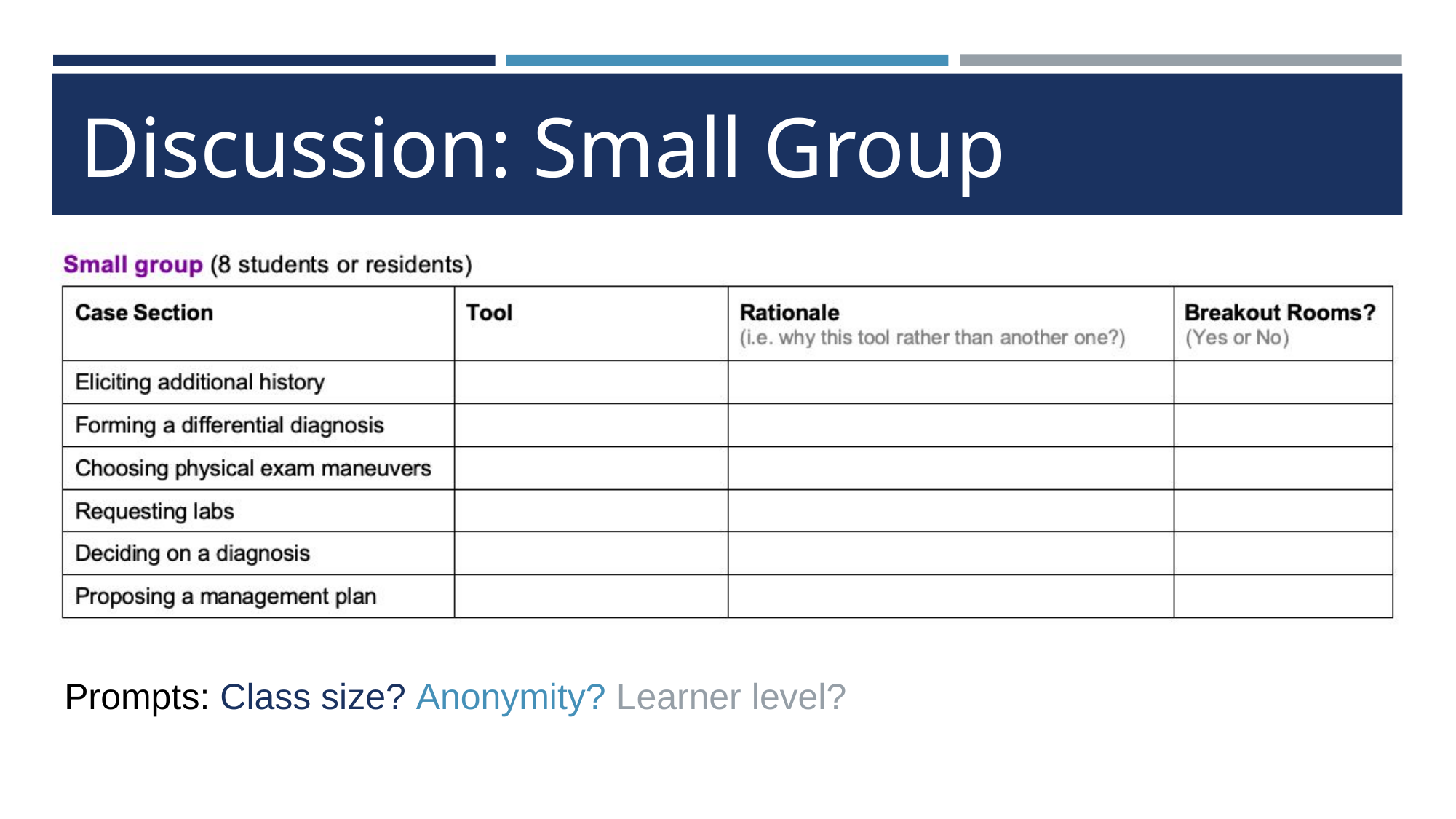

# Discussion: Small Group
Prompts: Class size? Anonymity? Learner level?

## Slide 50
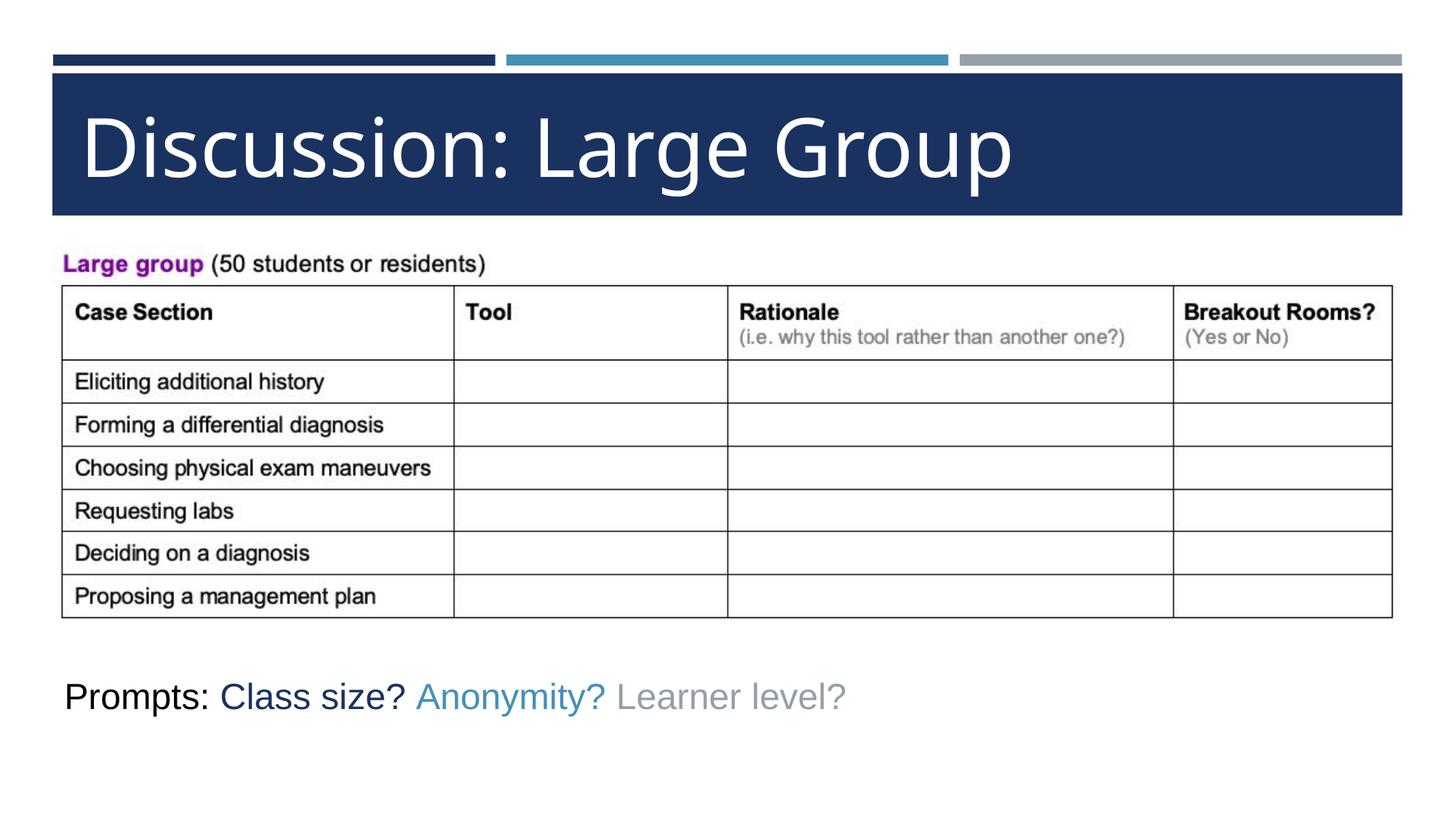

# Discussion: Large Group
Prompts: Class size? Anonymity? Learner level?

## Slide 51
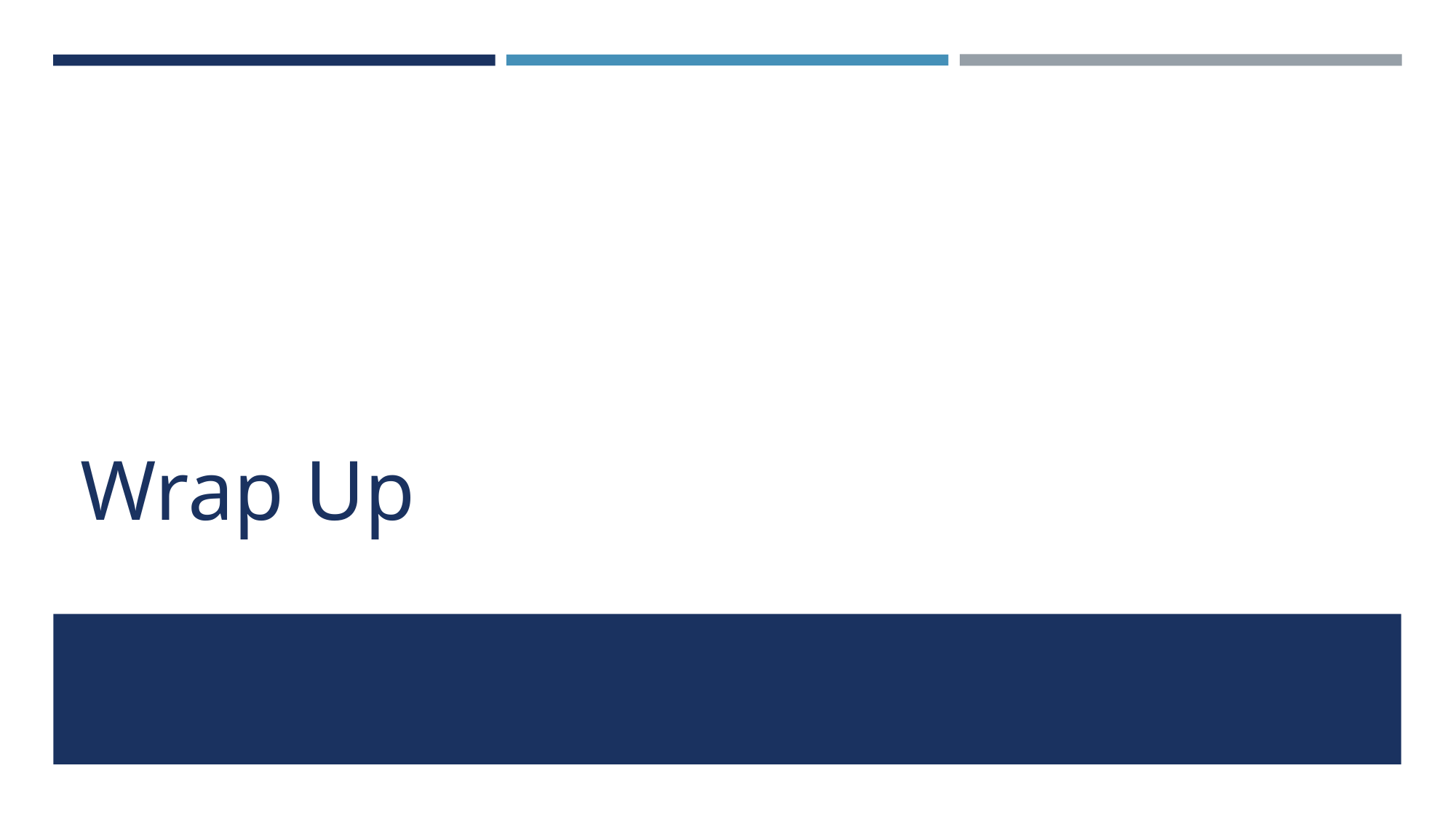

# Wrap Up

## Slide 52
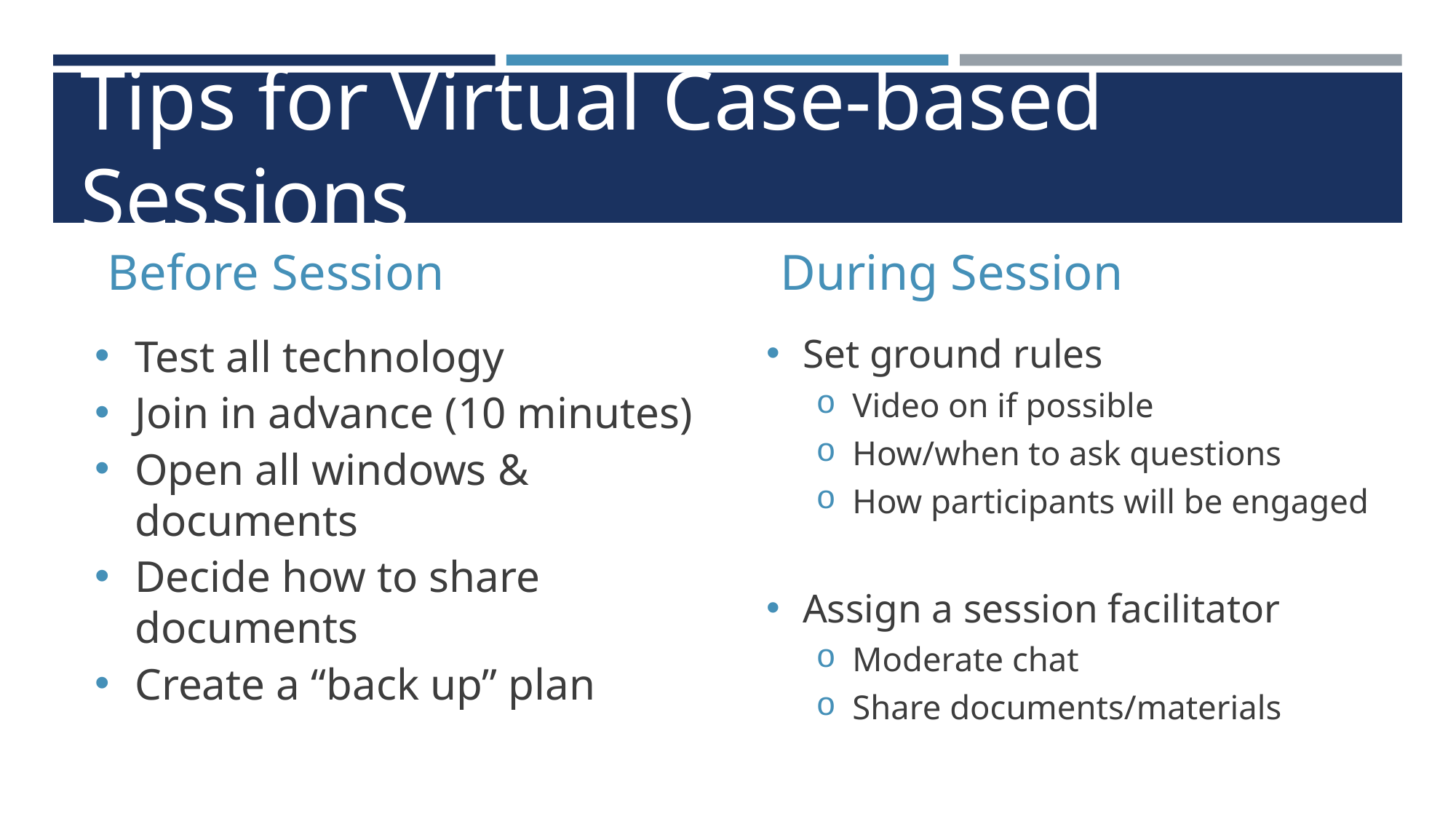

# Tips for Virtual Case-based Sessions
Before Session
During Session
Test all technology
Join in advance (10 minutes)
Open all windows & documents
Decide how to share documents
Create a “back up” plan
Set ground rules
Video on if possible
How/when to ask questions
How participants will be engaged
Assign a session facilitator
Moderate chat
Share documents/materials

## Slide 53
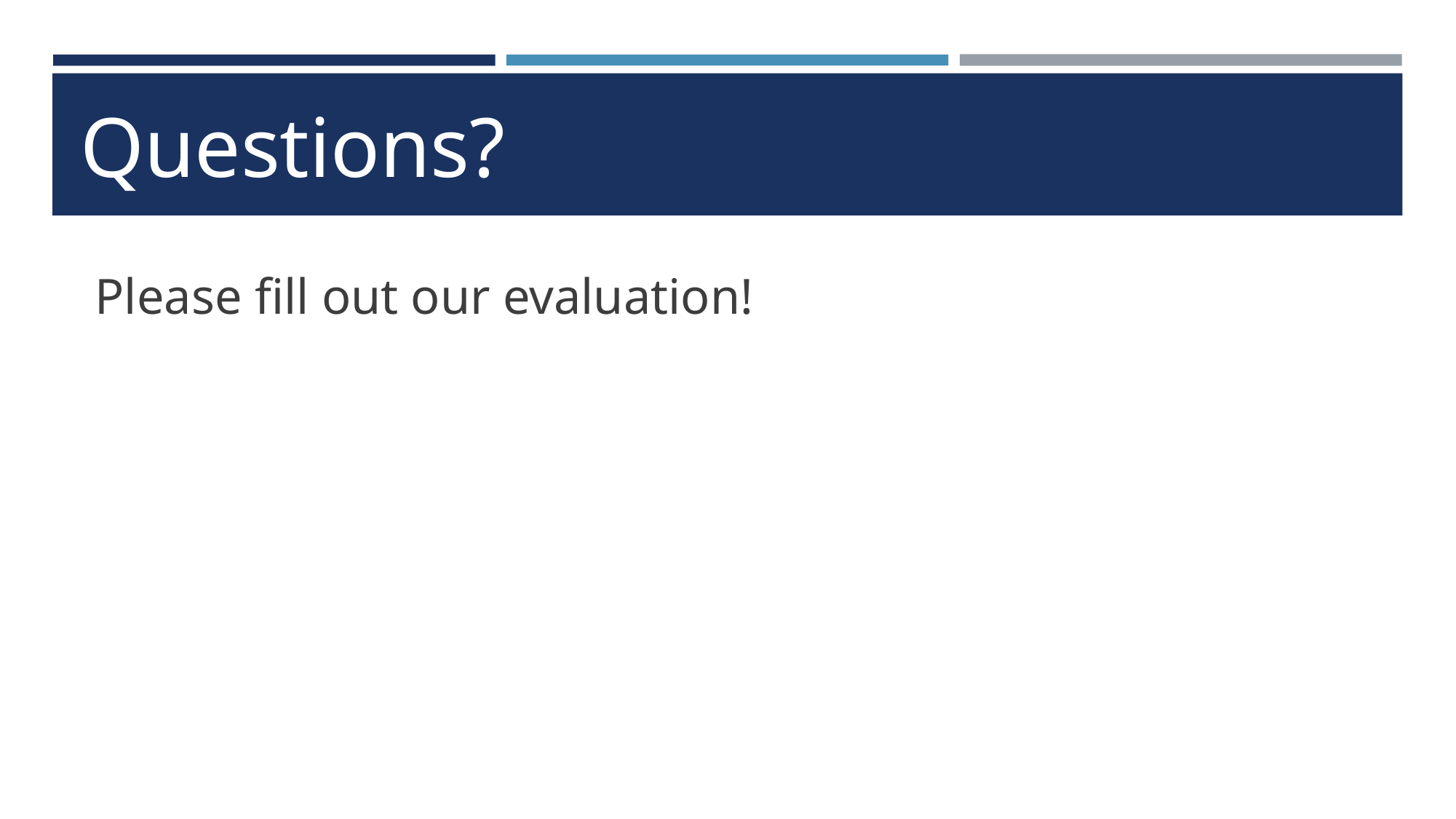

# Questions?
Please fill out our evaluation!

## Slide 54
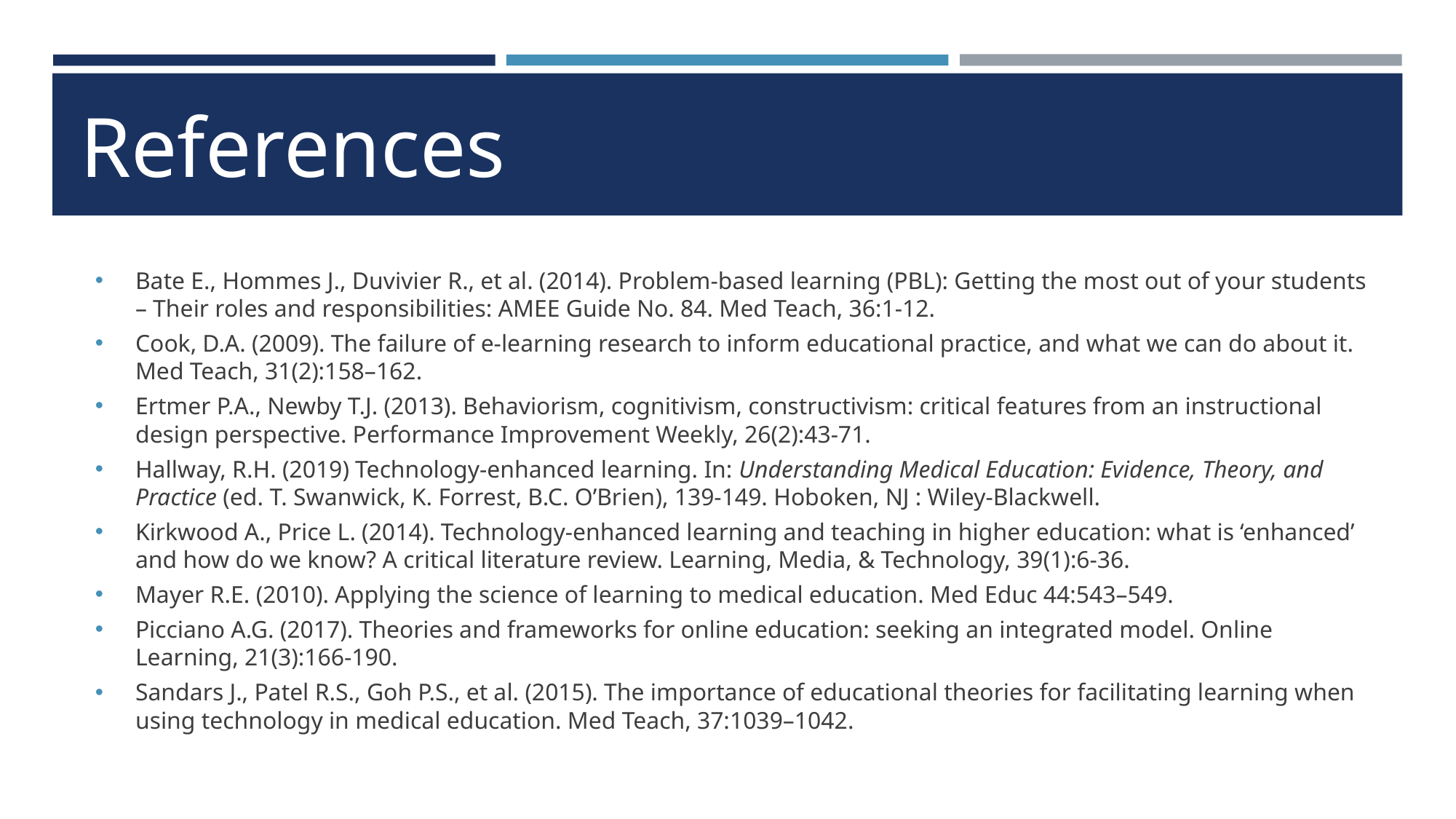

# References
Bate E., Hommes J., Duvivier R., et al. (2014). Problem-based learning (PBL): Getting the most out of your students – Their roles and responsibilities: AMEE Guide No. 84. Med Teach, 36:1-12.
Cook, D.A. (2009). The failure of e-learning research to inform educational practice, and what we can do about it. Med Teach, 31(2):158–162.
Ertmer P.A., Newby T.J. (2013). Behaviorism, cognitivism, constructivism: critical features from an instructional design perspective. Performance Improvement Weekly, 26(2):43-71.
Hallway, R.H. (2019) Technology-enhanced learning. In: Understanding Medical Education: Evidence, Theory, and Practice (ed. T. Swanwick, K. Forrest, B.C. O’Brien), 139-149. Hoboken, NJ : Wiley-Blackwell.
Kirkwood A., Price L. (2014). Technology-enhanced learning and teaching in higher education: what is ‘enhanced’ and how do we know? A critical literature review. Learning, Media, & Technology, 39(1):6-36.
Mayer R.E. (2010). Applying the science of learning to medical education. Med Educ 44:543–549.
Picciano A.G. (2017). Theories and frameworks for online education: seeking an integrated model. Online Learning, 21(3):166-190.
Sandars J., Patel R.S., Goh P.S., et al. (2015). The importance of educational theories for facilitating learning when using technology in medical education. Med Teach, 37:1039–1042.
