# Supplementary material for: A Faculty Development Workshop for Planning and Implementing Interactive Virtual Case-Based Teaching
Source: MedEdPORTAL. 2021 Mar 17;17:11126. doi: 10.15766/mep_2374-8265.11126 (PMC7970636; doi:10.15766/mep_2374-8265.11126)

**Appendix D: Facilitator Guide for Tech Demonstrations**

This guide provides a step-by-step guide with screenshots to help the facilitator set up each tech tool and demonstrate it during the session.

BackChannel Chat Set Up and Demonstration

Prior to the session, the facilitator does not need to do anything to get Backchannel Chat set up. During the session, the facilitator should go to the website [http://backchannelchat.com](http://backchannelchat.com/) where they will see the screen below:


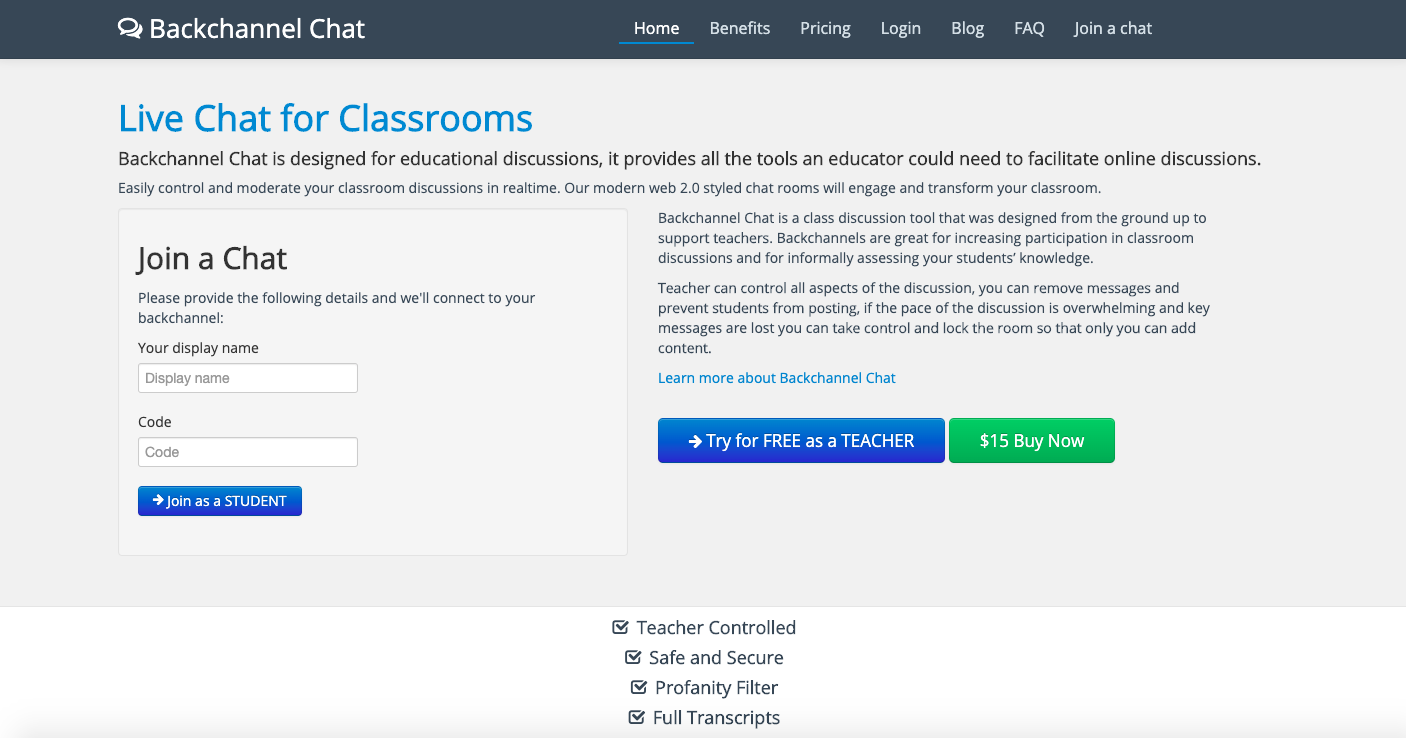


Screenshot images author owned.

The facilitator will click on the “Try for FREE as a TEACHER” button, and the screen below will pop up requesting an email address, a name to display for the teacher in the chat, and a title for the chat:


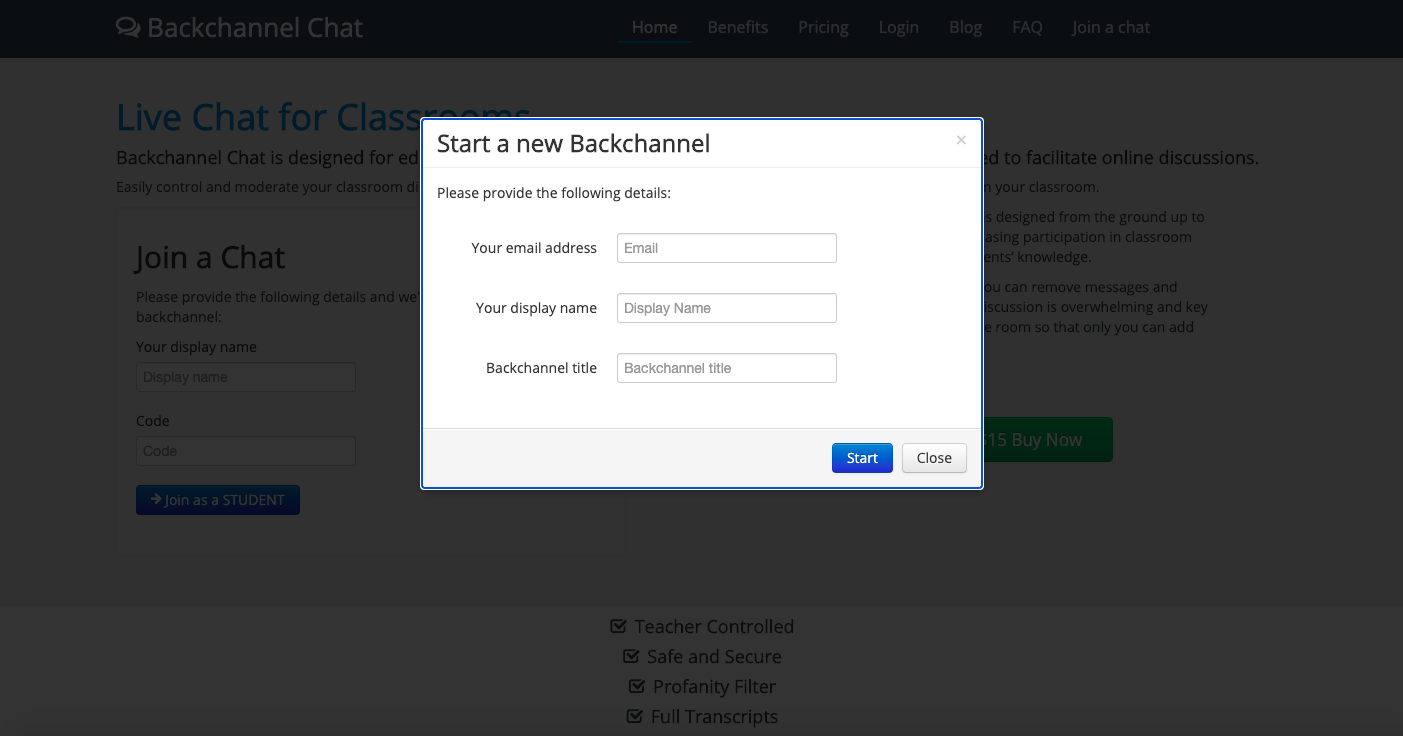


After completing that information and clicking “start,” the facilitator will be taken to a webpage that looks like the one below. The instructor can hit the blue “x” in the upper right hand corner and then type in material comments in the chat box below:


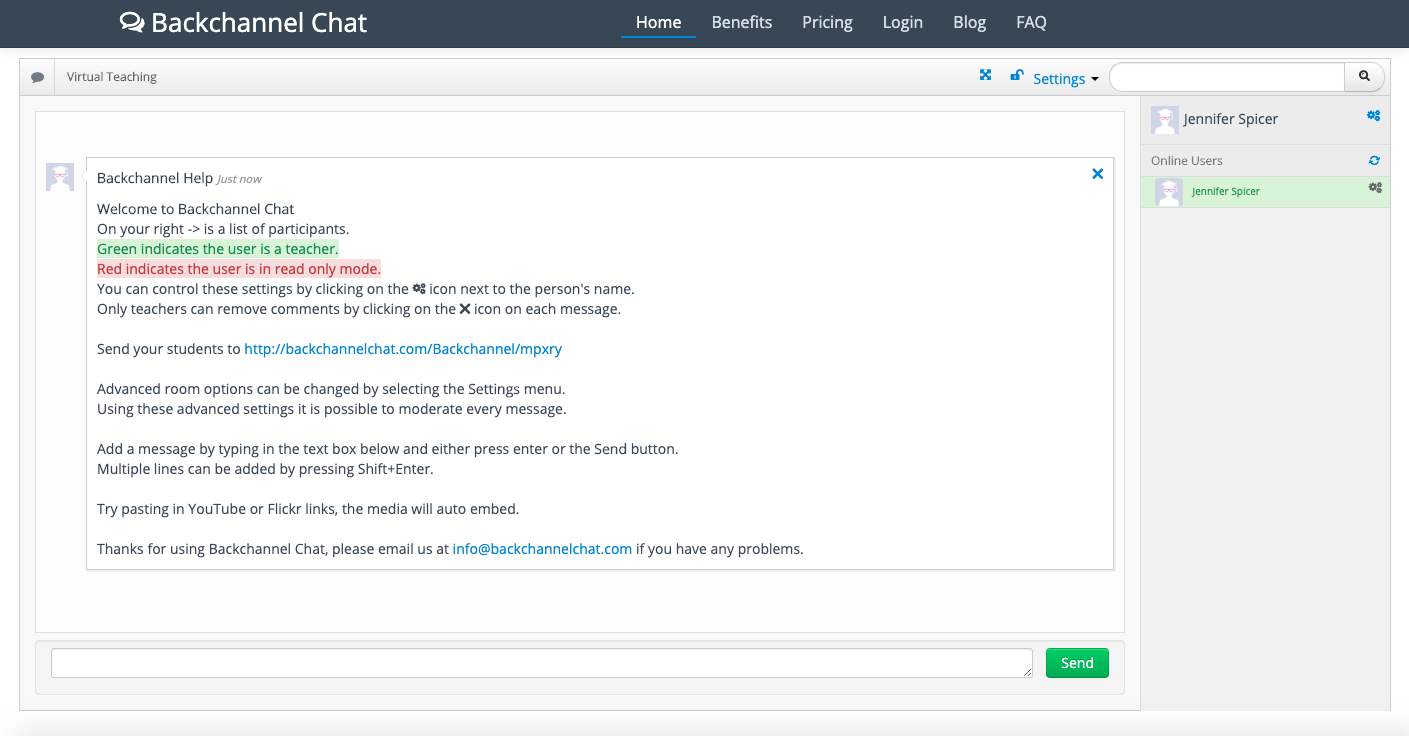


After inputting a comment, the facilitator can click on the “pin” of that chat to have it “pinned” to the top of the chat and thus visible to anyone who joins. The facilitator can also demonstration how to “upvote” by clicking on the “thumbs up” button on the chat.


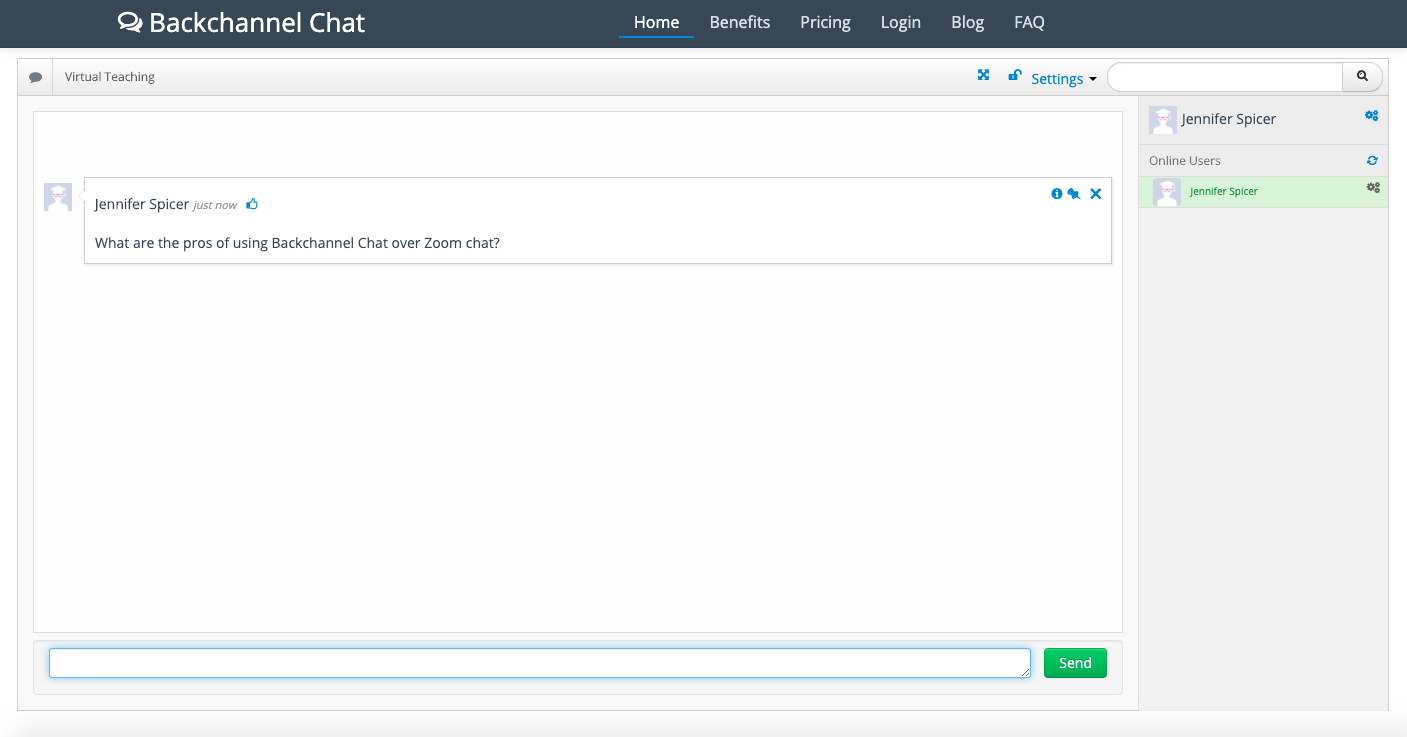


The screenshot below shows what a “pinned” chat looks like:


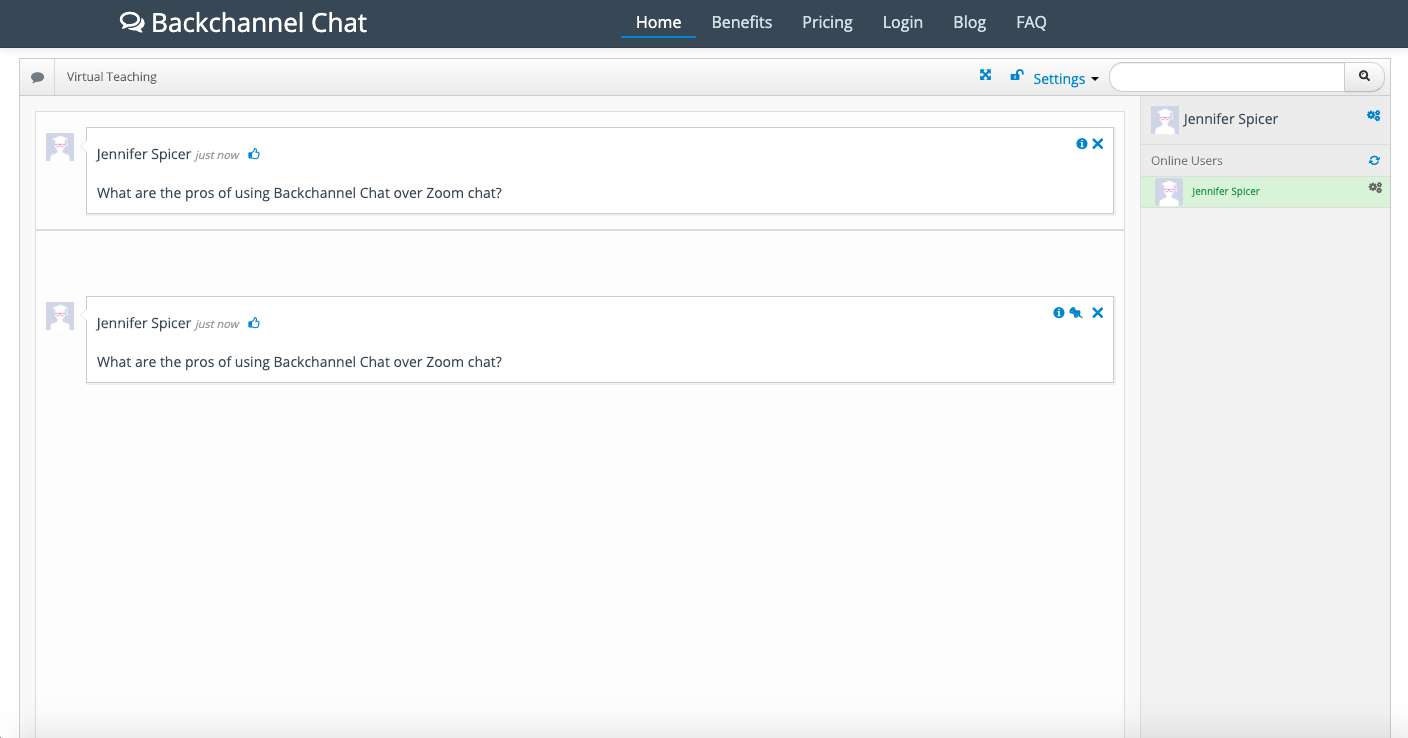


Padlet Set Up and Demonstration

Prior to the session, the facilitator should sign up for a free version of Padlet (website: <http://padlet.com>) and have the website opened to the dashboard and signed in to their account. Also, before the session, the facilitator should make two Padlets.

The first Padlet shows a possible layout for a “Case Conference” using the “Shelf” layout:


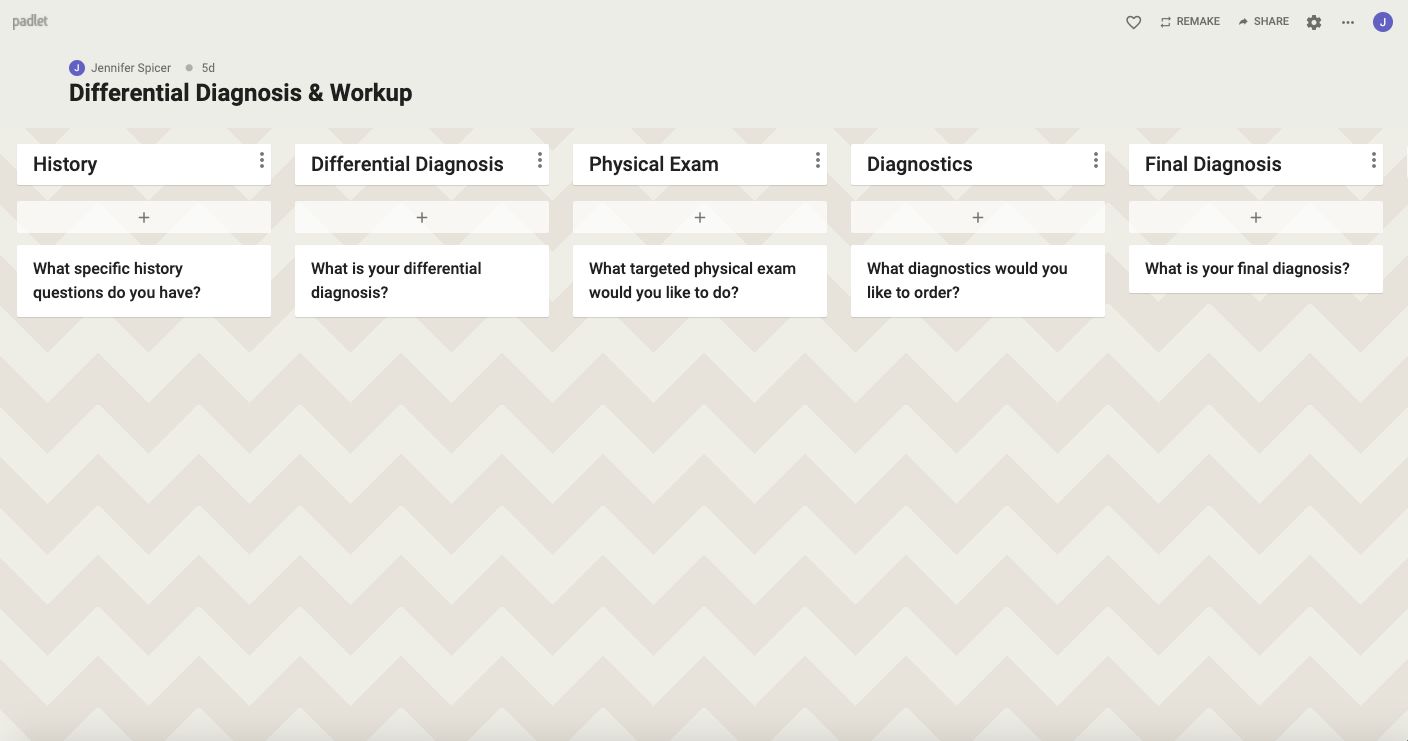


The second Padlet will be used during the session to allow the participants to practice using Padlet. The facilitator should use the “Wall” layout and write “Pros and Cons of Padlet” as the title, as shown below:


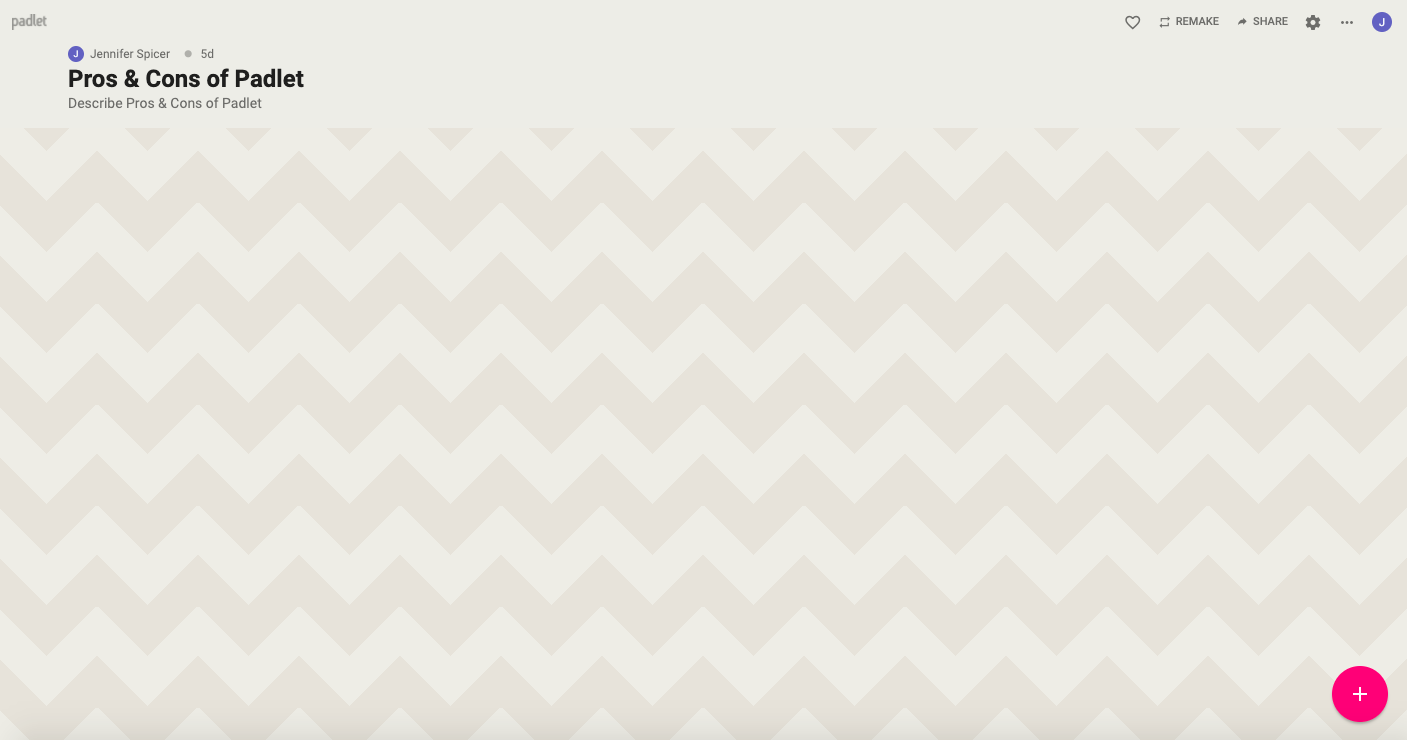


During the session, the facilitator will start on the dashboard (shown below), and the facilitator will first click on the pink “Make a Padlet” button:


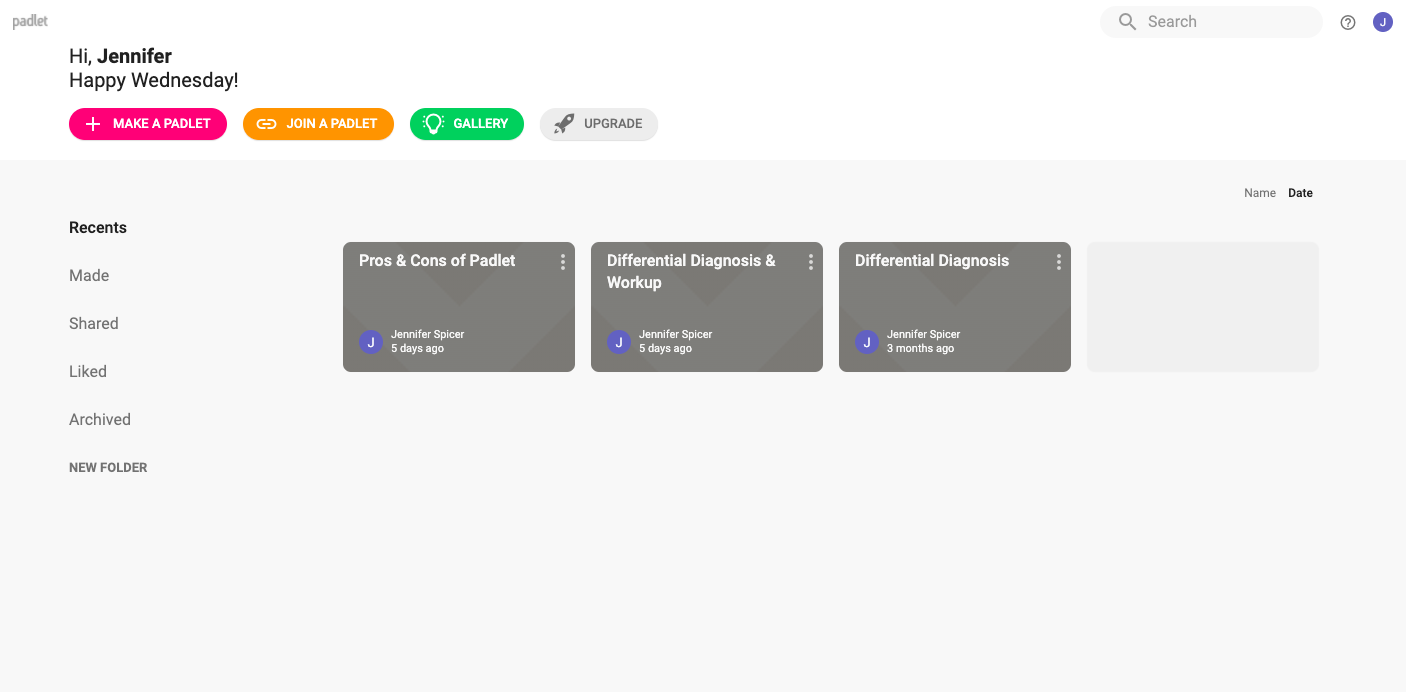


Clicking on the “Make a Padlet button will take the user to a page that shows options for different Padlet layouts, which the facilitator can describe to the participants (screenshot below):


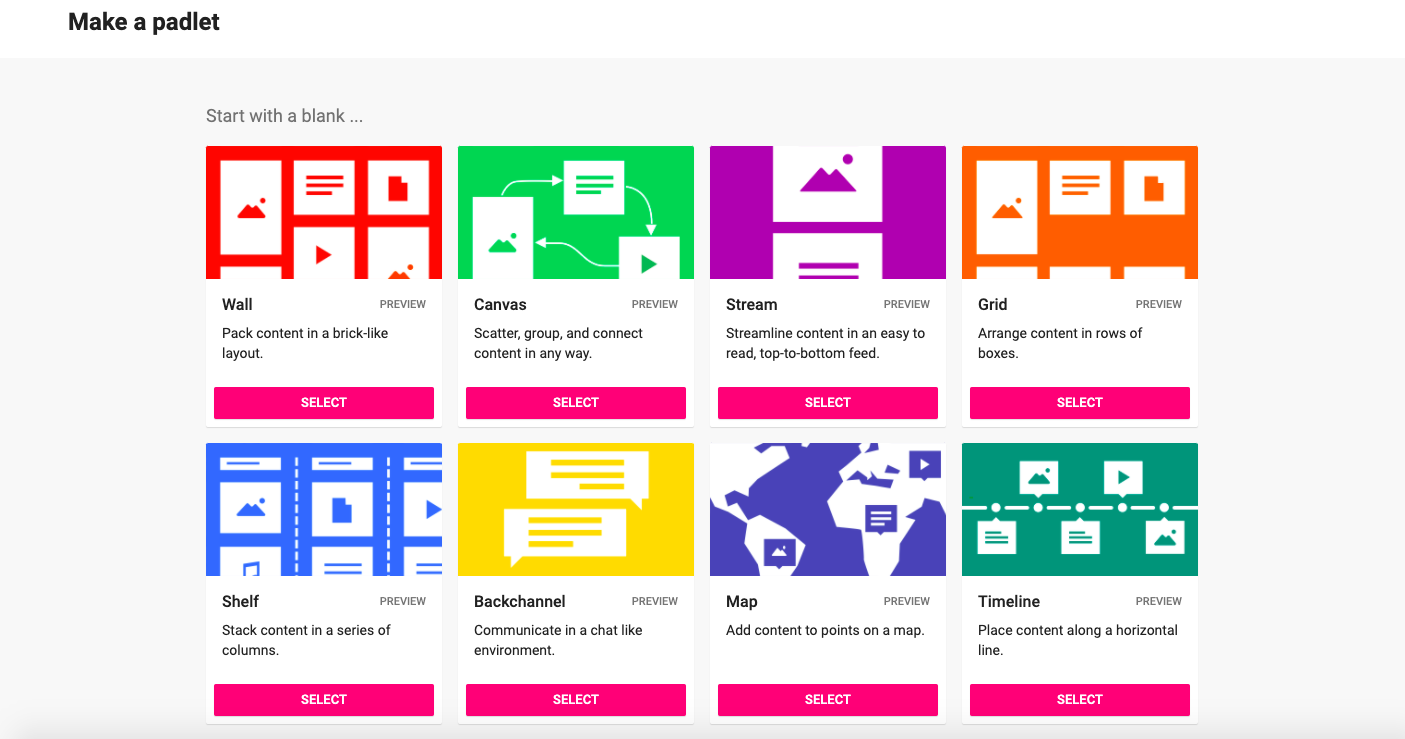


After going through those different options, the facilitator can go back to the dashboard, and select the previously created Padlets to first show the “Case Conference” layout. While on this page, the facilitator should click on the “gear” icon in the upper right corner to open up options and show the participants the various options available to customize Padlets (e.g. whether individuals’ names appear, whether individuals can comment, how individuals can “react” to posts, etc.):


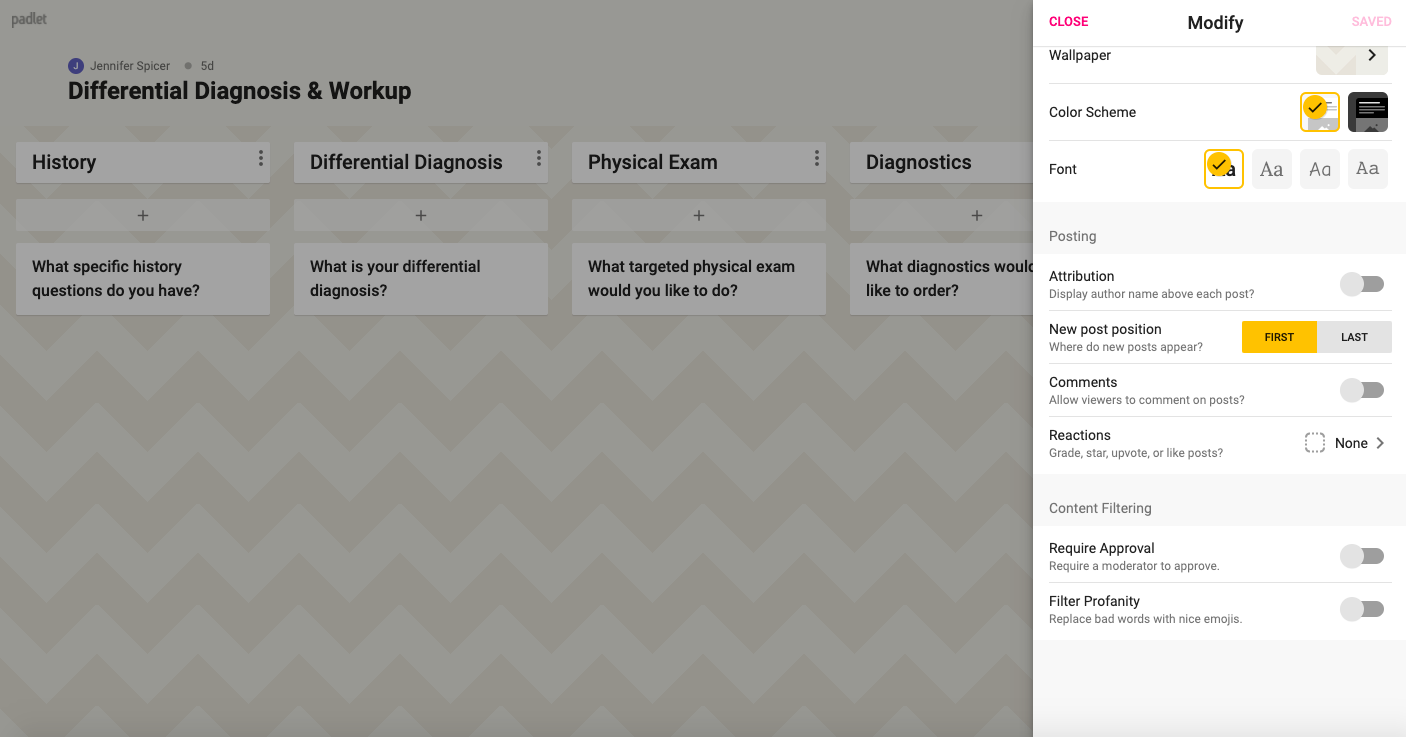


Then the facilitator should go to the previously created “Pros and Cons of Padlet” webpage and share that weblink with the participants. The participants should be asked to create a “post it” and list pros and cons of using Padlet.

PollEverywhere Setup and Demonstration

Prior to the session, the facilitator should go to the website [https://polleverywhere.com](https://www.polleverywhere.com/) and create an account. The facilitator can also create the poll that will be used in the session by going to the upper left corner of the website and clicking on the “Create” button:


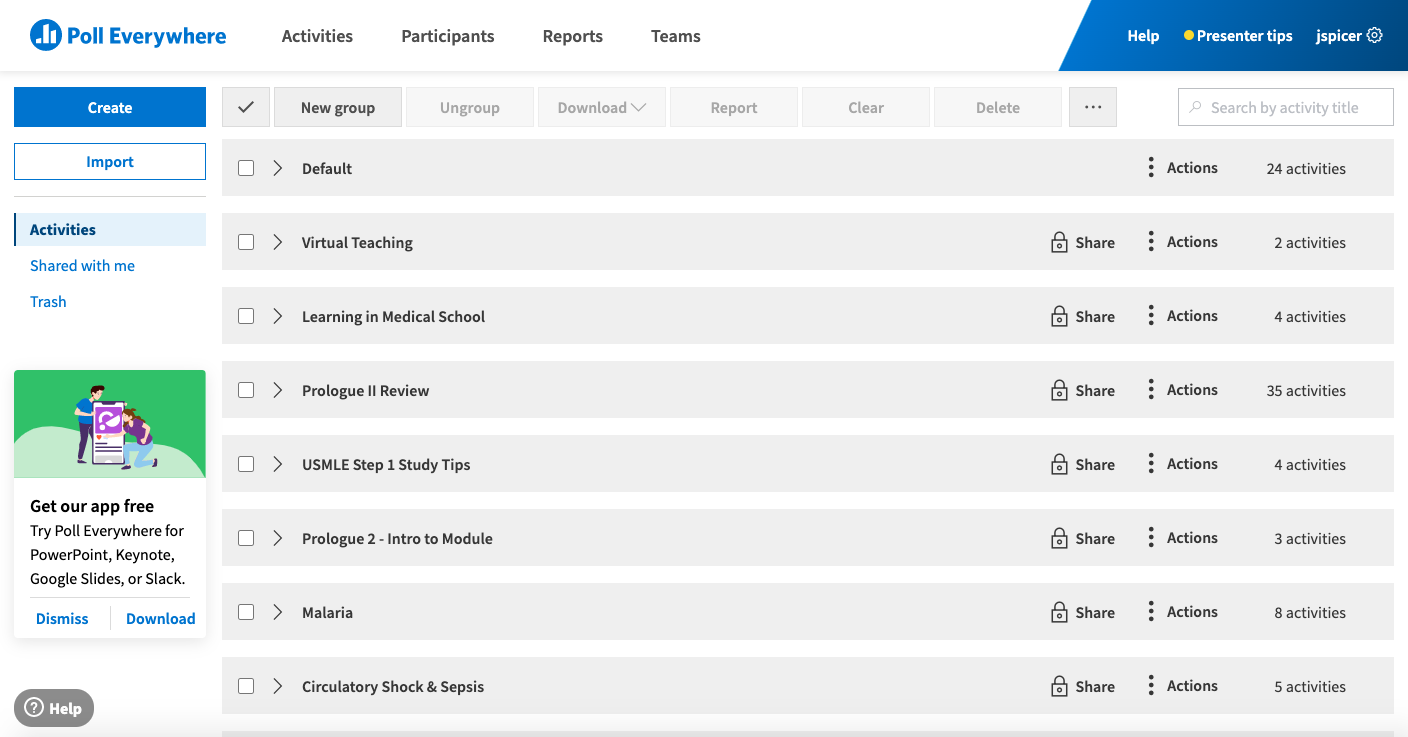


The following screen will appear, which shows options for creating different polls. The facilitator should select the “Upvote” option, and type in the question “What are the pros of PollEverywhere as compared to Zoom polling?”:


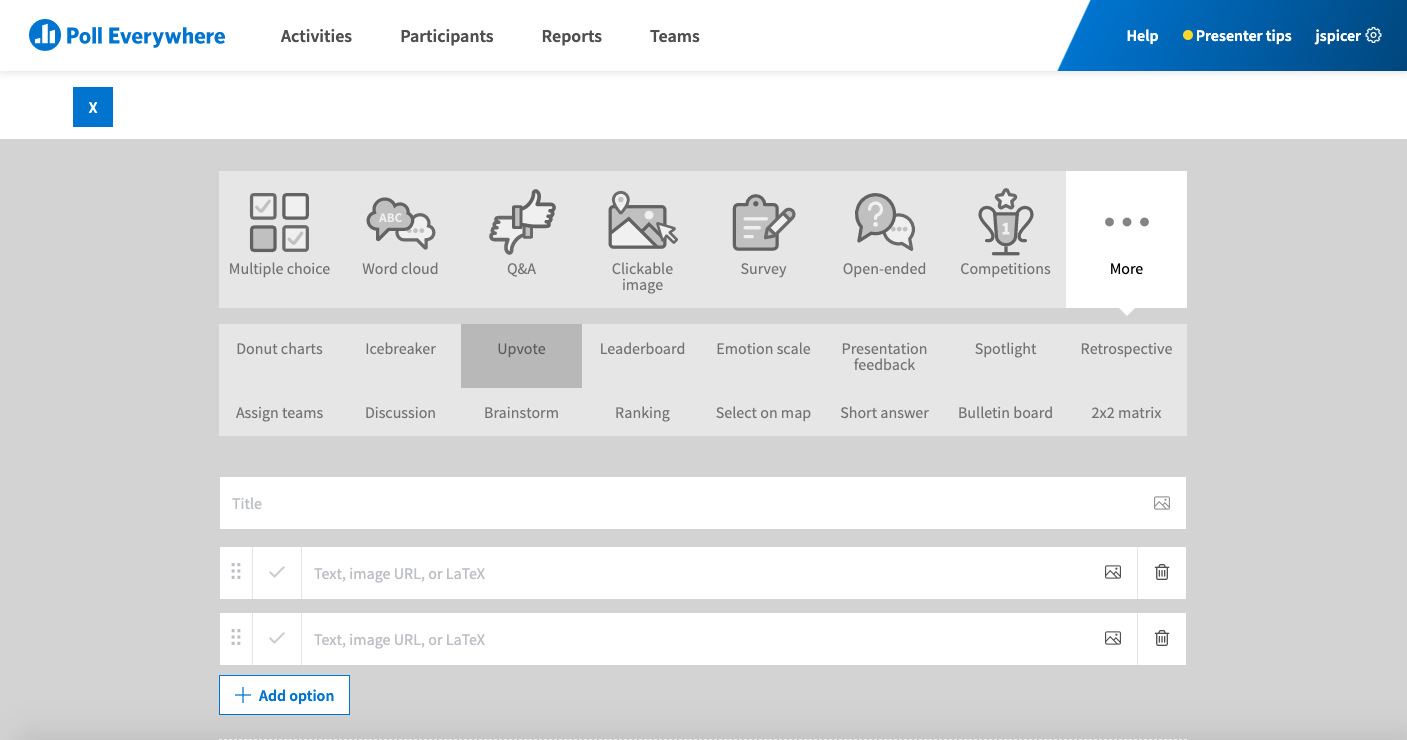


Once the poll is created, the facilitator can view the poll, as shown below. This is the screen that the facilitator should have open at the start of the session. The facilitator should make sure to click on the options to “Activate” the poll and “Show Now.” The link to share with the participants will be at the top of the poll (shown in the top gray banner in the image below, but will be personalized for the facilitator’s account).


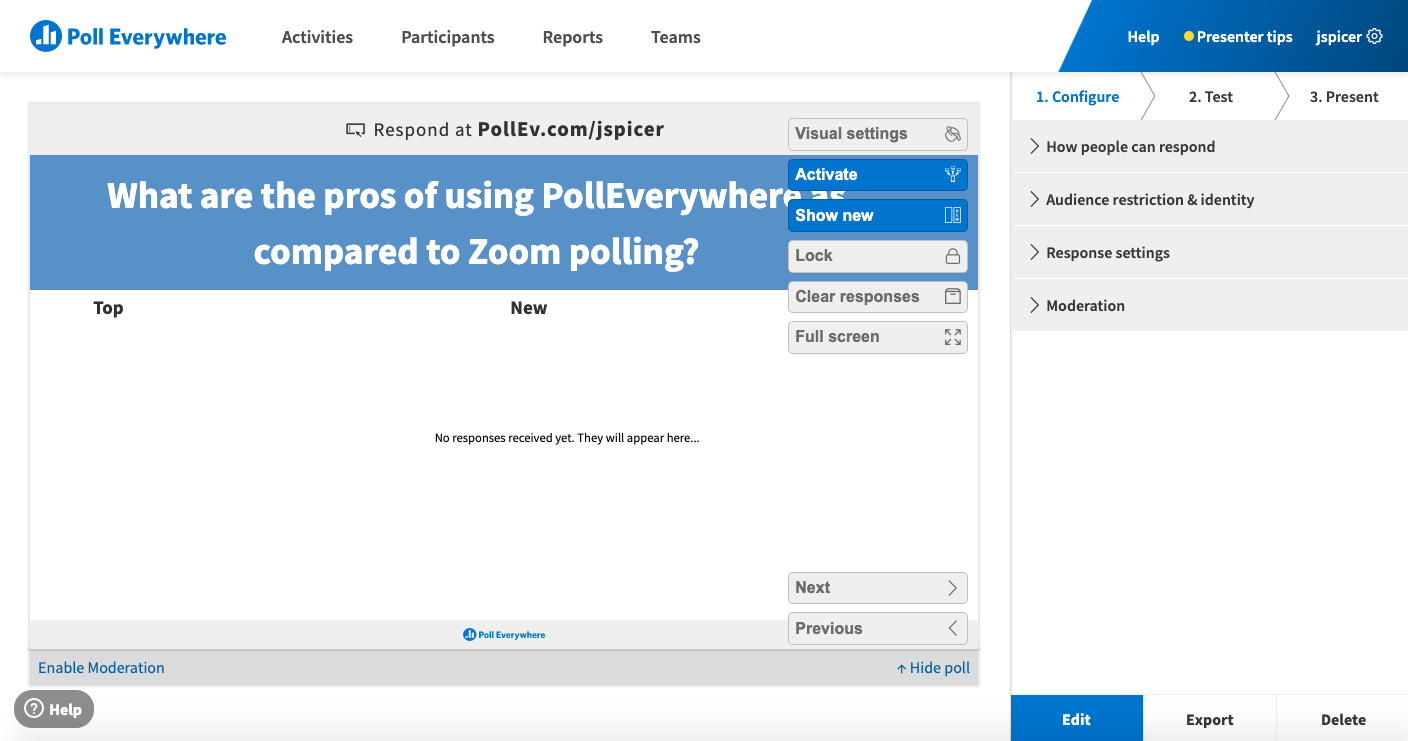

Supplement: Supplementary file 1 — Optional Readings.pptxInteractive Tools Worksheet.docxWorkshop Presentation.pptxFacilitator Guide Tech Demo.docxBreakout Session Worksheet.docxWorkshop Evaluation.docx [file mep_2374-8265.11126-s001.zip › D. Facilitator Guide Tech Demo.docx]
