# Supplementary material for: A Faculty Development Workshop for Planning and Implementing Interactive Virtual Case-Based Teaching
Source: MedEdPORTAL. 2021 Mar 17;17:11126. doi: 10.15766/mep_2374-8265.11126 (PMC7970636; doi:10.15766/mep_2374-8265.11126)
Supplement: Supplementary file 1 — Optional Readings.pptxInteractive Tools Worksheet.docxWorkshop Presentation.pptxFacilitator Guide Tech Demo.docxBreakout Session Worksheet.docxWorkshop Evaluation.docx [file mep_2374-8265.11126-s001.zip › E. Breakout Session Worksheet.docx]

Outlining Interactive Virtual Case-based Sessions

Using the tables below, outline which tools you would use to promote an interactive, virtual case-based session for each group.

**Small group** (8 students or residents)

| **Case Section** | **Tool** | **Rationale**  (i.e. why this tool rather than another one?) | **Breakout Rooms?**  (Yes or No) |
| --- | --- | --- | --- |
| Eliciting additional history |  |  |  |
| Forming a differential diagnosis |  |  |  |
| Choosing physical exam maneuvers |  |  |  |
| Requesting labs |  |  |  |
| Deciding on a diagnosis |  |  |  |
| Proposing a management plan |  |  |  |

**Large group** (50 students or residents)

| **Case Section** | **Tool** | **Rationale**  (i.e. why this tool rather than another one?) | **Breakout Rooms?**  (Yes or No) |
| --- | --- | --- | --- |
| Eliciting additional history |  |  |  |
| Forming a differential diagnosis |  |  |  |
| Choosing physical exam maneuvers |  |  |  |
| Requesting labs |  |  |  |
| Deciding on a diagnosis |  |  |  |
| Proposing a management plan |  |  |  |

Reflect on how each of the following impacts your choice of interactive tools.

- Anonymity:
- Class size:
- Learner level (i.e. students vs residents/fellows):
