# Supplementary material for: A Faculty Development Workshop for Planning and Implementing Interactive Virtual Case-Based Teaching
Source: MedEdPORTAL. 2021 Mar 17;17:11126. doi: 10.15766/mep_2374-8265.11126 (PMC7970636; doi:10.15766/mep_2374-8265.11126)
Supplement: Supplementary file 1 — Optional Readings.pptxInteractive Tools Worksheet.docxWorkshop Presentation.pptxFacilitator Guide Tech Demo.docxBreakout Session Worksheet.docxWorkshop Evaluation.docx [file mep_2374-8265.11126-s001.zip › F. Workshop Evaluation.docx]

**Appendix F: Workshop Evaluation**

|  | Strongly Disagree | Disagree | Neutral | Agree | Strongly Agree | N/A |
| --- | --- | --- | --- | --- | --- | --- |
| 1. The moderators provided clear and relevant learning objectives. |  |  |  |  |  |  |
| 1. The moderators presented relevant seminal articles. |  |  |  |  |  |  |
| 1. The moderators used appropriate reference materials. |  |  |  |  |  |  |
| 1. The moderators demonstrated adequate knowledge of the subject. |  |  |  |  |  |  |
| 1. The workshop was clear and organized. |  |  |  |  |  |  |
| 1. Following this workshop, I am more familiar with various virtual interactive tools used in online teaching. |  |  |  |  |  |  |
| 1. Following this workshop, I feel comfortable using various virtual interactive tools used in online teaching. |  |  |  |  |  |  |
| 1. Following this workshop, I plan to incorporate various virtual interactive tools used in online teaching. |  |  |  |  |  |  |

1. Identify the three most important concepts that you learned from this workshop.
2. Identify a question that was unanswered question by the workshop.
3. Identify areas or content in this workshop that require improvement.
